# Synthesis and Characterization of Symmetrical *N*-Heterocyclic Carbene Copper(II) Complexes—An Investigation of the Influence of Pyridinyl Substituents

**DOI:** 10.3390/molecules29153542

**Published:** 2024-07-27

**Authors:** Bhupendra Adhikari, Selvam Raju, Raymond Femi Awoyemi, Bruno Donnadieu, David O. Wipf, Sean L. Stokes, Joseph P. Emerson

**Affiliations:** Department of Chemistry, Mississippi State University, Starkville, MS 39762, USA

**Keywords:** *N*-heterocyclic carbene, symmetrical, copper(II)–NHC, pyridine, C–N cross coupling

## Abstract

Three new tridentate copper(II) *N*-heterocyclic carbene (NHC) complexes have been obtained and characterized with symmetrical C-4 substitutions on their pendent pyridine rings. Substitutions including methyl (Me), methoxy (OMe), and chloro (Cl) groups, which extend the library pincer Cu-NHC complexes under investigation, modify the impact of pyridinyl basicity on NCN pincer complexes. Both ligand precursors and copper(II) complexes are characterized using a range of techniques, including nuclear magnetic resonance (NMR) spectroscopy for ^1^H, ^13^C, ^31^P, and ^19^F nuclei, electrospray ionization mass spectrometry (ESI-MS), X-ray crystallography, cyclic voltammetry, and UV-Vis spectroscopy. The pyridine substitutions lead to minimal changes to bond lengths and angles in the X-ray crystal structures of these related complexes; there is a pronounced impact on the electrochemical behavior of both the ligand precursors and copper complexes in the solution. The substitution in the pyridinyl units of these complexes show an impact on the catalytic reactivity of these complexes as applied to a model C–N bond-forming reaction (CEL cross-coupling) under well-established conditions; however, this observation does not correlate to the expected change in basicity in these ligands.

## 1. Introduction

Since Arduengo’s groundbreaking work in 1991, stable *N*-heterocyclic carbenes (NHCs) have gained prominence, particularly as organometallic ligands [1]. Over the past three decades, NHCs have emerged as crucial contributors in coordination chemistry and catalysis [2]. Specifically, NHCs serve as versatile σ-donating ligands that rival other commonly used ligands like water-sensitive phosphines. Metal-NHC complexes have been shown to catalyze a plethora of chemical reactions including hydrogenation, metathesis, cross-coupling processes, and hydroformylation [3,4].

**Figure 1:**
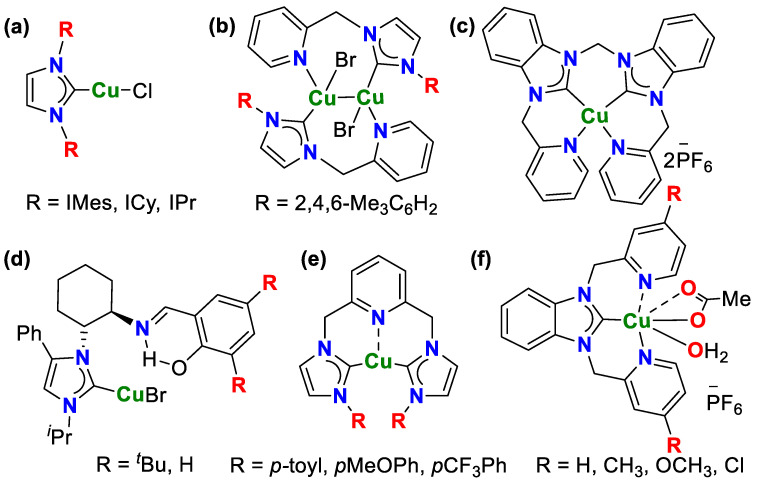
Cu-NHCs prepared using imidazolium salts with copper [5,6,7]. Complexes (**a**–**e**) in this figure represent selected copper NHC complexes previously reported in the literature. Complex (**f**) highlights this works focus.

It is generally accepted that a carbon donor of the NHC has a significant impact to the stability and electronic properties of these systems. The earliest examples of NHC complexes were unstable, monodentate coordination complexes. These early examples showed limited utility due in part to the free rotation in the NHC carbon—metal coordination mode, which allowed for an ensemble of conformations in the solution. Chelating NHC systems with bi- and tridentate binding modes were developed that added additional coordinating moieties into the ligand architecture affording more stable coordination modes (Figure 1) [5,6,7]. These flanking coordinating groups also contribute to the electronic tuning of the metal center [8,9], while controlling accessibility of the substrate to the catalytic metal ion [10,11,12]. Generally, the steric bulk of the NHC ligands manifest through the concept of percent buried volume and impart electronic effects that notably influence catalytic reactivity [13].

In more recent years, there has been a growing emphasis on developing catalytic systems that employ cost-effective metals such as copper (Cu), cobalt (Co), nickel (Ni), and iron (Fe) [14,15,16,17]. In some cases, the NHC complexes demonstrate stability even in the presence of air and moisture [18,19,20,21,22].

Cu-NHC species with the composition [Cu(NHC)(X)] (where X = OAc, I, Br, Cl) have demonstrated exceptional catalytic efficiency in numerous transformations [2]. These transformations include [3+2] cycloaddition reactions involving azides and alkynes, cross-coupling reactions, hydrosilylation, and carbonyl compound reduction. In 2010, Nolan et al. synthesized three comprehensive series of NHC-containing copper complexes [(NHC)CuCl], leading to the discovery of enhanced catalysts for ketone hydrosilylation and alkyne-azide 1,3-dipolar cycloaddition, revealing unique NHC ligand coordination modes [23]. This research distinctly illustrates the substantial advantages of employing precisely defined systems (Figure 1a). Eastham et al. demonstrated the straightforward preparation of pyridine-*N*-functionalized copper(I) carbene complexes and characterized the monomeric Cu^+^ imidazol-2-ylidene complex, featuring a Cu–C bond shorter than a typical Cu–C single bond [24]. By utilizing picolyl-*N*-functionalized carbene ligands with improved bite angles and backbone flexibility, they produced dimeric and polymeric materials (Figure 1b). Emerson and coworkers have developed an effective protocol facilitating the coupling of aromatic amines and arylboronic acids through the CEL coupling reaction, employing a tetradentate copper(II)–NHC complex that donates NCCN (Figure 1c). Furthermore, a tridentate copper(II)–NHC complex, distinguished by an NCN-type ligand originating from the precursor 1,3-bis(pyridine-2-ylmethyl)-1H-benzo[d]imidazole (bPymBI·PF_6_) was synthesized [25,26,27]. Douthwaite et al. described copper(II) complexes utilizing a chiral NHC precursor with an imidazol–phenoxyimine motif, exploring their potential in enantioselective catalysis [28]. This study reported the catalytic potential of their ligand through a tridentate coordination mode involving NHC–carbon, imine–nitrogen, and phenoxide–oxygen (CNO), including additional oxygen-coordinating groups like alkoxide and aryl oxide in the ligand (Figure 1d). In 2023, Tahsini and coworkers reported the synthesis and characterization of six novel copper(I)−CNC complexes. These complexes feature trifluoroethyl (TFE), phenyl, and aryl wings that are substituted in the *para* position with both electron-donating and electron-withdrawing groups, such as CF_3_, OCF_3_, and CH_3_ (Figure 1e) [20].

Building on these works, we report the synthesis of a family of a tridentate copper(II) –NHC complex characterized by an NCN-type ligand derived from the precursor 1,3-bis(pyridine-2-ylmethyl)-*1H*-benzo[*d*]imidazole ([bPymBI][PF_6_]) (Figure 1f) with different substituted pyridine moieties. This study examines how incorporating electron-withdrawing and electron-donating groups into the flanking pyridinyl units affects the structure and electronic properties of the resulting copper(II)–NHC complexes. Specifically, we modified and characterized our tridentate [bPymBI][PF_6_] ligand precursor, generating three new complexes with *para* substitutions in the pyridine rings, resulting in the cationic heteroleptic six-coordinate Cu–NHC complexes, denoted as Cu^2+^bPymBI-Me, Cu^2+^bPymBI-OMe, and Cu^2+^bPymBI-Cl. Together, this series of coordination compounds afford insight into the role pyridinyl donor properties/basicity have on the structure and electronic properties of the copper(II)–NHC complexes.

## 2. Results and Discussion

**Scheme 1:**
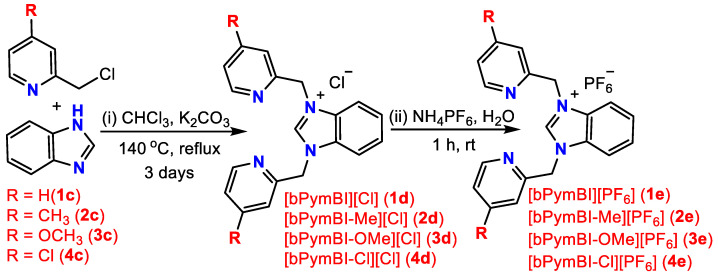
Schematic representation for the synthesis of NHC carbenes: (i) generation ligand generation, and (ii) counter anion exchange to PF_6_^−^ salt.

The synthesis of tridentate copper-NHC complexes followed a general procedure involving the generation of three new complexes (Scheme 1). In the first step, a tridentate NHC precursor ligand was synthesized by reacting 2.0 equivalents of picolyl chloride with 1.0 equivalent of benzimidazole. The resulting solution was heated to reflux in CHCl_3_ for 48 h, yielding the tridentate NHC-precursor chloride salt in 76–86% yield. Subsequently, the chloride counterion was exchanged with PF_6_^−^ to form compounds [bPymBI-Cl][PF_6_], [bPymBI-Me][PF_6_], and [bPymBI-OMe][PF_6_]. The precipitates were collected and dried under vacuum in a vacuum desiccator.

**Figure 2:**
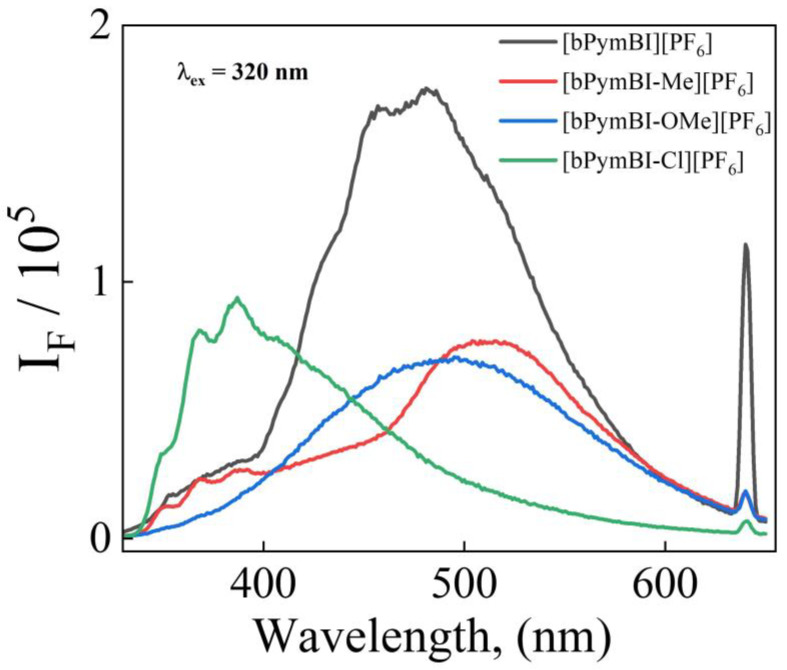
Fluorescence emission spectra of 5 mM [bPymBI][PF_6_] (black), [bPymBI-Me][PF_6_] (red) [bPymBI-OMe][PF_6_] (blue), and [bPymBI-Cl][PF_6_] (green) complexes in MeOH. Both excitation and emission slit widths were set to 2 nm with integration time of 0.3 s.

These ligand precursors salts were characterized using FT-IR, HR-MS, and ^1^H, ^13^C, ^19^F, and ^31^P NMR when appropriate. The ^1^J_CH_ NMR couplings of the carbene carbon precursor have been correlated with the σ-donor strength of the resulting NHC complex [29]. The ^1^J_CH_ NMR couplings were measured for the [bPymBI][PF_6_], [bPymBI-Me][PF_6_], [bPymBI-OMe][PF_6_], and [bPymBI-Cl][PF_6_] precursor salts and were found to be 222.0, 222.4 and 222.9, and 221.9Hz, respectively. This result shows that all ligand variants should demonstrate similar (relatively weak) carbene donor properties, allowing for the direct assessment of the impact of the flanking pyridinyl units. These precursors were also characterized with fluorescence spectroscopy. The excitation wavelength of 320 nm was chosen based on the complex’s maximum UV absorption peak, and fluorescence emission was subsequently collected (Figure 2). The presence of an electron-withdrawing group in [bPymBI-Cl][PF_6_] results in a blue shift associated with the fluorescence emission in this series of ligand salts, leading to an emission profile with several observable transitions centered at 387 nm. In contrast, the electron-releasing groups in [bPymBI-Me][PF_6_] and [bPymBI-OMe][PF_6_] induce a red shift, resulting in lower emission energies at 513 nm and 495 nm, respectively [30]. As expected, [bPymBI][PF_6_] exhibits an intermediate energy state with an emission peak at 481 nm. The combination of Cu(OAc)_2_ with the ligand precursor in equimolar proportions resulted in quenching the fluorescence signal associated with these complexes.

**Scheme 2:**
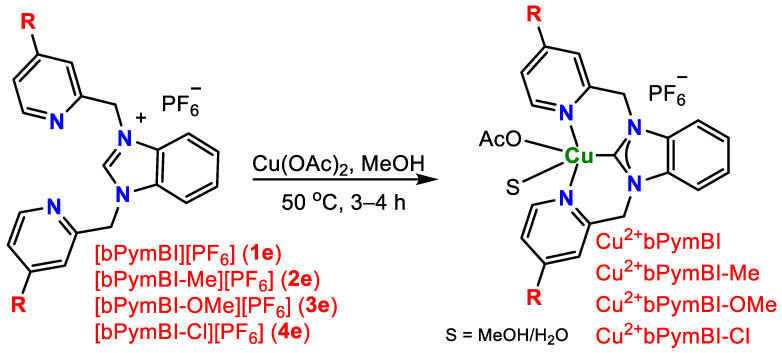
Schematic representation for the synthesis of Cu-NHC complexes.

The [NHC][PF_6_] precursor salts were combined with copper acetate in MeOH and stirred at 50 °C for 2–3 h, leading to the formation of dark blue precipitates corresponding to complexes Cu^2+^bPymBI-Cl, Cu^2+^bPymBI-Me, and Cu^2+^bPymBI-Ome (Scheme 2), with yields ranging from 75–90%. The NHC complexes were recrystallized, generating crystals suitable for X-ray crystallography. The recrystallized complexes were also characterized with ESI-MS, cyclic voltammetry, and a combination of other spectroscopic techniques, including UV-Vis and FT-IR spectroscopy, XRD, and TGA.

**Figure 3:**
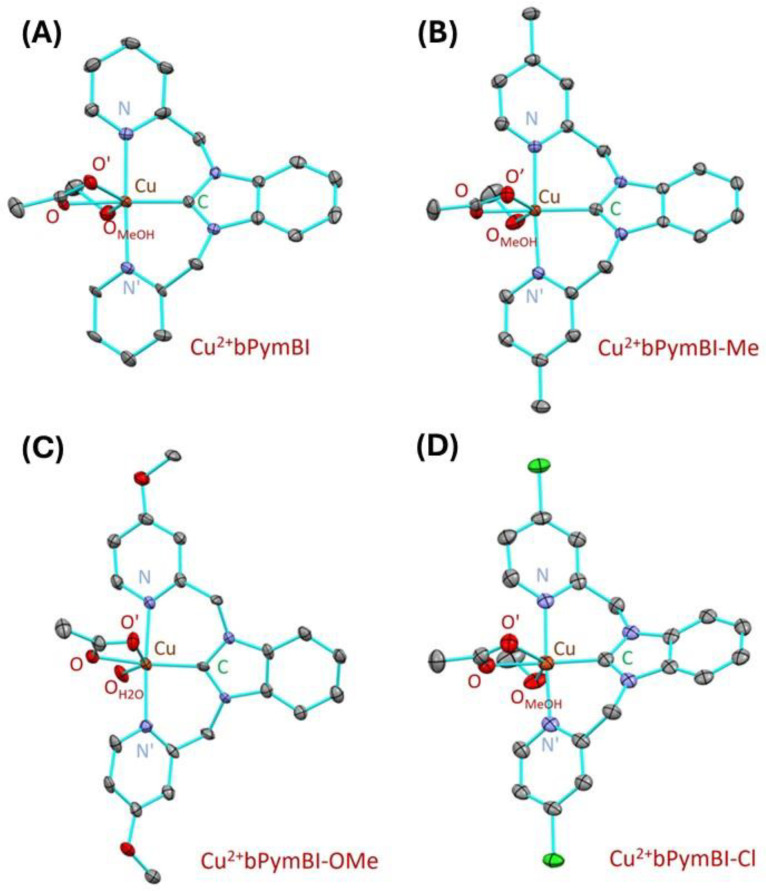
Crystal structures of copper complex (**A**) Cu^2+^bPymBI,^30^ (**B**) Cu^2+^bPymBI-Me (CCDC number: 2313922), (**C**) Cu^2+^bPymBI-Ome (CCDC number: 2313921), and (**D**) Cu^2+^bPymBI-Cl (CCDC number: 2313923), shown with 50% probability thermal ellipsoids. PF_6_^–^ ions and H-atoms are removed for clarity.

Single crystals of Cu^2+^bPymBI-Cl, Cu^2+^bPymBI-Me, and Cu^2+^bPymBI-OMe were grown through solvent evaporation using a blend of acetonitrile, dichloromethane, and methanol (2:1:1) yielding [CuNHC(solvent)(C_2_H_3_O_2_)][PF_6_]. These complexes are described as Cu^2+^NHC with anions and solvent-derived ligands omitted for clarity, where the generic NHC can be bPymBI, bPymBI-Me bPymBI-OMe, or bPymBI-Cl. Figure 3 illustrates the ORTEP representation of these complexes. Detailed crystal structure refinement data are available in the Supplementary Information (SI) provided in Tables S1–S3, along with the selected bond lengths and angles shown in Table 1. Within the crystallographic asymmetric units, each complex is accompanied by a hexafluorophosphate anion. The hexacoordination around the copper(II) center involves two nitrogen atoms and one carbon atom from the tridentate NHC ligand, along with three oxygen atoms from the solvent and acetate units. Curiously, these complexes exhibit similar bond lengths and angles to those previously discussed for complex Cu^2+^bPymBI (refer to Table 1) [26]. Based on the ^1^J_CH_ NMR couplings reported above, it was expected that the Cu–C bonds would be all similar due to their common σ-donation strength, but it was unexpected that changing the basicity of the pyridinyl units also had minimal structural impact to the measured Cu–N bond lengths. A similar trend was observed in a related study, where substituents in the para position of the pyridinyl units in a NNN-type pincer ligands, resulted in negligible structural differences. In this report, the related Cu–N bond lengths in the pyridyl ring were 1.926, 1.939, and 1.949 Å for substituents ranging from a –OH, to –H, to –Cl, respectively [31].

The equatorial plane of the distorted octahedral coordination mode in these complexes is occupied by two nitrogen atoms, one carbon atom from the tridentate NHC ligands, and one oxygen atom from the acetate ion. Meanwhile, the longer axial positions are filled by other oxygen atoms from methanol or water and acetate, as depicted in Figure 3. This axial elongation aligns with the Jahn–Teller distortion [30], indicating the labile nature of the axial ligands. The geometries of complexes Cu^2+^bPymBI-Cl, Cu^2+^bPymBI-Me, and Cu^2+^bPymBI-OMe are proposed to be tetragonally distorted octahedral. Support for this notion is based on bond distance analysis where Cu^2+^bPymBI-Me: R_in_ = (2.059 + 2.094 + 1.966 + 1.932 Å)/4; R_out_ = (2.333 + 1.966 Å)/2, Cu^2+^bPymBI-OMe: R_in_ = (1.920 + 2.058 + 2.086 + 2.271 Å)/4; R_out_ = (1.982 + 2.271 Å)/2, Cu^2+^bPymBI-Cl: R_in_ = (1.964 + 2.101 + 2.051 + 1.937 Å)/4; R_out_ = (1.964 + 2.308 Å)/2] T values = R_in_/R_out_ = 0.9365, 0.9800, and 0.9425, respectively. The confirmation of these distortions in the octahedral structures is further supported by the bond angle of Cu^2+^bPymBI-Me: ∠N–Cu–N’ = 176.49(5); ∠O–Cu–C = 161.73(6), Cu^2+^bPymBI-OMe: ∠N–Cu–N’ = 176.4(3); ∠O–Cu–C = 160.6(3), Cu^2+^bPymBI-Cl: ∠N–Cu–N’ = 176.62(14); and ∠O–Cu–C = 162.75(16), respectively. These values suggest that the equatorial plane is not a square planar around the copper(II) center. The relatively short bond lengths of Cu–C in the NHC complexes (Cu^2+^bPymBI-Me: 1.937(4), Cu^2+^bPymBI-OMe: 1.920(8), Cu^2+^bPymBI-Cl: 1.937(4) Å) correspond to *σ* donation and *π* back-bonding of the metal carbene, contributing to the complexes’ stability [29].

**Figure 4:**
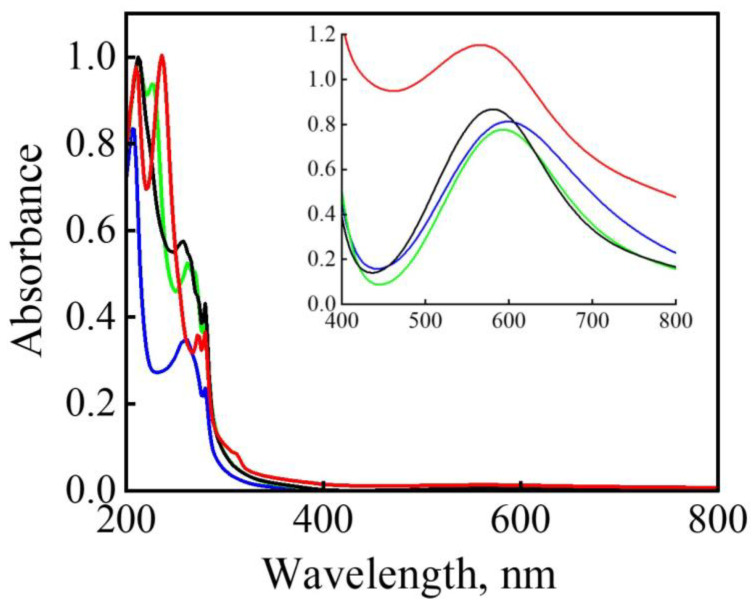
UV-Vis absorption spectra of the Cu^2+^bPymBI (blue), Cu^2+^bPymBI-Me (black), Cu^2+^bPymBI-OMe (green), and Cu^2+^bPymBI-Cl (red) at 25 μM in acetonitrile. Inset shows absorption spectra of all complexes at 5 mM in acetonitrile, highlighting the low-intensity *d*–*d* transition.

The solution UV-Vis spectra were collected for complexes Cu^2+^bPymBI, Cu^2+^bPymBI-Me, Cu^2+^bPymBI-OMe, and Cu^2+^bPymBI-Cl in CH_3_CN (Figure 4). All three complexes reveal notably higher energy d-d transitions (Cu^2+^bPymBI-Me: λ_max_ = 582 nm, ε_582_ 172 M^−1^cm^−1^; Cu^2+^bPymBI-OMe: λ_max_ = 576 nm, ε_576_ 199 M^−1^ cm^−1^; Cu^2+^bPymBI-Cl: λ_max_ = 598 nm, ε_598_ 154 M^−1^ cm^−1^) in compared to Cu(OAc)_2_, highlighting the strong-field nature of the NHC system, which has been observed previously [32,33]. The λ_max_ associated with the d-d transitions in these complexes does shift predictably where electron withdrawing groups blue shift this band, and where donating groups red shift this feature. Furthermore, there are several high-energy absorption features likely associated with the ligand-based π-π* transitions or charge transfer transitions in the range of 260–315 nm.

**Figure 5:**
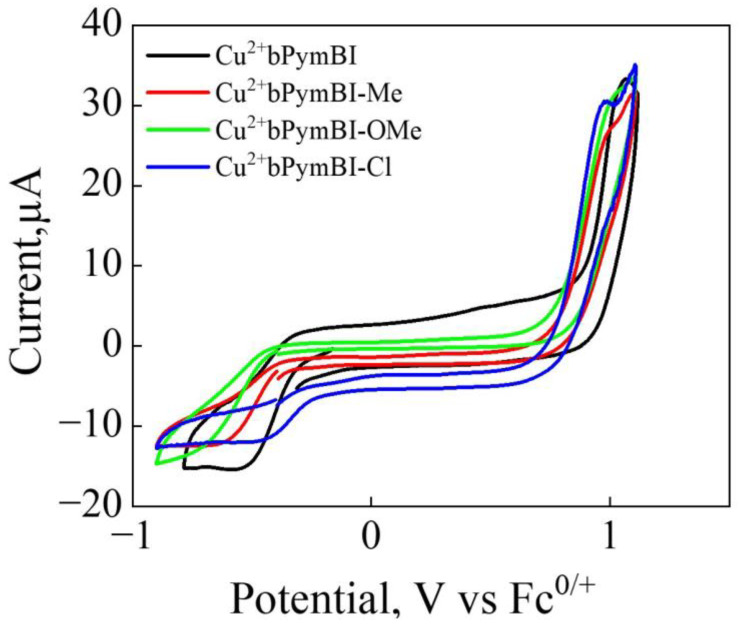
The cyclic voltammograms of 1 mM complexes Cu^2+^bPymBI (black = H, red = Me, green = OMe, and blue = Cl) were measured in 100 mM NBu_4_PF_6_/CH_3_CN. Scan Rate: 100 mV/s, GCE working electrode, and graphite rod auxiliary electrode.

The voltammograms for Cu^2+^bPymBI, Cu^2+^bPymBI-Me, Cu^2+^bPymBI-OMe and Cu^2+^bPymBI-Cl, in a 0.1 M NBu_4_PF_6_/CH_3_CN solution are illustrated in Figure 5. Cu^2+^bPymBI (depicted in Figure 5, black trace), previously documented by Emerson and co-workers [26,27], and the different Cu^2+^NHC complexes in aqueous solutions, show similar irreversible anodic (E_ox_) and cathodic (E_red_) peak patterns to the three new complexes (Figure 5 red, green, and blue traces). The irreversible reduction peak at −0.785, −0.582 V, −0.701 V, and −0.452 V vs. Fc+/0, respectively, is present in complexes Cu^2+^bPymBI, Cu^2+^bPymBI-Me, Cu^2+^bPymBI-OMe, and Cu^2+^bPymBI-Cl. The anodic peaks were recorded at 0.985 V, 1.034 V, and 0.969 V vs. Fc+/0 for complexes Cu^2+^bPymBI-Me, Cu^2+^bPymBI-OMe, and Cu^2+^bPymBI-Cl, respectively. 

The low standard redox potential of 0.142 V observed for the neutral Cu^2+^bPymBI complex indicates a low energy bar for the observed redox process in cyclic voltammetry. Remarkably, with different species, a variation in potential is observed, measuring 0.202 V, 0.167 V, and 0.254 V for Cu^2+^bPymBI-Me, Cu^2+^bPymBI-OMe, and Cu^2+^bPymBI-Cl, respectively. This implies the sensitivity to the chemical ambiance with the redox behavior, indicating the influence of substituents on the copper-bPymBI complexes. Additionally, the blue-shifted fluorescence emission band was observed in the [bPymBI-Cl][PF_6_] ligand precursor, and may be attributed to its relatively poor π-donating nature compared to other ligands [34].

Finally, all four complexes were screened as CEL cross-coupling agents utilizing the model reaction of imidazole and arylboronic acid under previously reported conditions [27]. Table 2 shows the influence of electron-donating and withdrawing groups on the yields of the C–N arylation reaction. While all the complexes displayed activity, low reaction yields were observed with electron-donating substituents on the pyridine units. This result indicates that at least one of the key mechanistic steps in the CEL reaction is impacted by the pyridinyl donor properties of these complex. The classical oxidative addition and reductive elimination steps associated with the CEL coupling reaction are likely steps that changes in lability and pyridinyl coordination impact. The magnitude of the change in reactivity was unexpected when compared to all other characterization data collected where the donating ability of the NHC dominates the electronics of these complexes. One possibility is that the basicity of the pyridine units plays a key role in the solution dynamics associated with these complexes. For example, the hemilability of traditional donor groups can influence catalysis in metal-NHC complexes [35,36]. In this case, it seems that the more basic pyridine units could be contributing to stronger or less labile dative bonding interactions with the metal ions. Although we normally consider two adjacent coordination sites sufficient to support CEL C–N bond-forming reactions [27], perhaps in this case, there is a marked catalytic benefit to a more dynamic coordination environment. The air-dependent oxidative catalyst recycling step is unlikely effected by pyridinyl donation, where the redox potentials of all complexes were measured to be highly similar.

Together, the solution data showcases that there are subtle differences in the electronic structure and perhaps the lability of the flanking pyridinyl units in this series of copper(II)–NHC complexes. There are notable effects to the d-d transitions, redox potential, and reactivity that follow inductive and resonance effect trends, which arise from the subtle pyridinyl donor properties in the soluble form of this complex. In addition, hyperconjugation may play a minor yet observable role in the behavior of alkyl substituents. The literature examples highlight the predominance of the inductive effect in the substitution of a chlorine (–Cl) group, which results in a decrease in the basicity of the pyridine ring. Conversely, introducing a methyl (–CH_3_) group increases the basicity of pyridine. A similar trend is seen with the methoxy (–OCH_3_) group, where this strong effect outweighs the inductive effect, leading to enhanced basicity [37]. These observations are directly opposed to the solid phase, crystallographic data that suggest minimal differences in the copper(II) coordination environment. In these copper(II)–NHC complexes, where crystal packing forces that minimize differences to the coordination environment must dominate other more subtle interactions induced by the pyridinyl coordination. 

## 3. Conclusions

In an effort to design NHC complexes to study the impact of electronic and resonance effects of flanking pyridinyl units on polydentate copper(II)–NHC systems, three new Cu-tridentate NHC complexes were synthesized and characterized. These complexes incorporate 2,3-dihydro-*1H*-benzo[*d*]imidazole-linked (NHC) ligands with 4-substituted pyridin-2-ylmethyl arms. X-ray crystallographic analysis of this series of mononuclear structures for Cu^2+^ NHC complexes displayed a common six-coordinated bonding mode between the copper centers and their NHC ligands. Minimal structural differences were observed in the coordination geometry in this series of copper(II)–NHC complexes based on X-ray crystallographic analysis, where both bond lengths and bond angles in all complexes are congruent. In the solution, the copper(II)–NHC complexes demonstrate the predictable strong σ-donation of the carbene unit which shifts the physical properties of the copper(II) center significantly from those of traditional copper(II) systems. However, this series of complexes also showcase the physical properties are impacted by the donor properties of the flanking pyridinyl units, which afford subtle but predicable perpetuation of the electronic structure and physical properties of the copper(II) center. The solution reactivity of these complexes toward Chan–Evans–Lam C-N cross-coupling reactions also showcases significant difference in reaction productivity that correlates to the electronic effects present in the pyridinyl units. In summary, electronic effects on pendant ligands can modulate the physical properties of Cu–NHC complexes in solutions that are not obvious from crystallographic analysis. It is unclear how the dynamic, solution structure of copper(II)–NHC complexes differ from their solid-state structures, but it is clear that these subtle effects can modulate reactivity of these systems.

## 4. Experimental Methods

Benzimidazole, picolinic acid, picolinic esters, picolyl chloride (TCI), ammonium hexafluorophosphate, copper (II) acetate (Alfa Aesar, Haverhill, MA, USA), and potassium carbonate were used as received. All reagents used for this work were of analytical grade and were used as received. The solvents used were of HPLC grade and were obtained from Fisher Scientific (Bridgewater, NJ, USA). All the products and ligand species were characterized by melting points (m.p), ^1^H-NMR, ^13^C-NMR, mass spectra, and infrared spectra (IR). Melting points were measured on an Electrothermal MEL-TEMP melting point apparatus; IR were recorded on a BRUKER spectrometer; ^1^H-NMR and ^13^C-NMR spectra were obtained on Agilient-500 MHz; and chemical shifts were reported in parts per million (ppm, *δ*), with CHCl_3_ as a reference. Proton coupling patterns are described as singlet (s), doublet (d), triplet (t), triplet of doublet (td), doublet of doublet (dd), and multiplet (m); coupling constants (J) are quoted in Hz. Carbon-13 nuclear magnetic resonance (^13^C-NMR) data were acquired at 100 MHz. The water used was initially deionized using a reverse osmosis system. All of the used-as-purchased chemicals were used without further purification. All of the salt products were purified using flash column chromatography using SiliaFlash^Ⓡ^ P60 (230–400 mesh) silica gels. All of the heating reactions were carried out using an oil bath equipped with a digital temperature controller.

**Procedure for the synthesis of methyl picolinate derivatives**


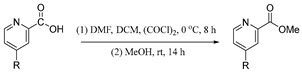


Picolinic acid derivative 1.0 equivalent (12.7 mmol), oxalyl chloride 3.5 equivalent (38.1 mmol), and a catalytic amount (5 drops) of DMF were combined with 50 mL of DCM in a round-bottom flask at 0 °C and stirred for 8 h under N_2_ atmosphere. The solvent was then removed using rotary evaporation at reduced pressure. The residue was treated with 20 mL of MeOH and stirred at RT for 14 h to achieve conversion of the respective esters. 

**Procedure for the synthesis of pyridin-2-ylmethanol derivatives**


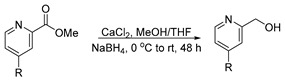


The 1.0 equivalent (8.8 mmol) of esters were reduced to alcohols by loading the 5.0 equivalent (43.8 mmol) of CaCl_2_ in 100 mL MeOH:THF (6:3.5) solvent at 0 °C to RT, followed by the addition of the 3.0 equivalent (26.3 mmol) of NaBH_4_ in three installments. The same addition was performed after 24 h, maintaining 0 °C to RT for more than 24 h to obtain the alcohol. 

**Procedure for the synthesis of 2-(chloromethyl)pyridine derivatives**


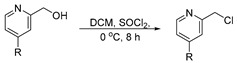


Pyridin-2-ylmethanol derivative 1 equivalent (6.9 mmol), and thionyl chloride 2.5 equivalent (17.5 mmol), was mixed with 50 mL of DCM in a round bottom flask at 0 °C and stirred for 8 h under N_2_ atmosphere. The solvent was then removed using rotary evaporation at reduced pressure. The residue was treated with *n*-hexane to obtain the precipitate/sticky liquid. 

**Procedure for the synthesis of NHC precursor ligand**


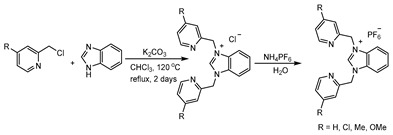


The mixture of 2.0 equivalent (3.4 mmol) of 2-picolyl chloride derivative, 1.0 equivalent (1.7 mmol) of benzimidazole, and 5.0 equivalent (8.5 mmol) of potassium carbonate were mixed with the CHCl_3_ solvent in a pressure tube and refluxed for 48 h at 120 °C. The compound was filtered, and the filtrate-containing solvent was removed completely under reduced pressure using rotary vaporization. The residue after rotary vaporization was again dissolved in DCM and dried over MgSO_4_ and the saturated complex was treated with *n*-hexane to obtain brown solid. The chloride salt of the ligand (1 equivalent, 0.5 mmol) was dissolved in the minimum amount of water followed by the addition of the 5.0 equivalent (2.5mmol) of ammonium hexafluorophosphate to obtain the brown-colored complex, and was dried under vacuum. 

**1,3-bis((4-chloropyridin-2-yl)methyl)-1*H*-benzo[*d*]imidazol-3-ium chloride (1d)**

Yield 32% (220 mg), Brown solid, m.p: 140–142 °C; R*_f_*: 0.25 (DCM:EA:MeOH; 7:3:0.5); ^1^H NMR (500 MHz, DMSO-d_6_) *δ*: 10.26 (s, 1H), 8.49 (d, *J* = 5.0 Hz, 2H), 8.01–7.99 (m, 2H), 7.91 (s, 2H), 7.66–7.64 (m, 2H), 7.57 (t, *J* = 5.0 Hz, 2H), 6.06 (s, 4H) ppm; ^13^C NMR (125 MHz, DMSO-d_6_) *δ*: 195.6, 151.5, 144.7, 144.3, 131.7, 127.3, 124.3, 123.4, 114.5, 50.9 ppm. FT-IR (KBr) ṽ (cm^−1^) 1580, 1510, 1430, 1375, 1280; HRMS (ESI^+^), calcd for C_19_H_15_Cl_3_N_4_ [M–Cl]^+^ 369.0668 found 369.0684.

**1,3-bis((4-chloropyridin-2-yl)methyl)-1*H*-benzo[*d*]imidazol-3-ium hexafluorophosphate (1e)**

Yield 94% (242 mg), Brown solid, m.p: 138–140 °C; R*_f_*: 0.67 (DCM:EA:MeOH; 7:3:0.5); ^1^H NMR (500 MHz, DMSO-d_6_) *δ*: 9.61 (s, 1H), 8.42 (s, 2H), 7.99 (s, 2H), 7.59 (s, 4H), 7.30 (s, 2H), 5.76 (s, 4H) ppm; ^13^C NMR (125 MHz, DMSO-d_6_) *δ*: 195.5, 151.5, 144.7, 144.4, 131.7, 127.4, 124.3, 123.4, 114.5, 50.9 ppm. ^19^F NMR (470 MHz, DMSO-d_6_) *δ*: −70.13 (d, *J* = 709.70 Hz) ppm; ^31^P NMR (202 MHz, DMSO-d_6_) *δ*: -144.19 (septet, *J* = 710.43 Hz) ppm; FT-IR (KBr) ṽ (cm^−1^) 1580, 1510, 1430, 1375, 1280; HRMS (ESI^+^), calcd for C_19_H_15_Cl_2_F_6_N_4_P [M–PF_6_]^+^ 369.0668 found 369.0684.

**1,3-bis((4-methylpyridin-2-yl)methyl)-1*H*-benzo[*d*]imidazol-3-ium chloride (2d)**

Yield 41% (240 mg), Faded brown solid, m.p: 201–203 °C; R*_f_*: 0.08 (DCM:EA:MeOH; 7:3:0.5); ^1^H NMR (500 MHz, CDCl_3_) *δ*: 11.66 (s, 1H), 8.25 (s, 2H), 7.7 (s, 2H), 7.53 (d, *J* = 5.0 Hz, 4H), 6.97 (s, 2H), 5.91 (s, 4H) 2.25 (s, 6H) ppm; ^13^C NMR (125 MHz, CDCl_3_) *δ*: 152.4, 149.3, 149.1, 143.9, 131.9, 126.7, 124.8, 124.3, 114.2, 52.6, 20.9 ppm. FT-IR (KBr) ṽ (cm^−1^) 1580, 1510, 1430, 1375, 1280; HRMS (ESI^+^), calcd for C_21_H_21_ClN_4_ [M–Cl]^+^ 329.1761 found 329.1751.

**1,3-bis((4-methylpyridin-2-yl)methyl)-1*H*-benzo[*d*]imidazol-3-ium hexafluorophosphate (2e)**

Yield 97% (230 mg), brown solid, m.p: 153–155 °C; R*_f_*: 0.46 (DCM:EA:MeOH; 7:3:0.5); ^1^H NMR (500 MHz, CDCl_3_) *δ*: 9.48 (s, 1H), 8.36 (d, *J* = 5, 2H), 7.83 (s, 2H), 7.55 (d, *J* = 5.0 Hz, 2H), 7.44 (s, 2H), 7.08 (d, *J* = 10, 2H), 5.68 (s, 4H), 2.36 (s, 6H) ppm; ^13^C NMR (125 MHz, CDCl_3_) *δ*: 151.9, 149.6, 149.5, 142.1, 131.7, 127.3, 125.1, 124.4, 114.1, 52.8, 20.9 ppm. ^19^F NMR (470 MHz, DMSO-d_6_) *δ*: −70.13 (d, *J* = 710.28 Hz) ppm; ^31^P NMR (202 MHz, DMSO-d_6_) *δ*: −144.19 (septet, *J* = 712.46 Hz) ppm; FT-IR (KBr) ṽ (cm^−1^) 1580, 1510, 1430, 1375, 1280; HRMS (ESI^+^), calcd for C_21_H_21_F_6_N_4_P [M–PF_6_]^+^ 329.1761 found 329.1751.

**1,3-bis((4-methoxypyridin-2-yl)methyl)-1*H*-benzo[*d*]imidazol-3-ium chloride (3d)**

Yield 29% (200 mg), Brown solid, m.p: 169–171 °C; R*_f_*: 0.09 (DCM:EA:MeOH; 7:3:0.5); ^1^H NMR (500 MHz, CDCl_3_) *δ*: 11.86 (s, 1H), 8.28 (d, *J* = 5.0 Hz, 2H), 7.91–7.89 (m, 2H), 7.54–7.52 (m, 4H), 6.75 (t, *J* = 5.0 Hz, 2H), 5.92 (s, 4H), 3.83 (s, 6H) ppm; ^13^C NMR (125 MHz, CDCl_3_) *δ*: 166.9, 154.2, 150.6, 144.0, 131.6, 126.9, 114.2, 110.9, 109.4, 55.9, 52.8 ppm. FT-IR (KBr) ṽ (cm^−1^) 1580, 1510, 1430, 1375, 1280; HRMS (ESI^+^), calcd for C_21_H_21_ClN_4_O_2_ [M–Cl]^+^ 361.1659 found 361.1684.

**1,3-bis((4-methoxypyridin-2-yl)methyl)-1*H*-benzo[*d*]imidazol-3-ium hexafluorophosphate (3e)**

Yield 95% (240 mg), Brown solid, m.p: 168–170 °C; R*_f_*: 0.51 (DCM:EA:MeOH; 7:3:0.5); ^1^H NMR (500 MHz, DMSO-d_6_) *δ*: 10.08 (s, 1H), 8.31 (d, *J* = 5.0 Hz, 2H), 7.96–7.95 (m, 2H), 7.64–7.62 (m, 2H), 7.30 (d, *J* = 5.0 Hz, 2H), 6.98–6.97 (m, 2H) 5.90 (s, 4H), 3.87 (s, 6H) ppm; ^13^C NMR (125 MHz, DMSO-d_6_) *δ*: 166.9, 154.9, 151.5, 144.4, 131.7, 127.2, 114.2, 110.0, 109.7, 96.1, 51.4 ppm. ^19^F NMR (470 MHz, DMSO-d_6_) *δ*: −70.14 (d, *J* = 710.28 Hz) ppm; ^31^P NMR (202 MHz, DMSO-d_6_) *δ*: −144.20 (septet, *J* = 711.04Hz) ppm; FT-IR (KBr) ṽ (cm^−1^) 1580, 1510, 1430, 1375, 1280; HRMS (ESI^+^), calcd for C_21_H_21_F_6_N_4_O_2_P [M–PF_6_]^+^ 361.1659 found 361.1684.

**1,3-bis(pyridin-2-ylmethyl)-1*H*-benzo[*d*]imidazol-3-ium chloride (4d)**

Yield 40% (228 mg), Brown solid, m.p: 112–114 °C; R*_f_*: 0.51 (DCM:EA:MeOH; 7:3:0.5); ^1^H NMR (500 MHz, DMSO-d_6_) *δ*: 10.22 (s, 1H), 8.50 (d, *J* = 5.0 Hz, 2H), 7.97–7.86 (m, 2H), 7.92–7.91 (m, 2H), 7.69 (d, *J* = 10.0 Hz, 2H), 7.63–7.61 (m,2H), 7.40–7.38 (m,2H), 3.35 (s, 4H), ppm; ^13^C NMR (125 MHz, DMSO-d_6_) *δ*: 153.5, 150.1, 144.5, 138.1, 131.7, 127.2, 124.2, 123.2, 114.5, 51.4, ppm.

**1,3-bis(pyridin-2-ylmethyl)-1*H*-benzo[*d*]imidazol-3-ium chloride (4e)**

Yield 95% (211 mg), Brown solid, m.p: 168–170 °C; R*_f_*: 0.51 (DCM:EA:MeOH; 7:3:0.5); ^1^H NMR (500 MHz, DMSO-d_6_) *δ*: 10.12 (s, 1H), 8.51 (d, *J* = 5.0 Hz, 2H), 7.97–7.95 (m, 2H), 7.94–7.91 (m, 2H), 7.67 (d, *J* = 5.0 Hz, 2H), 7.64–7.63 (m, 2H), 7.41–7.38 (t, J = 10.0 Hz, 2H), 6.00 (s, 4H), ppm; ^13^C NMR (125 MHz, DMSO-d_6_) *δ*: 153.5, 150.1, 144.5, 138.1, 131.7, 127.2, 124.2, 123.2, 114.5, 51.4, ppm. ^19^F NMR (470 MHz, DMSO-d_6_) *δ*: −70.11 (d, *J* = 710.3 Hz) ppm; ^31^P NMR (202 MHz, DMSO-d_6_) *δ*: −144.16 (septet, *J* = 712.5 Hz) ppm.

**Procedure for the synthesis of Cu^2+^bPymBI-R complex**


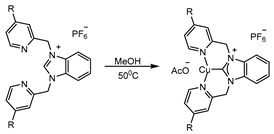


The 1.0 equivalent of the hexafluorophosphate version of the ligand was mixed with the 1.0 equivalent of Cu(OAc)_2_ in minimum MeOH (5.0 mL) in a flask and stirred for 2 h at 50 °C. A blue-colored precipitation was obtained, and was separated using filtration followed by washing with the MeOH.

## Figures and Tables

**Table 1:** Selected bond lengths (Å) for Cu^2+^bPymBI, Cu^2+^bPymBI-Me, Cu^2+^bPymBI-OMe, and Cu^2+^bPymBI-Cl.

Bond (Å)	Cu^2+^bPymBI [26]	Cu^2+^bPymBI-Me	Cu^2+^bPymBI-OMe	Cu^2+^bPymBI-Cl
Cu–C	1.935(8)	1.937(4)	1.920(8)	1.937(4)
Cu–N	2.112(7)	2.101(4)	2.058(6)	2.101(4)
Cu–N′	2.066(7)	2.057(4)	2.086(6)	2.057(4)
Cu–O	1.975(5)	2.308(3)	1.982(5)	2.308(3)
Cu–O′	2.758	2.308(3)	1.982(5)	2.308(3)
Cu–O_W/M_	2.287(6)	1.964(3)	2.271(6)	1.964(3)

**Table 2:** CEL cross-coupling reaction using [Cu(NHC)]^2+^ catalyst ^a^.

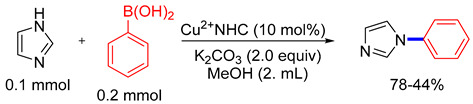		
No	[Cu^2+^(NHC)] Catalyst	Yield (3a%) ^b^
1	Cu^2+^bPymBI	78
2	Cu^2+^bPymBI-Me	44
3	Cu^2+^bPymBI-OMe	47
4	Cu^2+^bPymBI-Cl	65

^a^ Reaction conditions: **1a** (0.1 mmol), **2a** (0.2 mmol), [Cu(NHC)]^2+^ (10 mol%), and K_2_CO_3_ (0.2 mmol) in 2.0 mL of methanol at 70 °C for 24 h exposed to open air and then closed. ^b^ The reaction yields were calculated using ^1^H-NMR spectroscopy, using mesitylene as an internal standard.

## Data Availability

Data are available upon request.

## Supplementary Materials

The following supporting information can be downloaded at: https://www.mdpi.com/article/10.3390/molecules29153542/s1. References [25,26,38,39,40,41,42] are cited in Supplementary Materials.

## References

[1] Arduengo A.J., Harlow R.L., Kline M. (1991). A Stable Crystalline Carbene. J. Am. Chem. Soc..

[2] Cheng J., Wang L., Wang P., Deng L. (2018). High-Oxidation-State 3d Metal (Ti–Cu) Complexes with N-Heterocyclic Carbene Ligation. Chem. Rev..

[3] Arduengo A.J. (1999). Looking for Stable Carbenes: The Difficulty in Starting Anew. Acc. Chem. Res..

[4] Danopoulos A.A., Simler T., Braunstein P. (2019). N-Heterocyclic Carbene Complexes of Copper, Nickel, and Cobalt. Chem. Rev..

[5] Shee S., Shree Ranganathappa S., Gadhave M.S., Gogoi R., Biju A.T. (2023). Enantioselective Synthesis of C–O Axially Chiral Diaryl Ethers by NHC-Catalyzed Atroposelective Desymmetrization. Angew. Chem. Int. Ed..

[6] Jia J., Luo J., Li W., Cui F., Pan Y., Tang H. (2024). Copper-Metallized Porous N-Heterocyclic Carbene Ligand Polymer-Catalyzed Regio-and Stereoselective 1, 2-Carboboration of Alkynes. Adv. Sci..

[7] Fritsch L., Vukadinovic Y., Lang M., Naumann R., Bertrams M., Kruse A., Schoch R., Müller P., Neuba A., Dierks P. (2024). Chemical and Photophysical Properties of Amine Functionalized bis-NHC-pyridine-RuII Complexes. ChemPhotoChem.

[8] Jiang Y., Fei H. (2023). N-Heterocyclic Carbene-Ligated Metal Complexes and Clusters for Photocatalytic CO_2_ Reduction. Inorg. Chem. Front..

[9] Huynh H.V. (2018). Electronic Properties of N-Heterocyclic Carbenes and Their Experimental Determination. Chem. Rev..

[10] Carroll X.B., Errulat D., Murugesu M., Jenkins D.M. (2022). Late Lanthanide Macrocyclic Tetra-NHC Complexes. Inorg. Chem..

[11] Henrion M., Ritleng V., Chetcuti M.J. (2015). Nickel N-Heterocyclic Carbene-Catalyzed C–C Bond Formation: Reactions and Mechanistic Aspects. ACS Catal..

[12] Jiang Y., Gendy C., Roesler R. (2018). Nickel, Ruthenium, and Rhodium NCN-Pincer Complexes Featuring a Six-Membered N-Heterocyclic Carbene Central Moiety and Pyridyl Pendant Arms. Organometallics.

[13] Weiss D.T., Anneser M.R., Haslinger S., Pöthig A., Cokoja M., Basset J.-M., Kühn F.E. (2015). NHC Versus Pyridine: How “Teeth” Change the Redox Behavior of Iron (II) Complexes. Organometallics.

[14] Masaeli S.E., Teimouri M., Adhikari B., Attarroshan M., Akin J.W., Raju S., Stokes S.L., Emerson J.P. (2023). Sodium Trifluoroacetate Mediated Copper-Catalyzed Aza-Michael Addition of α, β-Unsaturated Olefins with Aromatic Amines. Tetrahedron Lett..

[15] Raju S., Teimouri M., Adhikari B., Donnadieu B., Stokes S.L., Emerson J.P. (2023). Copper Complexes for the Chemoselective N-Arylation of Arylamines and Sulfanilamides via Chan–Evans–Lam Cross-Coupling. Dalton Trans..

[16] Hu K., Gao Y., Jin J. (2022). Nickel-Catalyzed N-Arylation of Diarylamines for Triarylamine Synthesis. Organometallics.

[17] Jacob N., Zaid Y., Oliveira J.C., Ackermann L., Wencel-Delord J. (2022). Cobalt-Catalyzed Enantioselective C–H Arylation of Indoles. J. Am. Chem. Soc..

[18] Guérin V., Legault C.Y. (2021). Synthesis of NHC-Iridium (III) Complexes Based on N-Iminoimidazolium Ylides and Their Use for the Amine Alkylation by Borrowing Hydrogen Catalysis. Organometallics.

[19] Li J., He D., Lin Z., Wu W., Jiang H. (2021). Recent Advances in NHC–Palladium Catalysis for Alkyne Chemistry: Versatile Synthesis and Applications. Org. Chem. Front..

[20] Minnick J.L., Raincrow J., Meinders G., Latifi R., Tahsini L. (2023). Synthesis, Characterization, and Spectroscopic Studies of 2,6-Dime-thylpyridyl-Linked Cu(I)–CNC Complexes: The Electronic Influence of Aryl Wingtips on Copper Centers. Inorg. Chem..

[21] Siddique M., Boity B., Rit A. (2023). Heteroditopic Chelating NHC Ligand-Supported CoIII Complexes: Catalysts for the Reductive Functionalization of Carbon Dioxide under Ambient Conditions. Organometallics.

[22] Zheng D.-Z., Xiong H.-G., Song A.-X., Yao H.-G., Xu C. (2022). Buchwald–Hartwig Amination of Aryl Esters and Chlorides Catalyzed by the Dianisole-Decorated Pd–NHC Complex. Org. Biomol. Chem..

[23] Díez-González S., Escudero-Adán E.C., Benet-Buchholz J., Stevens E.D., Slawin A.M., Nolan S.P. (2010). [(NHC) CuX] Complexes: Synthesis, Characterization and Catalytic Activities in Reduction Reactions and Click Chemistry. On the Advantage of Using Well-Defined Catalytic Systems. Dalton Trans..

[24] Tulloch A.A., Danopoulos A.A., Kleinhenz S., Light M.E., Hursthouse M.B., Eastham G. (2001). Structural Diversity in Pyridine-N-Functionalized Carbene Copper (I) Complexes. Organometallics.

[25] Cope J.D., Sheridan P.E., Galloway C.J., Awoyemi R.F., Stokes S.L., Emerson J.P. (2020). Synthesis and Characterization of a Tetradentate, N-Heterocyclic Carbene Copper (II) Complex and Its Use as a Chan–Evans–Lam Coupling Catalyst. Organometallics.

[26] Sharma M., Adhikari B., Awoyemi R.F., Perkins A.M., Duckworth A.K., Donnadieu B., Wipf D.O., Stokes S.L., Emerson J.P. (2022). Copper (II) NHC Catalyst for the Formation of Phenol from Arylboronic Acid. Chemistry.

[27] Adhikari B., Teimouri M., Akin J.W., Raju S., Stokes S.L., Emerson J.P. (2023). Cu−NHC Complex for Chan-Evans-Lam Cross-Coupling Reactions of N-Heterocyclic Compounds and Arylboronic Acids. Eur. J. Org. Chem..

[28] Simonovic S., Whitwood A.C., Clegg W., Harrington R.W., Hursthouse M.B., Male L., Douthwaite R.E. (2009). Synthesis of Copper(I) Complexes of N-Heterocyclic Carbene–Phenoxyimine/Amine Ligands: Structures of Mononuclear Copper(II), Mixed-Valence Copper(I)/(II), and Copper(II) Cluster Complexes.

[29] Meng G., Kakalis L., Nolan S.P., Szostak M. (2019). A Simple 1H NMR Method for Determining the σ-Donor Properties of N-Heterocyclic Carbenes. Tetrahedron Lett..

[30] Sun H., Chen S., Zhong A., Sun R., Jin J., Yang J., Liu D., Niu J., Lu S. (2023). Tuning Photophysical Properties via Positional Isomerization of the Pyridine Ring in Donor–Acceptor-Structured Aggregation-Induced Emission Luminogens Based on Phenylmethylene Pyridineacetonitrile Derivatives. Molecules.

[31] Schwartz T.M., Burnett M.E., Green K.N. (2020). Electronic Influence of Substitution on the Pyridine Ring within NNN Pincer-Type Molecules. Dalton Trans..

[32] Sciortino G., Maréchal J.-D., Fábián I., Lihi N., Garribba E. (2020). Quantitative Prediction of Electronic Absorption Spectra of Copper(II)–Bioligand Systems: Validation and Applications. J. Inorg. Biochem..

[33] Chiyindiko E., Langner E.H.G., Conradie J. (2022). Spectroscopic Behaviour of Copper(II) Complexes Containing 2-Hydroxyphenones. Molecules.

[34] Wang J., Ren J., Tang Q., Wang X., Wang Y., Wang Y., Du Z., Wang W., Huang L., Belfiore L.A. (2022). An Efficient Cyan Emission from Copper (II) Complexes with Mixed Organic Conjugate Ligands. Materials.

[35] Astakhov A.V., Khazipov O.V., Chernenko A.Y., Pasyukov D.V., Kashin A.S., Gordeev E.G., Khrustalev V.N., Chernyshev V.M., Ananikov V.P. (2017). A New Mode of Operation of Pd-NHC Systems Studied in a Catalytic Mizoroki–Heck Reaction. Organometallics.

[36] Chernyshev V.M., Denisova E.A., Eremin D.B., Ananikov V.P. (2020). The Key Role of R–NHC Coupling (R = C, H, Heteroatom) and M–NHC Bond Cleavage in the Evolution of M/NHC Complexes and Formation of Catalytically Active Species. Chem. Sci..

[37] Comba P., Morgen M., Wadepohl H. (2013). Tuning of the Properties of Transition-Metal Bispidine Complexes by Variation of the Basicity of the Aromatic Donor Groups. Inorg. Chem..

[38] Saint Program Included in the Package Software: APEX4 v2022.10.0. https://www.brukersupport.com/ProductDetail/9269.

[39] Blessing R.H. (1995). An empirical correction for absorption anisotropy. Acta Crystallogr. Sect. A Found. Crystallogr..

[40] Sheldrick G.M. (2005). SHELXT–Integrated space-group and crystal-structure determination. Acta Crystallogr. Sect. A Found. Adv..

[41] Sheldrick G.M. (2015). Crystal structure refinement with SHELXL. Acta Crystallogr. Sect. C Struct. Chem..

[42] APEX4 v2022.10.0, AXS Bruker Program. https://www.brukersupport.com/ProductDetail/9269.

