# Correction: Adhikari et al. Synthesis and Characterization of Symmetrical *N*-Heterocyclic Carbene Copper(II) Complexes—An Investigation of the Influence of Pyridinyl Substituents. *Molecules* 2024, *29*, 3542

**DOI:** 10.3390/molecules31010135

**Published:** 2025-12-31

**Authors:** Bhupendra Adhikari, Selvam Raju, Raymond Femi Awoyemi, Bruno Donnadieu, David O. Wipf, Sean L. Stokes, Joseph P. Emerson

**Affiliations:** Department of Chemistry, Mississippi State University, Starkville, MS 39762, USA

## Text Correction

There was an error in the original publication [1]. Figure 2 citation should be changed to Figure 3 in Section 2 Paragraph 5. This is referring to a long bond, which is related to Figure 3 not Figure 2. 

## Reference

In the original publication, reference 20 was mistakenly assigned as follows:

20. Yan, J.; Wang, Y.-B.; Zhu, Z.-H.; Li, Y.; Zhu, X.; Hao, X.-Q.; Song, M.-P. Synthesis, Characterization, and Catalytic Studies of Unsymmetrical Chiral NCC Pincer Pd(II) and Ni(II) Complexes Bearing (Imidazolinyl) Aryl NHC Ligands. *Organometallics* **2018**, *37*, 2325–2334.

However, this citation has been correctly attributed as follows:

20. Minnick, J.L.; Raincrow, J.; Meinders, G.; Latifi, R.; Tahsini, L. Synthesis, Characterization, and Spectroscopic Studies of 2,6-Dime-thylpyridyl-Linked Cu(I)–CNC Complexes: The Electronic Influence of Aryl Wingtips on Copper Centers. *Inorg. Chem.* **2023**, *62*, 15912–15926. https://doi.org/10.1021/acs.inorgchem.3c01973.

The correct citation has now been corrected in the Reference section of the manuscript. The authors state that the scientific conclusions are unaffected. This correction was approved by the Academic Editor. The original publication has also been updated.
