# Supplementary material for: Synthesis and Characterization of Symmetrical N-Heterocyclic Carbene Copper(II) Complexes—An Investigation of the Influence of Pyridinyl Substituents
Source: Molecules. 2024 Jul 27;29(15):3542. doi: 10.3390/molecules29153542 (PMC11314359; doi:10.3390/molecules29153542)
Supplement: Supplementary file 1 [file molecules-29-03542-s001.zip › molecules-3081684-supplementary finalformat_updated.pdf]

Supplementary Materials

Synthesis and Characterization of Symmetrical *N*-Heterocyclic Carbene Copper(II) Complexes—An Investigation of the Influence of Pyridinyl Substituents

Bhupendra Adhikari, Selvam Raju, Raymond Femi Awoyemi, Bruno Donnadieu, David O. Wipf, Sean L. Stokes * and Joseph P. Emerson *

Table of Contents

1.	General information	2-3
2.	General synthetic procedures	4-5
3.	Spectroscopic and physical data of all synthesized compounds	6-12
4.	NMR Spectra (Figure S1-S42)	13-33
5.	HRMS-ESI Spectrum (Figure S43-S45)	34-35
6.	Single Crystal XRD (Figure S46-S48 and Tables S1-S3)	36-41
7.	CV data (Figure S49-S52)	42-43
8.	FT-IR data (Figure S53-S56)	44-45
9.	EA data (Table S4)	46
10.	PXRD (Figure S57-S60)	46-48
11.	References	49

General Information

Benzimidazole, picolinic acid, picolinic esters, picolyl chloride (TCI), ammonium hexafluorophosphate, copper (II) acetate (Alfa Aesar), and potassium carbonate were used as received. All reagents used for this work were of analytical grade and used as received. The solvents used were of HPLC grade and were obtained from Fisher Scientific (New Jersey, USA). All the products and ligand species were characterized by melting points (m.p), ^{1}H-NMR, ^{13}C-NMR, mass spectra and infrared spectra (IR). Melting points were measured on an Electrothermal MEL-TEMP melting point apparatus; IR spectra were recorded on a ThermoScientific iS5 spectrometer; ^{1}H-NMR and ^{13}C-NMR spectra were obtained on Bruker 500 MHz NMR and chemical shifts were reported in parts per million (ppm, δ) with $CHCl_3$ as a reference. Proton coupling patterns are described as singlet (s), doublet (d), triplet (t), triplet of doublet (td), doublet of doublet (dd), multiplet (m); Coupling constants (J) are quoted in Hz. Carbon-13 nuclear magnetic resonance (^{13}C- NMR) data were acquired at 125 MHz. High resolution mass spectrometry was carried out on a Bruker micrOTOF QII HRMS using an ESI source in positive mode. The water used was initially deionized using a reverse osmosis system. All the used as purchased chemicals were used without further purification. All the salt products were purified using flash column chromatography using SiliaFlash® P60 (230-400 mesh) silica gels. All the heating reactions were carried out using an oil bath equipped with a digital temperature controller.

The cyclic voltammetry data were acquired using an EmStat3 potentiostat (PalmSens Compact Electrochemical Interfaces) in a three-electrode cell with a glassy carbon electrode (GCE), graphite rod, and Ag wire quasi-reference electrode (Ag QRE) as the working, auxiliary, and reference electrodes, respectively. 1 mM of the analyte was dissolved in the electrolyte solution containing 100 mM of tetrabutylammonium hexafluorophosphate (VI) (n-Bu_4PF_6) used as the supporting electrolyte. Ferrocene was added at the end of the experiment as a reference potential standard. The potential was scanned between -1.0 V to 1.5 V vs. the Ag QRE in acetonitrile, starting from the open circuit potential OCP. The acetonitrile was purified prior to use. The electrolyte solution was purged with ultra-high purity (UHP) Ar for at least 30 min before the electrochemical measurements, and Ar was passed over the solution during CV collection. All data were acquired at room temperature.

A blue prism like single crystal of Cu^{2+}bPymBI-Cl, Cu^{2+}bPymBI-Me and Cu^{2+}bPymBI-OMe was selected for the X-ray crystallographic analysis and mounted on a cryoloop using an oil cryoprotectant. The X-ray intensity data was measured at low temperature (T = 100K), using a three circles goniometer geometry with a fixed Chie angle at = 54.74 deg Bruker AXS D8 Venture, equipped with a Photon 100 CMOS active pixel sensor detector. A monochromatized Copper X-ray radiation (λ = 1.54178 Å) was selected for the measurement. The frames were integrated with the Bruker SAINT software package 1 using a narrow-frame algorithm.

Procedure for the synthesis of methyl picolinate derivatives

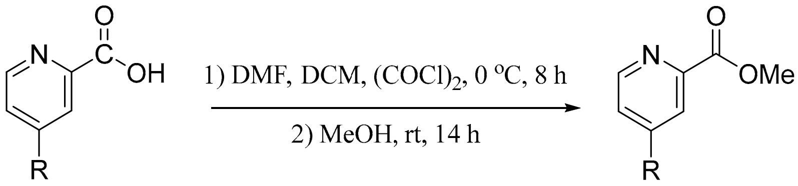

Picolinic acid derivative 1.0 equivalent (12.7 mmol), oxalyl chloride 3.5 equivalent (38.1 mmol), and a catalytic amount (5 drops) of DMF were combined with 50 mL of DCM in a round bottom flask at 0 °C and stirred for 8 hours under N_2 atmosphere. The solvent was then removed using rotary evaporation at reduced pressure. The residue was treated with 20 mL of MeOH and stirred at RT for 14 hours to achieve conversion of the respective esters.

Procedure for the synthesis of pyridin-2-ylmethanol derivatives

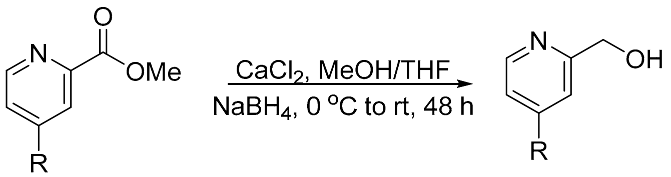

1.0 equivalent (8.8 mmol) of esters are reduced to alcohols by loading 5.0 equivalent (43.8 mmol) of $CaCl_2$ in 100 ml MeOH:THF (6:3.5) solvent at 0 °C to RT, followed by the addition of 3.0 equivalent (26.3 mmol) of $NaBH_4$ in three installments. The same addition was performed after 24 hours maintaining 0 °C to RT for more 24 hours to obtain the alcohol.

Procedure for the synthesis of 2-(chloromethyl)pyridine derivatives

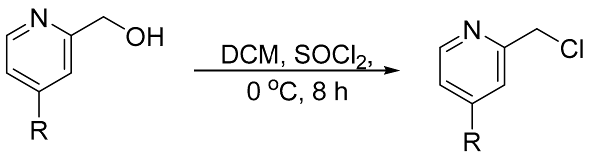

Pyridin-2-ylmethanol derivative 1 equivalent (6.9 mmol), and thionyl chloride 2.5 equivalent (17.5 mmol), with 50 mL of DCM in a round bottom flask at 0 °C and stirred for 8 hours under N_2 atmosphere. The solvent was then removed using rotary evaporation at reduced pressure. The residue was treated with *n*-hexane to get the precipitate/sticky liquid.

Procedure for the synthesis of NHC precursor ligand

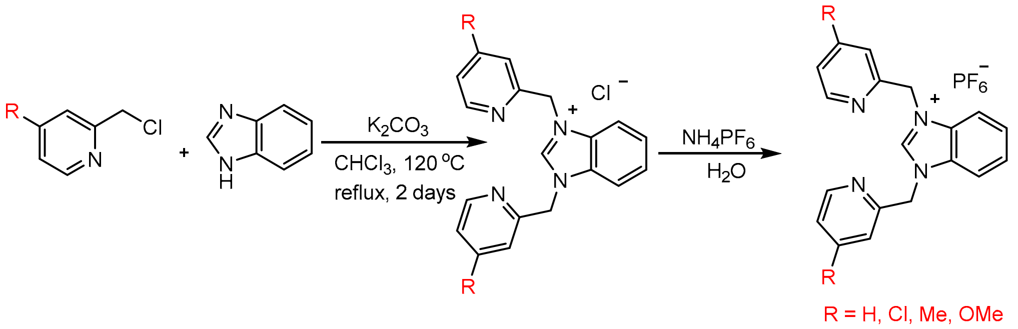

The mixture of 2.0 equivalent (3.4 mmol) of 2-picolyl chloride derivative, 1.0 equivalent (1.7 mmol) of benzimidazole, and 5.0 equivalent (8.5 mmol) of potassium carbonate were mixed $CHCl_3$ solvent in a pressure tube and refluxed for 48 h at 120 ºC. The compound was filtered and the filtrate containing solvent was removed completely under reduced pressure by rotary vaporization. The residue after rotary vaporization was again dissolved in DCM and dried over $MgSO_4$ and the saturated complex was treated with *n*-hexane to obtain brown solid. The chloride salt of the ligand (1 equivalent, 0.5mmol) was dissolved in the minimum amount of water followed by the addition of 5.0 equivalent (2.5mmol) of ammonium hexafluorophosphate to obtain brown colored complex and dried under vacuum.

Procedure for the synthesis of Cu(NHC) complex

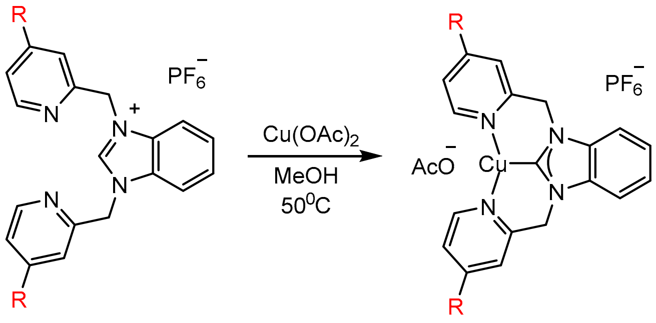

1.0 equivalent of hexafluorophosphate version of ligand was mixed with 1.0 equivalent of $Cu(OAc)_2$ in minimum MeOH (5.0 mL) in a flask and stirred for 2 h at 50 °C. A blue-colored precipitation was obtained and was separated by filtration followed by washing with the MeOH.

Spectroscopic and physical data of all synthesized compounds

1,3-bis(pyridin-2-ylmethyl)-1*H*-benzo[d]imidazol-3-ium chloride (1d)

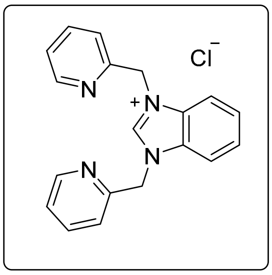

Yield 40% (228 mg), Brown solid, m.p: 112-114 °C; R*f*: 0.51 (DCM:EA:MeOH; 7:3:0.5); ^{1}H NMR (500 MHz, DMSO-d$_6$) *δ*: 10.22 (s, 1H), 8.50 (d, *J* = 5.0 Hz, 2H), 7.97–7.86 (m, 2H), 7.92–7.91 (m, 2H), 7.69 (d, *J* = 10.0 Hz, 2H), 7.63–7.61 (m,2H), 7.40–7.38 (m,2H), 3.35 (s, 4H), ppm; ^{13}C NMR (125 MHz, DMSO-d$_6$) *δ*: 153.5, 150.1, 144.5, 138.1, 131.7, 127.2, 124.2, 123.2, 114.5, 51.4, ppm; FT-IR (KBr) ṽ (cm^{-1}) 1580, 1510, 1430, 1375, 1280.

1,3-bis(pyridin-2-ylmethyl)-1*H*-benzo[d]imidazol-3-ium chloride (1e)

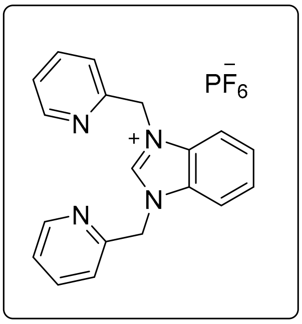

Yield 95% (211 mg), Brown solid, m.p: 168-170 °C; R*f*: 0.51 (DCM:EA:MeOH; 7:3:0.5); ^{1}H NMR (500 MHz, DMSO-d$_6$) *δ*: 10.12 (s, 1H), 8.51 (d, *J* = 5.0 Hz, 2H), 7.97–7.95 (m, 2H), 7.94–7.91 (m, 2H), 7.67 (d, *J* = 5.0 Hz, 2H), 7.64 – 7.63 (m, 2H), 7.41–7.38 (t, J = 10.0 Hz, 2H), 6.00 (s, 4H), ppm; ^{13}C NMR (125 MHz, DMSO-d$_6$) *δ*: 153.5, 150.1, 144.5, 138.1, 131.7, 127.2, 124.2, 123.2, 114.5, 51.4, ppm. ^{19}F NMR (470 MHz, DMSO-d$_6$) *δ*: -70.11 (d, *J* = 710.3 Hz) ppm; ^{31}P NMR (202 MHz, DMSO-d$_6$) *δ*: -144.16 (septet, *J* = 712.5 Hz) ppm; FT-IR (KBr) ṽ (cm^{-1}) 1580, 1510, 1430, 1375, 1280.

Methyl 4-methylpicolinate (2a)

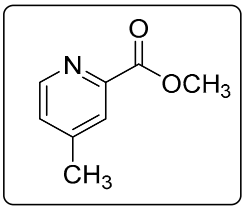

Yield 76% (1.46 g), Brown liquid, ^{1}H NMR (500 MHz, $CDCl_3$) δ: 8.60 (d, J = 5.0 Hz 1H), 7.97 (s, 1H), 7.31 (d, J = 5.0 Hz, 1H), 4.00 (s, 3H), 2.44 (s, 3H) ppm; ^{13}C NMR (125 MHz, $CDCl_3$) δ: 166.8, 149.5, 148.4, 147.6, 127.7,125.9, 52.7, 20.9 ppm.

(4-methylpyridin-2-yl)methanol (2b)

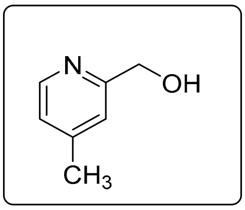

Yield 85% (917 mg), Brown liquid, ^{1}H NMR (500 MHz, $CDCl_3$) δ: 8.31 (s, 1H), 7.18 (s, 1H), 6.97 (s, 1H), 4.71 (s, 2H) 2.31 (s, 3H) ppm; ^{13}C NMR (125 MHz, $CDCl_3$) δ: 159.9, 148.2, 148.0, 123.4, 121.9, 64.2, 21.0 ppm.

2-(chloromethyl)-4-methylpyridine (2c)

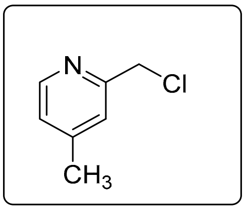

Yield 95% (937 mg), Red sticky liquid, ^{1}H NMR (500 MHz, $CDCl_3$) δ: 8.42 (d, J = 5.0 Hz 1H), 7.28 (s, 1H), 7.04 (d, J = 5.0 Hz, 1H), 4.63 (s, 2H), 2.35 (s, 3H) ppm; ^{13}C NMR (125 MHz, $CDCl_3$) δ: 156.3, 149.2, 148.4, 124.0, 123.7, 46.7, 21.0 ppm.

1,3-bis((4-methylpyridin-2-yl)methyl)-1*H*-benzo[*d*]imidazol-3-ium chloride (2d)

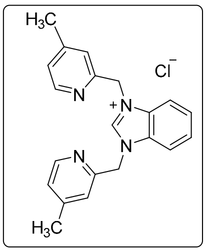

Yield 41% (240 mg), Faded brown solid, m.p: 201-203 ºC; R*f*: 0.08 (DCM:EA:MeOH; 7:3:0.5); ^{1}H NMR (500 MHz, $CDCl_3$) *δ*: 11.66 (s, 1H), 8.25 (s, 2H), 7.7 (s, 2H), 7.53 (d, *J* = 5.0 Hz, 4H), 6.97(s, 2H), 5.91 (s, 4H) 2.25 (s, 6H) ppm; ^{13}C NMR (125 MHz, $CDCl_3$) *δ*: 152.4, 149.3, 149.1, 143.9, 131.9, 126.7, 124.8, 124.3, 114.2, 52.6, 20.9 ppm. FT-IR (KBr) ṽ (cm^{-1}) 1580, 1510, 1430, 1375, 1280; ; HRMS (ESI^{+}), calcd for $C_{21}H_{21}N_4$ $[M–Cl]^{+}$ 329.1761 found 329.1751.

1,3-bis((4-methylpyridin-2-yl)methyl)-1*H*-benzo[*d*]imidazol-3-ium hexafluorophosphate (2e)

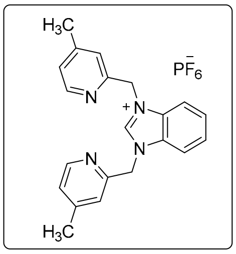

Yield 97% (230 mg), brown solid, m.p: 153-155 ºC; R*f*: 0.46 (DCM:EA:MeOH; 7:3:0.5); ^{1}H NMR (500 MHz, $CDCl_3$) *δ*: 9.48 (s, 1H), 8.36 (d, *J* = 5, 2H), 7.83 (s, 2H), 7.55 (d, *J* = 5.0 Hz, 2H), 7.44 (s, 2H), 7.08 (d, *J* = 10, 2H), 5.68 (s, 4H), 2.36 (s, 6H) ppm; ^{13}C NMR (125 MHz, $CDCl_3$) *δ*: 151.9, 149.6, 149.5, 142.1, 131.7, 127.3, 125.1, 124.4, 114.1, 52.8, 20.9 ppm. ^{19}F NMR (470 MHz, DMSO-d_6) *δ*: -70.13 (d, *J* = 710.28 Hz) ppm; ^{31}P NMR (202 MHz, DMSO-d_6) *δ*: -144.19 (septet, *J* = 712.46 Hz) ppm; FT-IR (KBr) ṽ (cm^{-1}) 1580, 1510, 1430, 1375, 1280; HRMS (ESI^{+}), calcd for $C_{21}H_{21}N_4$ $[M–PF_6]^{+}$ 329.1761 found 329.1751.

methyl 4-methoxypicolinate (3a)

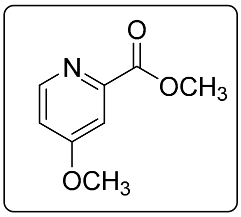

Yield 70% (1.34 g), Brown liquid, ^{1}H NMR (500 MHz, $CDCl_3$) δ: 8.54 (d, J = 5.0 Hz 1H), 7.67 (s, 1H), 6.95 (d, J = 5.0 Hz, 1H), 4.01 (s, 3H), 3.92 (s, 3H) ppm; ^{13}C NMR (125 MHz, $CDCl_3$) δ: 166.6, 165.8, 150.9, 149.6, 113.1,111.20, 55.6, 52.9 ppm.

(4-methoxypyridin-2-yl)methanol (3b)

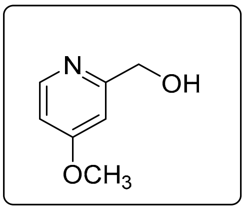

Yield 80% (975 mg), White solid, ^{1}H NMR (500 MHz, $CDCl_3$) δ: 8.28 (s, 1H), 6.89 (s, 1H), 6.69 (s, 1H), 4.70 (s, 2H) 3.82 (s, 3H) ppm; ^{13}C NMR (125 MHz, $CDCl_3$) δ: 166.5, 162.0, 149.5, 106.9, 106.01, 64.3, 55.2 ppm.

2-(chloromethyl)-4-methoxypyridine (3c)

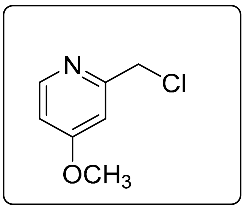

Yield 87% (958 mg), Red sticky liquid, ^{1}H NMR (500 MHz, $CDCl_3$) δ: 8.27 (d, J = 5.0 Hz 1H), 6.89 (s, 1H), 6.66 (d, J = 5.0 Hz, 1H), 4.52 (s, 2H), 3.75 (s, 3H) ppm; ^{13}C NMR (125 MHz, $CDCl_3$) δ: 156.3, 149.2, 148.4, 124.0, 123.7, 46.7, 21.0 ppm.

1,3-bis((4-methoxypyridin-2-yl)methyl)-1*H*-benzo[*d*]imidazol-3-ium chloride (3d)

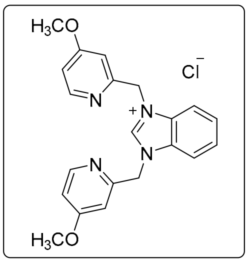

Yield 29% (200 mg), Brown solid, m.p: 169-171 ºC; R*f*: 0.09 (DCM:EA:MeOH; 7:3:0.5); ^{1}H NMR (500 MHz, $CDCl_3$) δ: 11.86 (s, 1H), 8.28 (d, J = 5.0 Hz, 2H), 7.91–7.89 (m, 2H), 7.54–7.52 (m, 4H), 6.75 (t, J = 5.0 Hz, 2H), 5.92 (s, 4H), 3.83 (s, 6H) ppm; ^{13}C NMR (125 MHz, $CDCl_3$) δ: 166.9, 154.2, 150.6, 144.0, 131.6, 126.9, 114.2, 110.9, 109.4, 55.9, 52.8 ppm. FT-IR (KBr) ṽ (cm^{-1}) 1580, 1510, 1430, 1375, 1280; HRMS (ESI^{+}), calcd for $C_{21}H_{21}N_4O_2$ $[M–Cl]^{+}$ 361.1659 found 361.1684.

1,3-bis((4-methoxypyridin-2-yl)methyl)-1*H*-benzo[*d*]imidazol-3-ium hexafluorophosphate (3e)

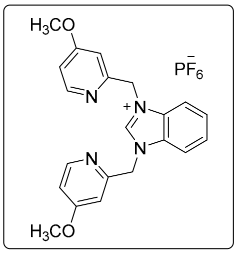

Yield 95% (240 mg), Brown solid, m.p: 168-170 ºC; R*f*: 0.51 (DCM:EA:MeOH; 7:3:0.5); ^{1}H NMR (500 MHz, DMSO-d_6) δ: 10.08 (s, 1H), 8.31 (d, J = 5.0 Hz, 2H), 7.96–7.95 (m, 2H), 7.64–7.62 (m, 2H), 7.30 (d, J = 5.0 Hz, 2H), 6.98 – 6.97 (m, 2H) 5.90 (s, 4H), 3.87 (s, 6H) ppm; ^{13}C NMR (125 MHz, DMSO-d_6) δ: 166.9, 154.9, 151.5, 144.4, 131.7, 127.2, 114.2, 110.0, 109.7, 96.1, 51.4 ppm. ^{19}F NMR (470 MHz, DMSO-d_6) δ: -70.14 (d, J = 710.28 Hz) ppm; ^{31}P NMR (202 MHz, DMSO-d_6) δ: -144.20 (septet, J = 711.04Hz) ppm; FT-IR (KBr) ṽ (cm^{-1}) 1580, 1510, 1430, 1375, 1280; HRMS (ESI^{+}), calcd for $C_{21}H_{21}N_4O_2$ $[M–PF_6]^{+}$ 361.1659 found 361.1684.

1-(4-chloropyridin-2-yl)ethan-1-one (4a)

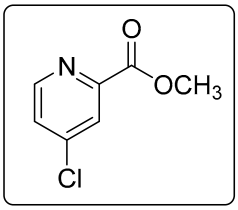

Yield 86% (1.86g), White solid, ^{1}H NMR (500 MHz, $CDCl_3$) δ: 8.67 (d, J = 5.0 Hz 1H), 8.13 (s, 1H), 7.52 (d, J = 5.0 Hz, 1H), 4.03 (s, 3H) ppm; ^{13}C NMR (125 MHz, $CDCl_3$) δ: 164.4, 150.5, 149.1, 145.2, 127.0, 125.5, 53.0 ppm.

(4-chloropyridin-2-yl)methanol (4b)

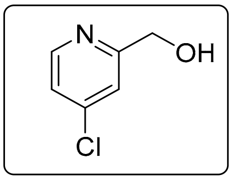

Yield 92% (1.15g), White solid, ^{1}H NMR (500 MHz, $CDCl_3$) δ: 8.33 (d, J = 5.0 Hz 1H), 7.47 (s, 1H), 7.16 (d, J = 5.0 Hz, 1H), 4.75 (s, 2H) ppm; ^{13}C NMR (125 MHz, $CDCl_3$) δ: 162.5, 149.2, 145.1, 122.5, 121.0, 63.9 ppm.

4-chloro-2-(chloromethyl)pyridine (4c)

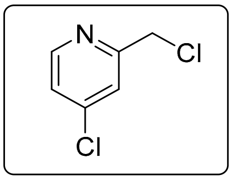

Yield 89% (1.12 g), red sticky liquid, ^{1}H NMR (500 MHz, $CDCl_3$) δ: 8.45 (d, J = 5.0 Hz 1H), 7.50 (s, 1H), 7.25–7.23 (m, 1H), 4.66 (s, 2H) ppm; ^{13}C NMR (125 MHz, $CDCl_3$) δ: 158.1, 150.2, 144.9, 123.3, 123.1, 45.9 ppm.

1,3-bis((4-chloropyridin-2-yl)methyl)-1*H*-benzo[*d*]imidazol-3-ium chloride (4d)

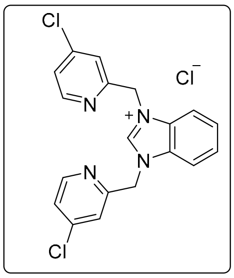

Yield 32% (220 mg), Brown solid, m.p: 140-142 ºC; R*f*: 0.25 (DCM:EA:MeOH; 7:3:0.5); ^{1}H NMR (500 MHz, DMSO-d$_6$) δ: 10.26 (s, 1H), 8.49 (d, J = 5.0 Hz, 2H), 8.01−7.99 (m, 2H), 7.91 (s, 2H), 7.66−7.64 (m, 2H), 7.57 (t, J = 5.0 Hz, 2H), 6.06 (s, 4H) ppm; ^{13}C NMR (125 MHz, DMSO-d$_6$) δ: 195.6, 151.5, 144.7, 144.3, 131.7, 127.3, 124.3, 123.4, 114.5, 50.9 ppm. FT-IR (KBr) ṽ (cm^{-1}) 1580, 1510, 1430, 1375, 1280; HRMS (ESI$^+$), calcd for $C_{19}H_{15}Cl_2N_4$ [M−Cl]$^+$ 369.0668 found 369.0684.

1,3-bis((4-chloropyridin-2-yl)methyl)-1*H*-benzo[*d*]imidazol-3-ium hexafluorophosphate (4e)

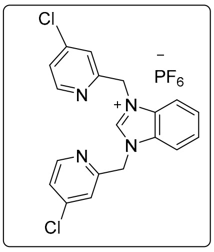

Yield 94% (242 mg), Brown solid, m.p: 138-140 ºC; R*f*: 0.67 (DCM:EA:MeOH; 7:3:0.5); ^{1}H NMR (500 MHz, DMSO-d$_6$) δ: 9.61 (s, 1H), 8.42 (s, 2H), 7.99 (s, 2H), 7.59 (s, 4H), 7.30 (s, 2H), 5.76 (s, 4H) ppm; ^{13}C NMR (125 MHz, DMSO-d$_6$) δ: 195.5, 151.5, 144.7, 144.4, 131.7, 127.4, 124.3, 123.4, 114.5, 50.9 ppm. ^{19}F NMR (470 MHz, DMSO-d$_6$) δ: -70.13 (d, J = 709.70 Hz) ppm; ^{31}P NMR (202 MHz, DMSO-d$_6$) δ: -144.19 (septet, J = 710.43 Hz) ppm; FT-IR (KBr) ṽ (cm^{-1}) 1580, 1510, 1430, 1375, 1280; HRMS (ESI$^+$), calcd for $C_{19}H_{15}Cl_2N_4$ [M−PF_6]$^+$ 369.0668 found 369.0684.

Figure S1. ^{1}H-NMR spectrum of compound **1d**, (500 MHz, DMSO-d_6)

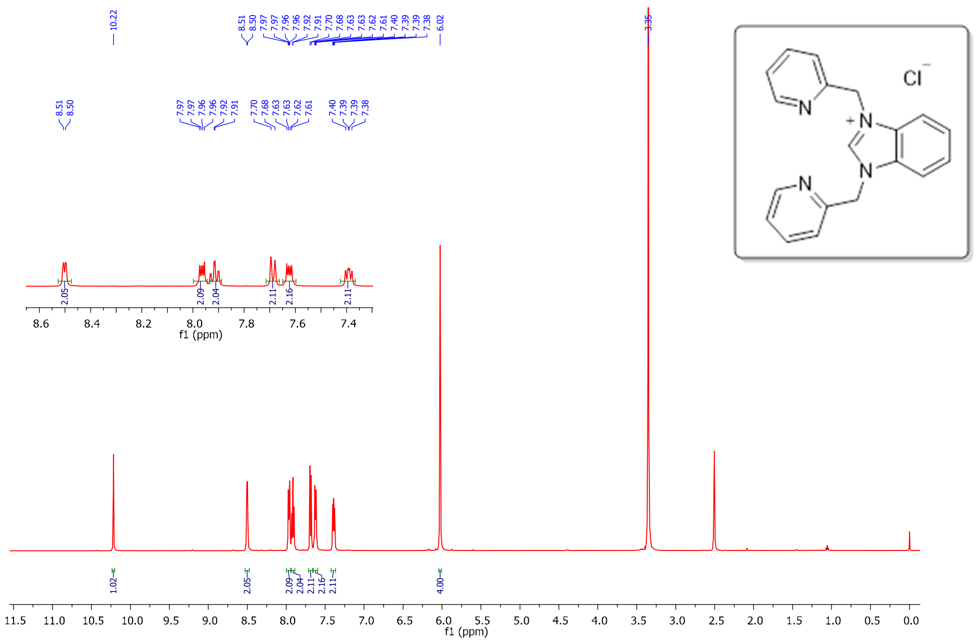

Figure S2. ^{13}C-NMR spectrum of compound **1d**, (125 MHz, DMSO-d_6)

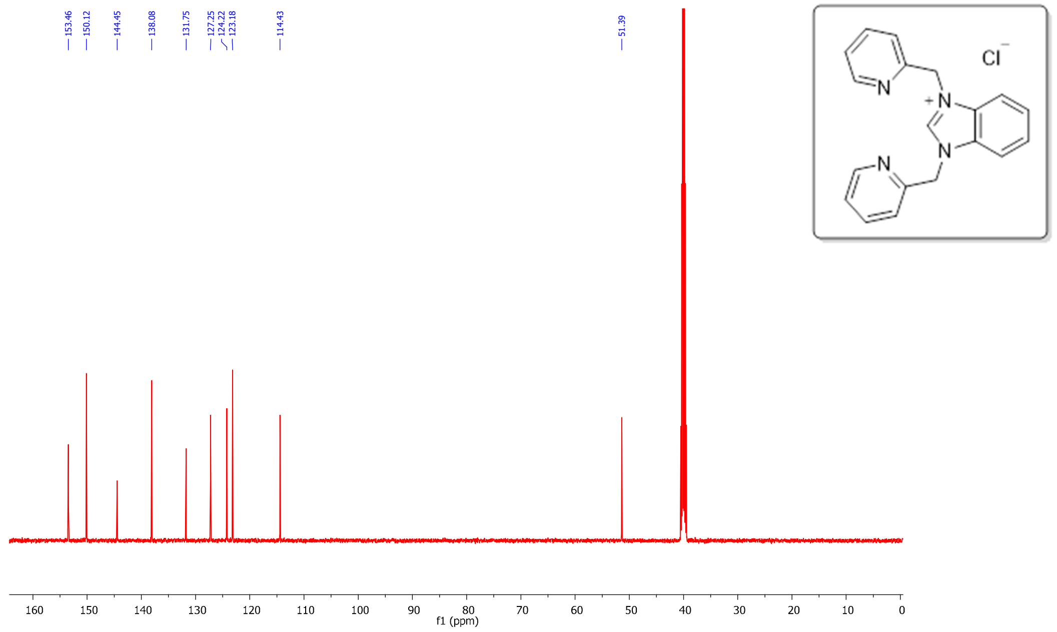

Figure S3. ^{1}H-NMR spectrum of compound **1e**, (500 MHz, DMSO-d$_6$)

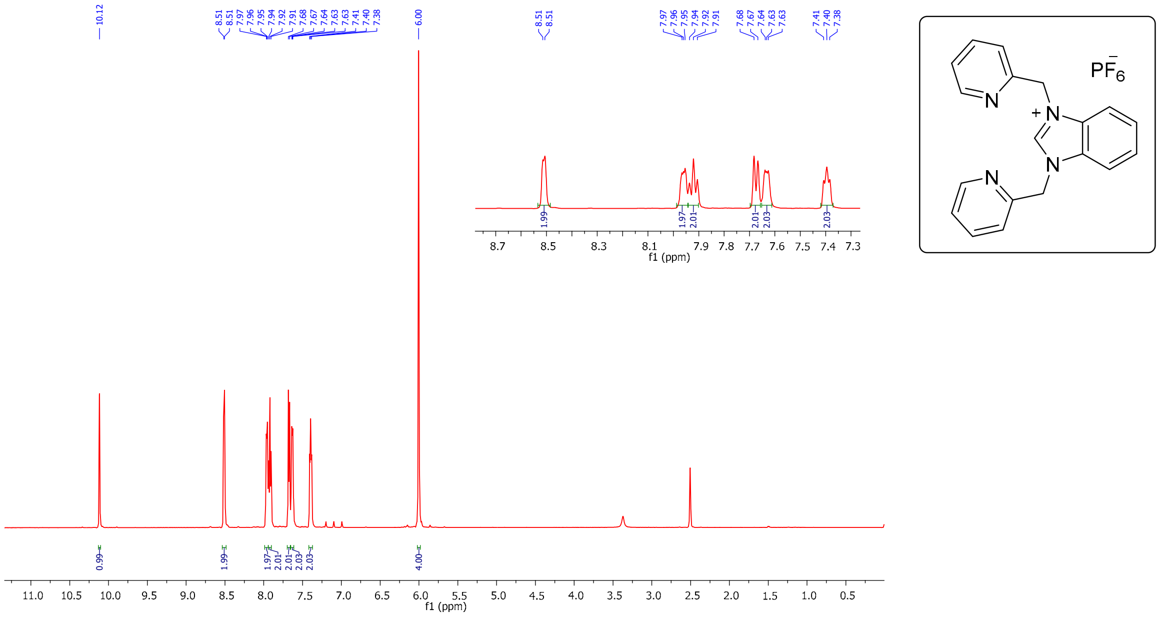

Figure S4. ^{13}C-NMR spectrum of compound **1e**, (125 MHz, DMSO-d$_6$)

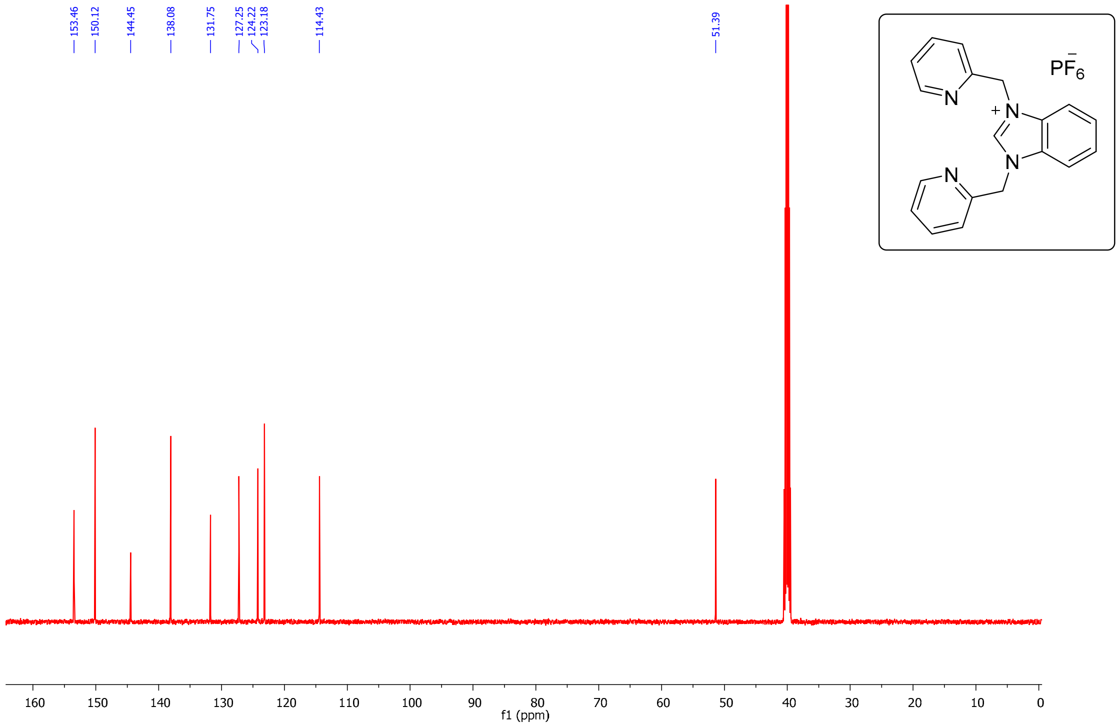

Figure S5. ^{19}F-NMR spectrum of compound **1e**, (470 MHz, DMSO-d$_6$)

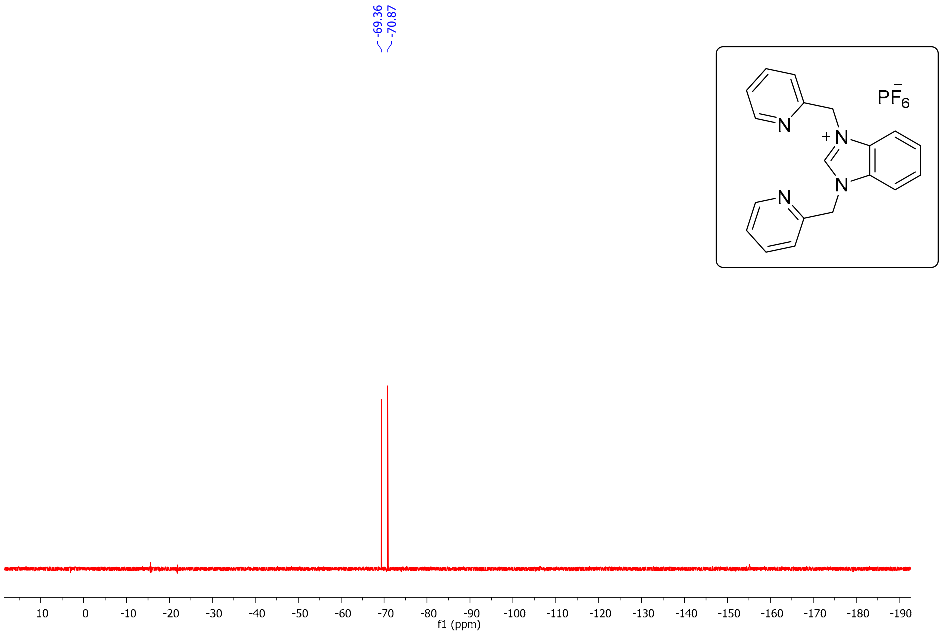

Figure S6. ^{31}P-NMR spectrum of compound **1e**, (202 MHz,DMSO-d$_6$)

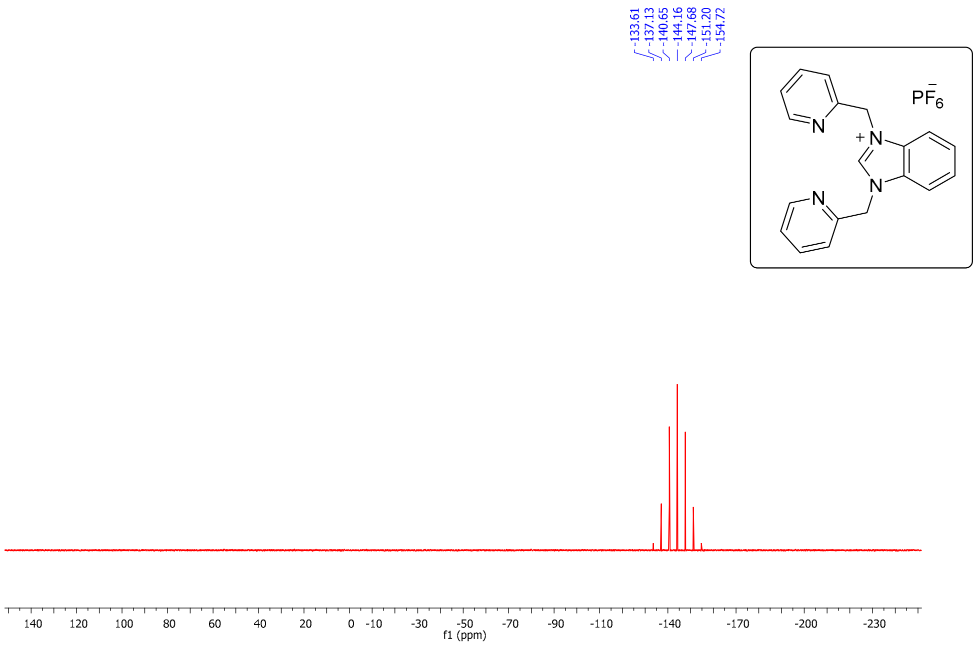

Figure S7. ^{1}H-NMR spectrum of compound **2a** (500 MHz, $CDCl_3$)

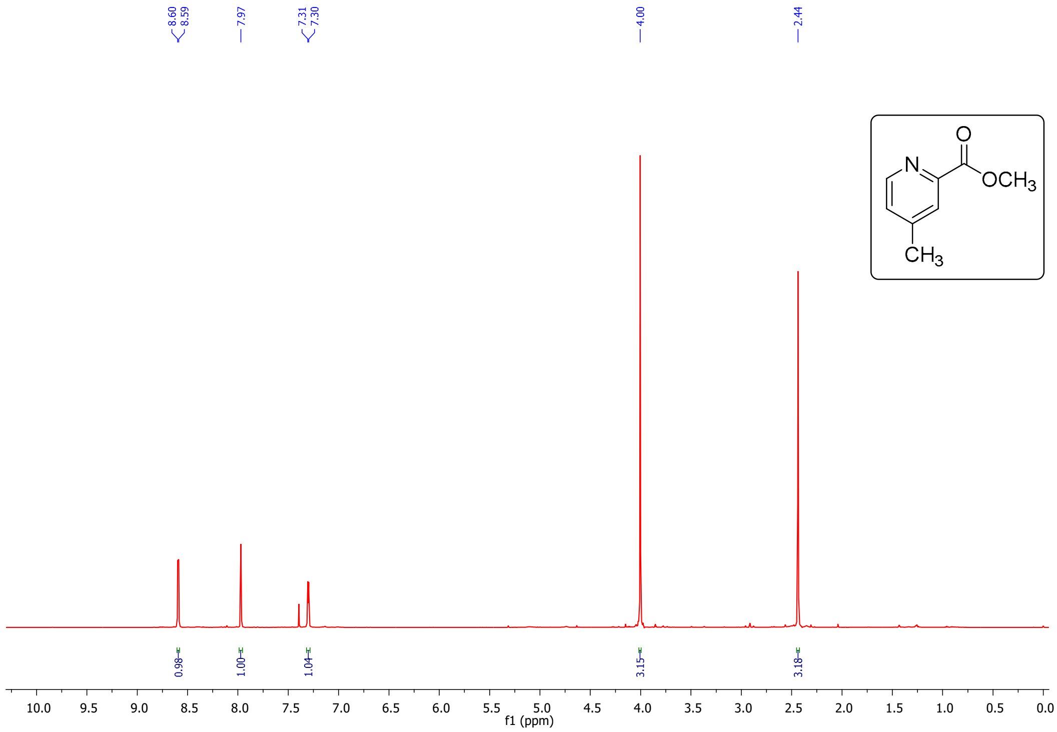

Figure S8. ^{13}C-NMR spectrum of compound **2a** (125 MHz, $CDCl_3$)

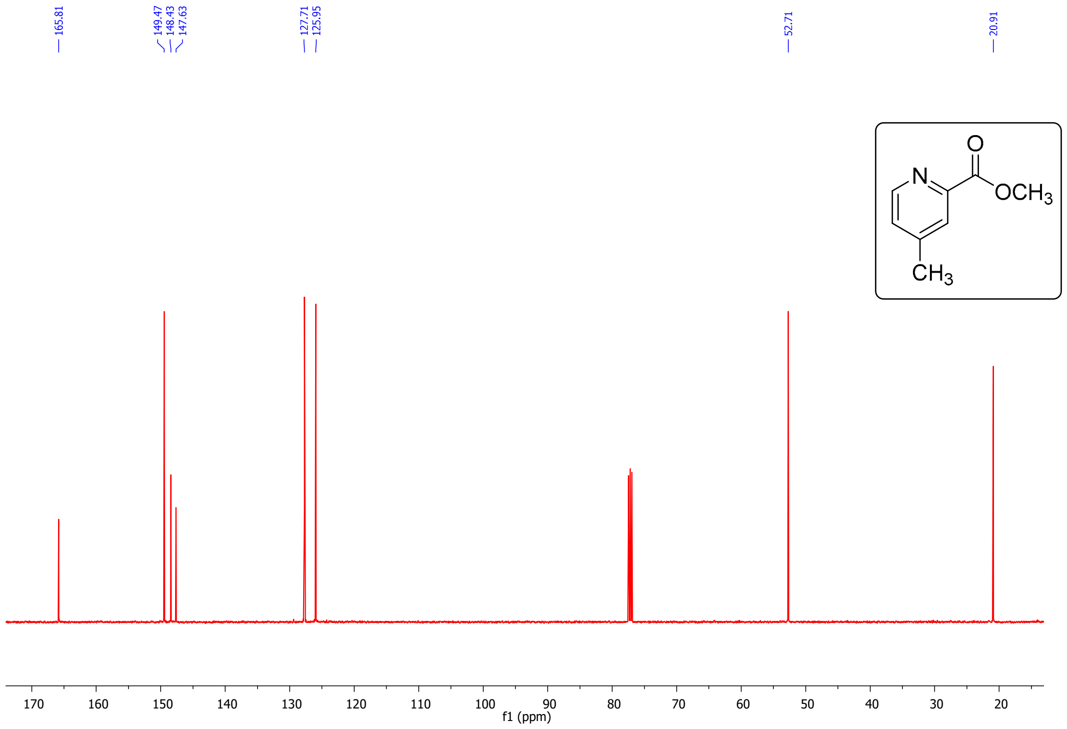

Figure S9. ^{1}H-NMR spectrum of compound **2b** (500 MHz, $CDCl_3$)

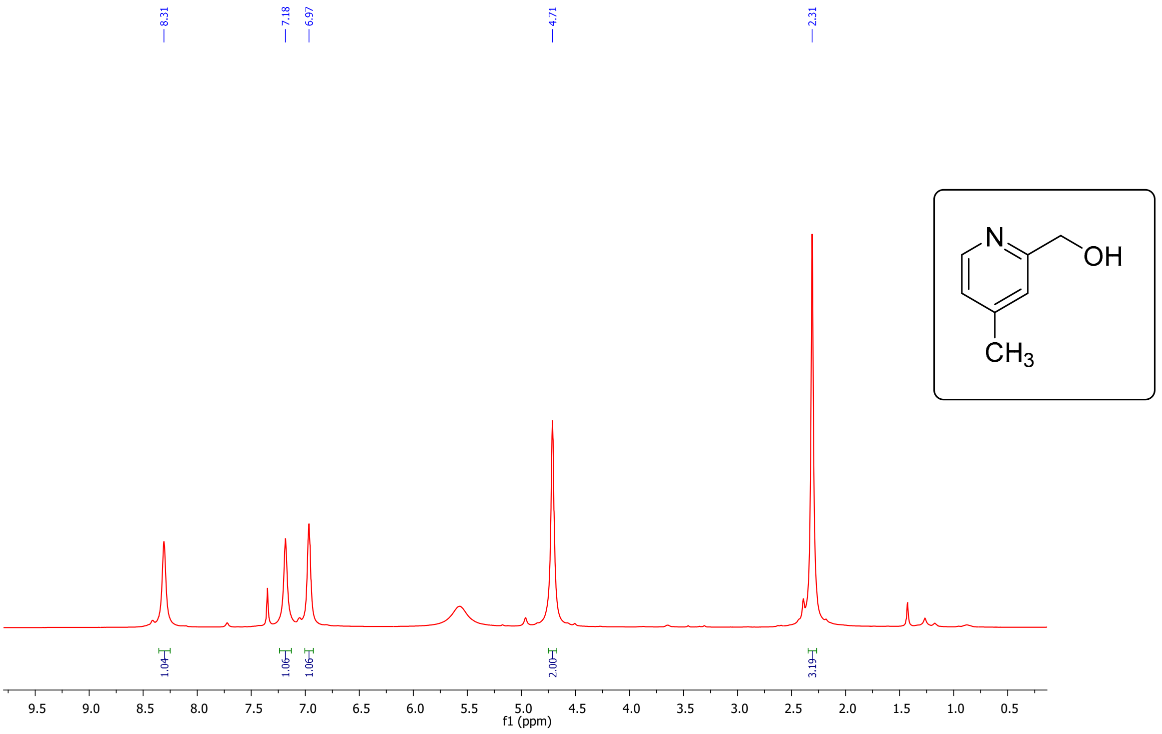

Figure S10. ^{13}C-NMR spectrum of compound **2b** (125 MHz, $CDCl_3$)

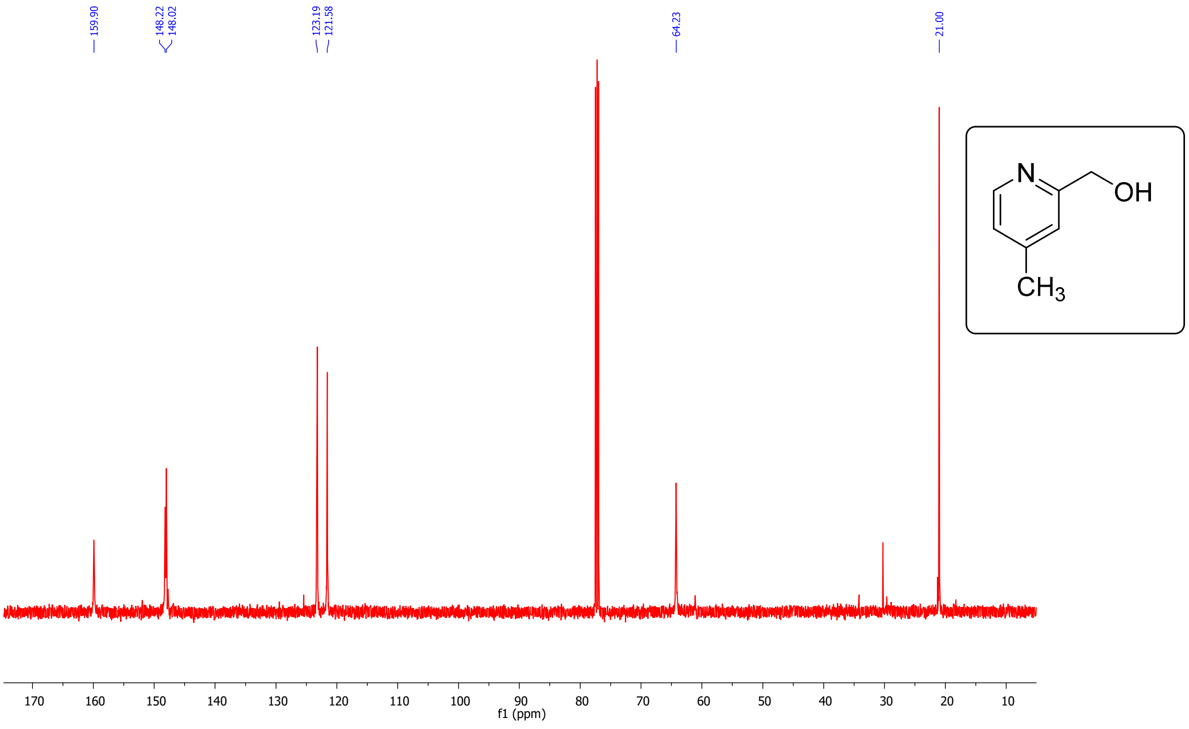

Figure S11. ^{1}H-NMR spectrum of compound **2c** (500 MHz, $CDCl_3$)

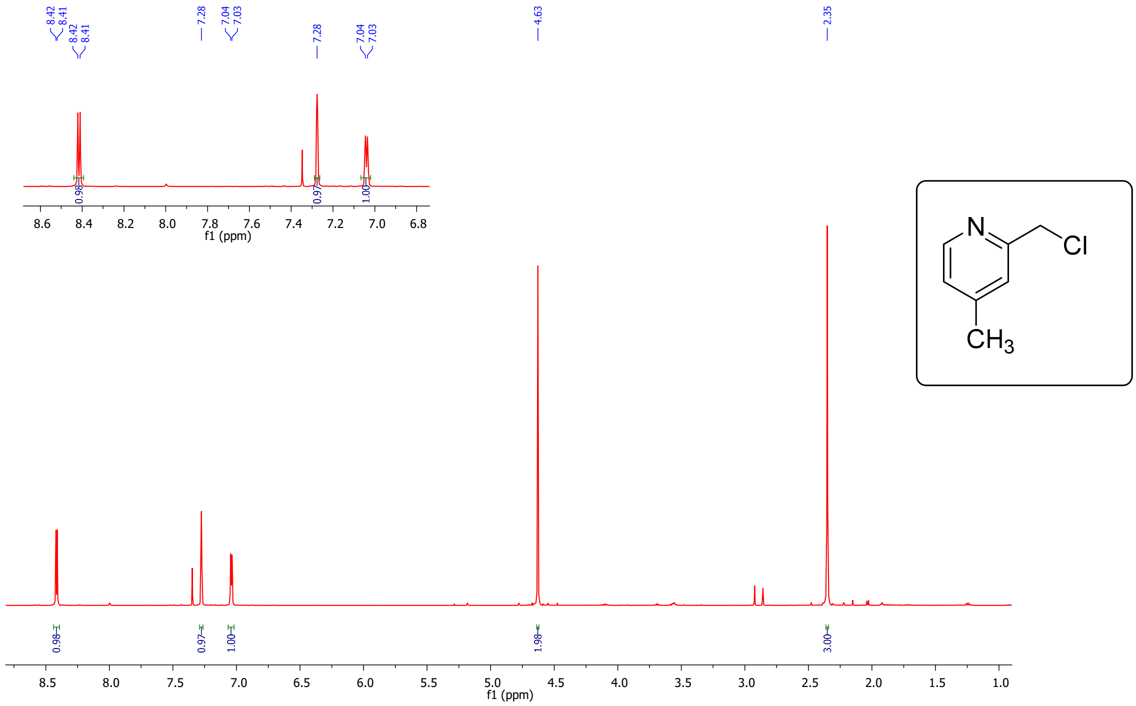

Figure S12. ^{13}C-NMR spectrum of compound **2c** (125 MHz, $CDCl_3$)

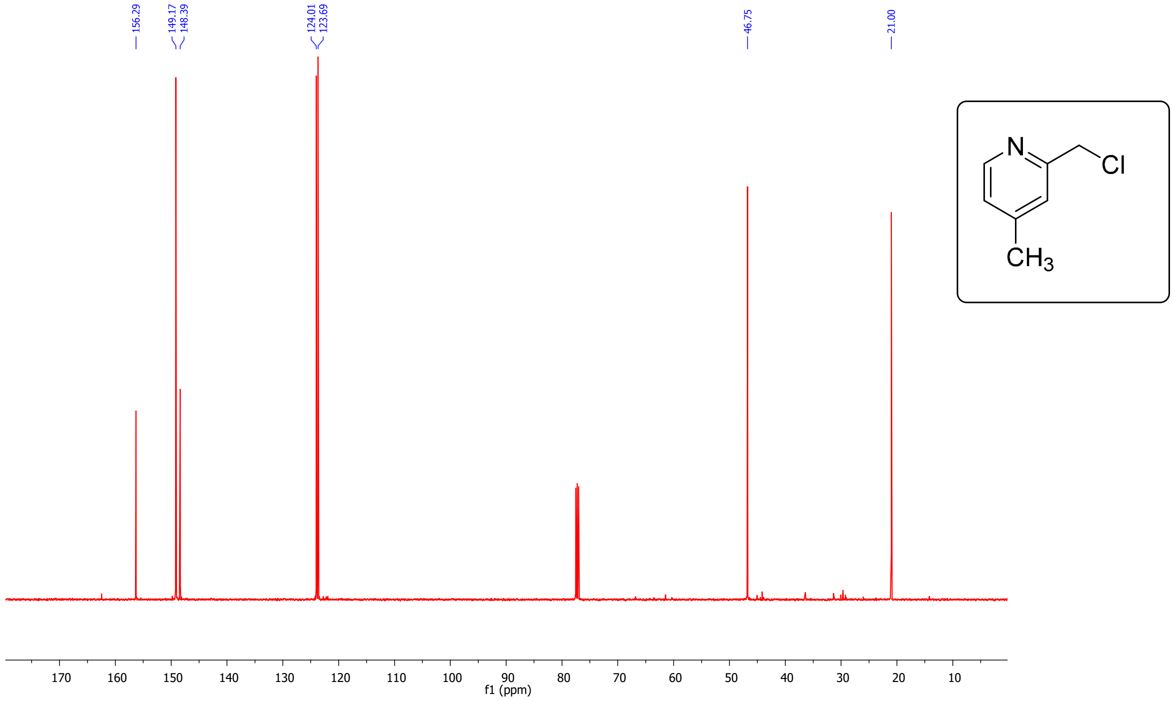

Figure S13. ^{1}H-NMR spectrum of compound **2d** (500 MHz, , $CDCl_3$)

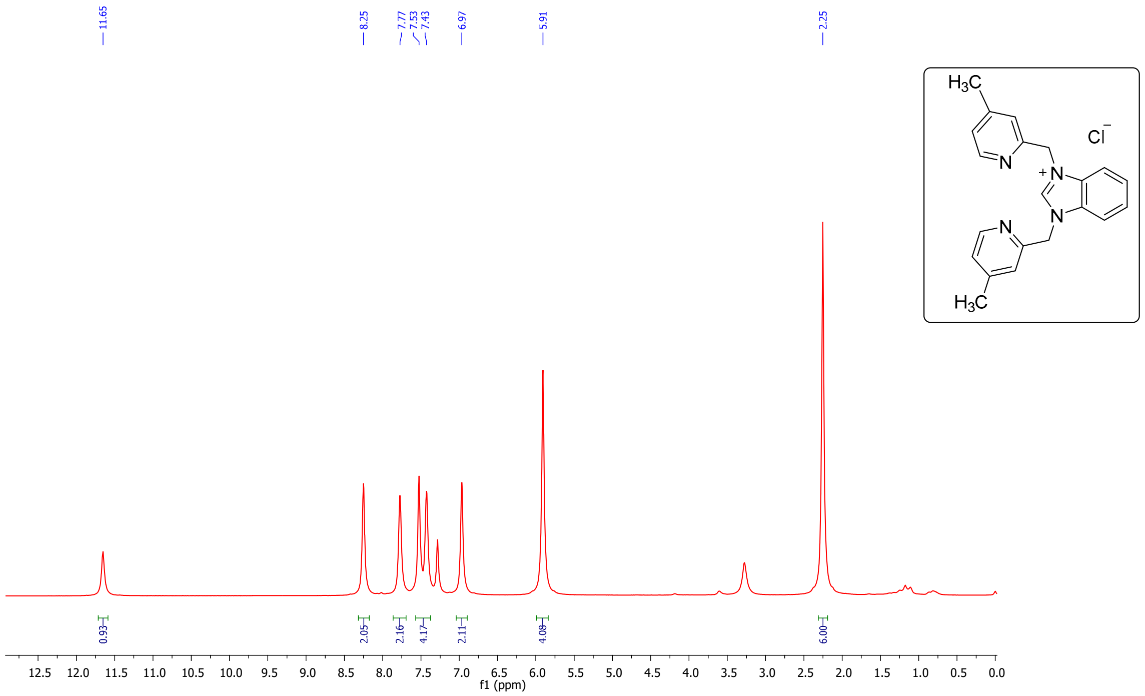

Figure S14. ^{13}C-NMR spectrum of compound **2d** (125 MHz, $CDCl_3$)

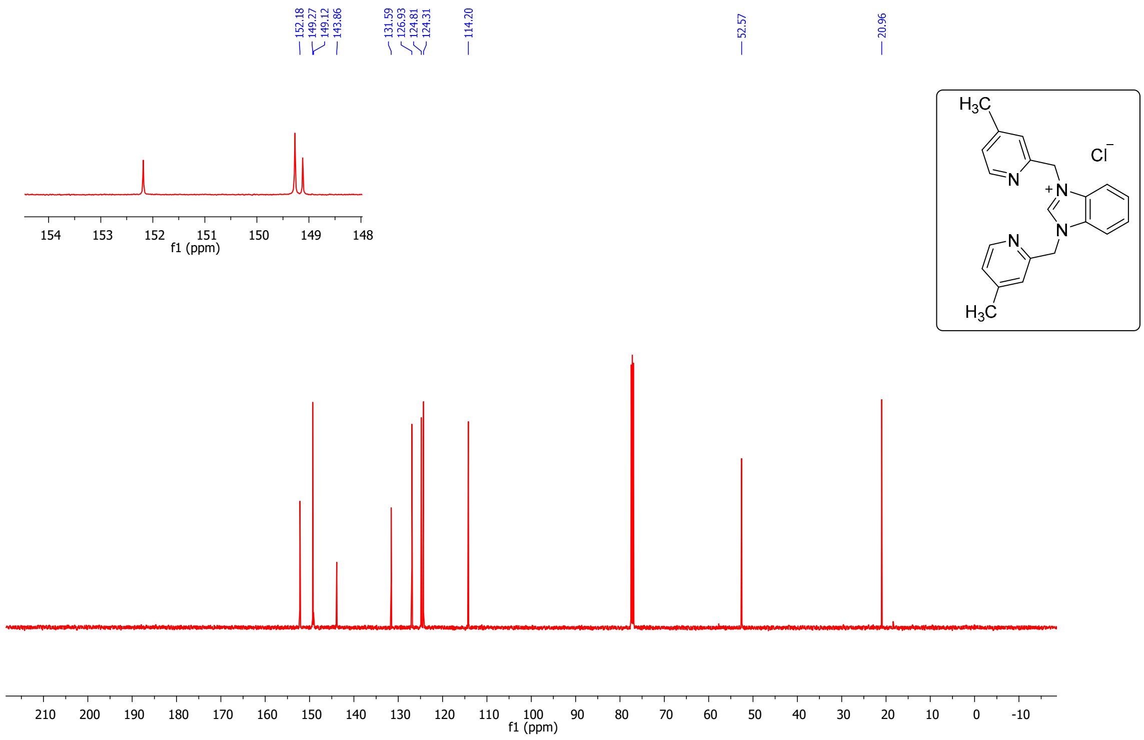

Figure S15. ^{1}H-NMR spectrum of compound **2e** (500 MHz, $CDCl_3$)

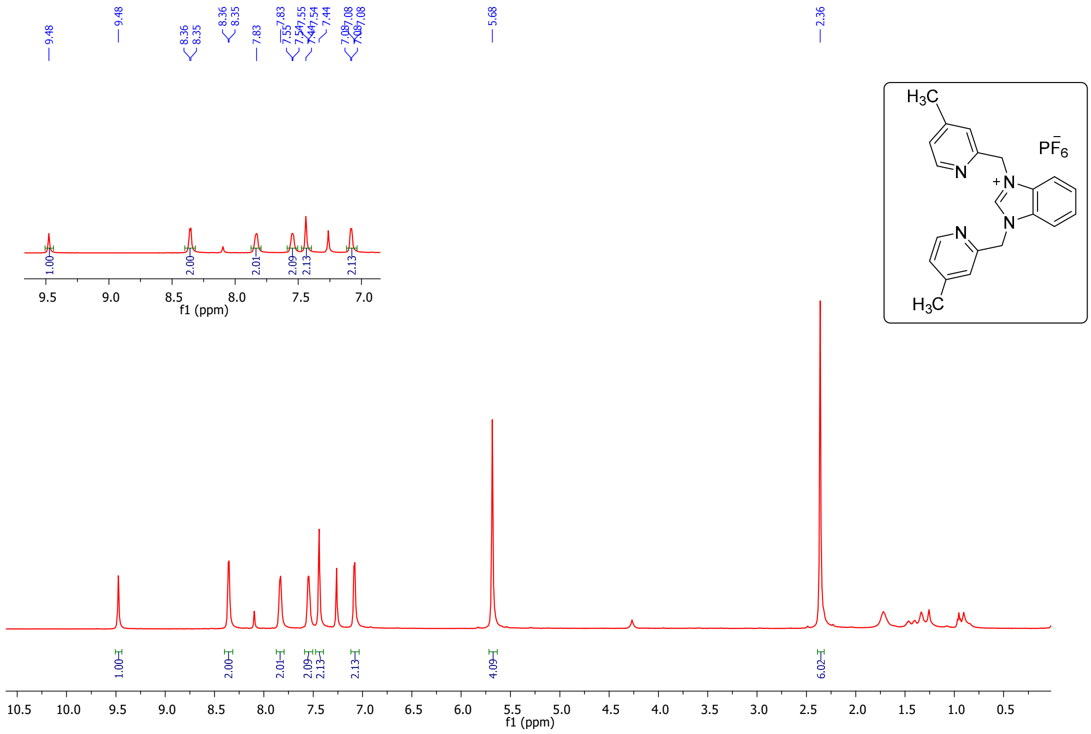

Figure S16. ^{13}C-NMR spectrum of compound **2e** (125 MHz, $CDCl_3$)

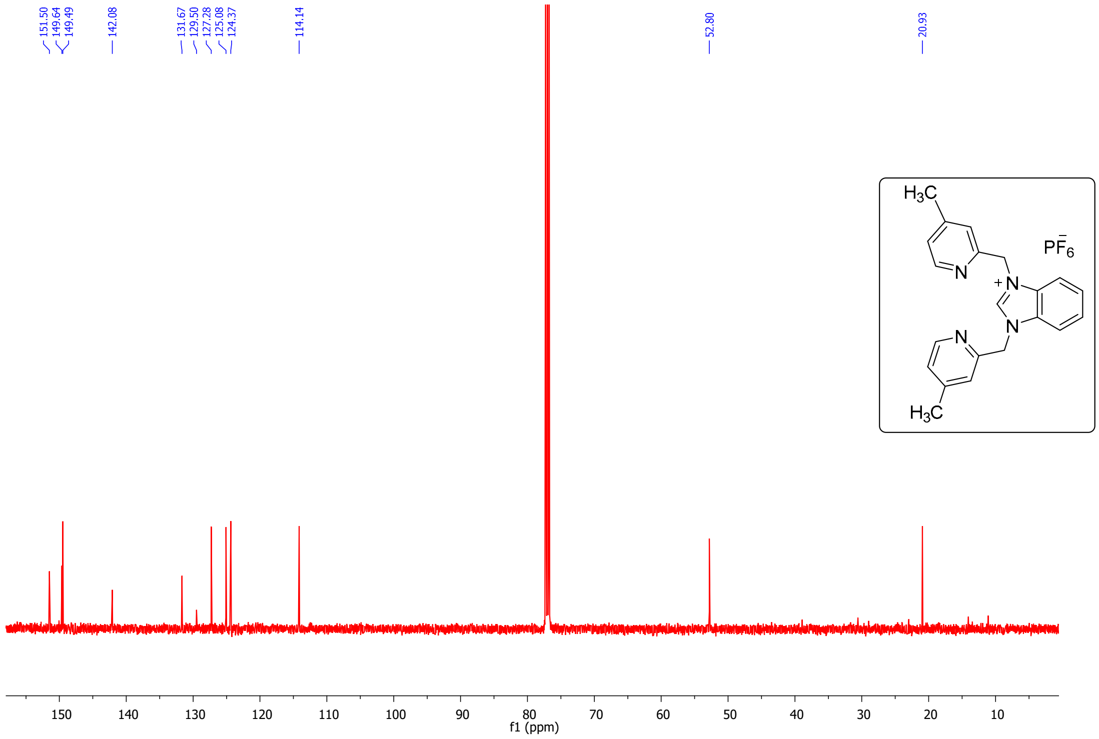

Figure S17. ^{19}F-NMR spectrum of compound **2e** (470 MHz, DMSO-d6)

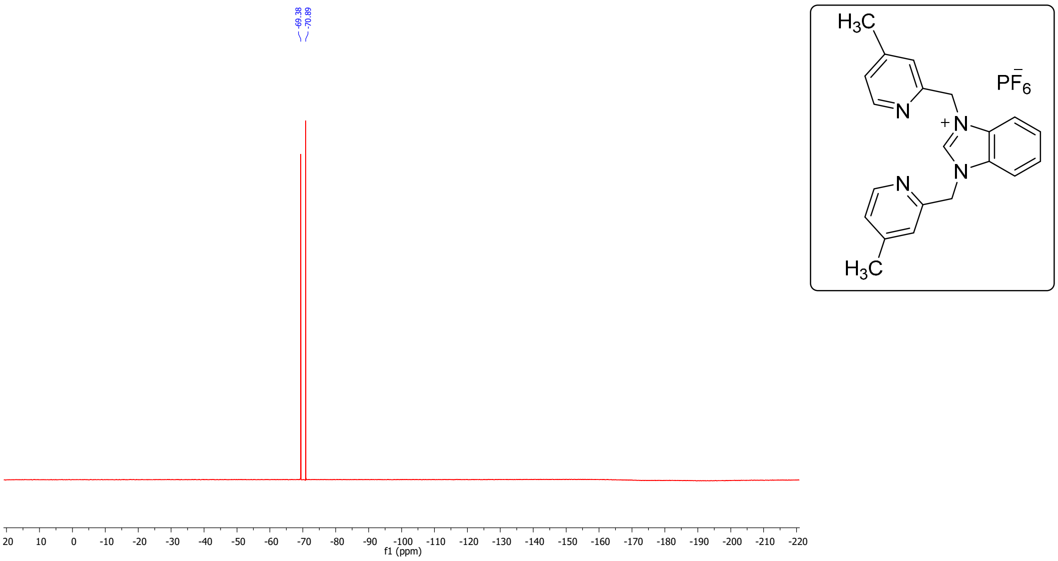

Figure S18. ^{31}P-NMR spectrum of compound **2e** (202 MHz, DMSO-d6)

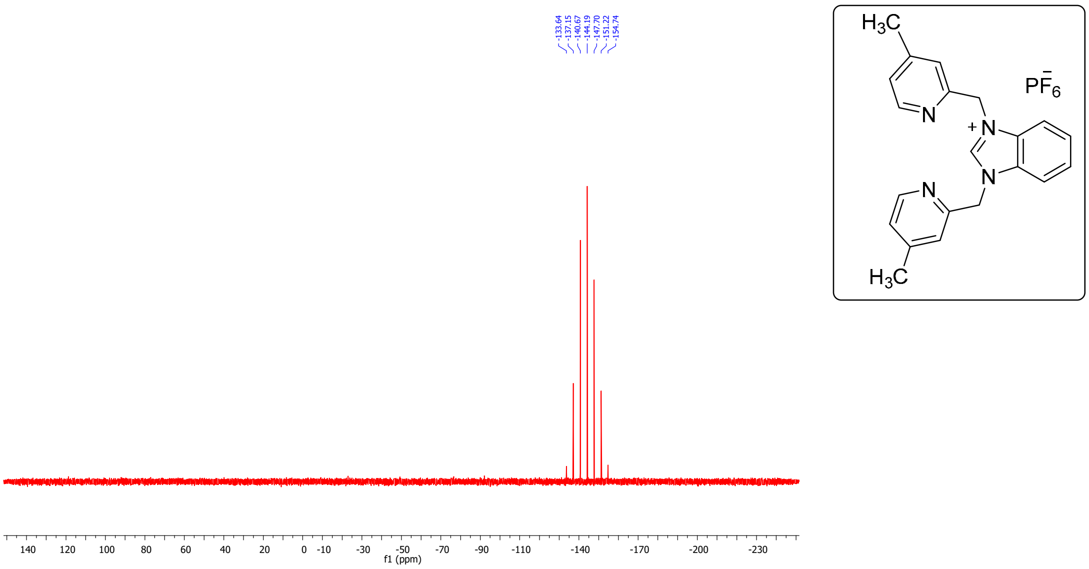

Figure S19. ^{1}H-NMR spectrum of compound **3a** (500 MHz, $CDCl_3$)

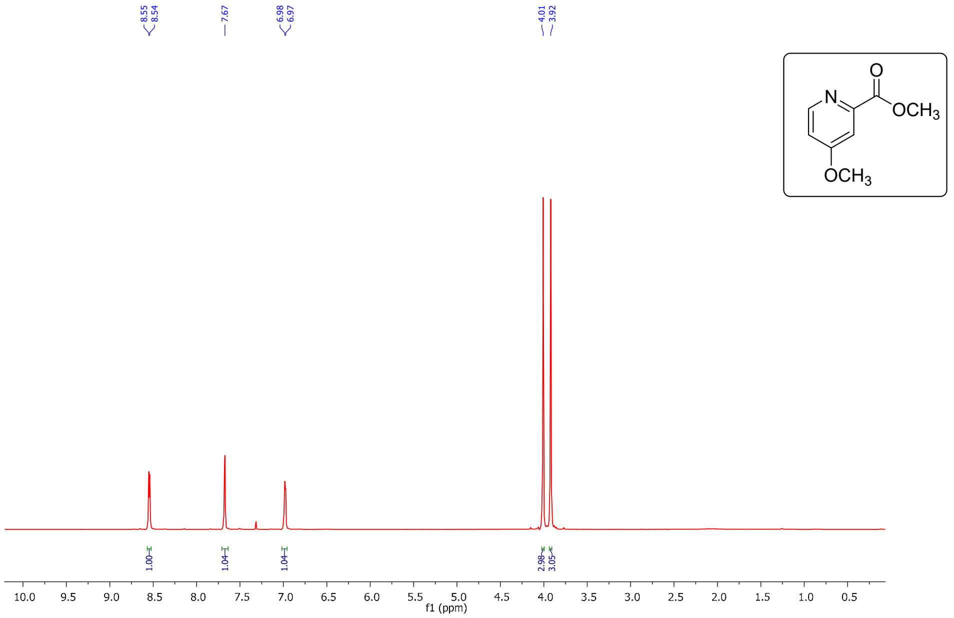

Figure S20. ^{13}C-NMR spectrum of compound **3a** (125 MHz, $CDCl_3$)

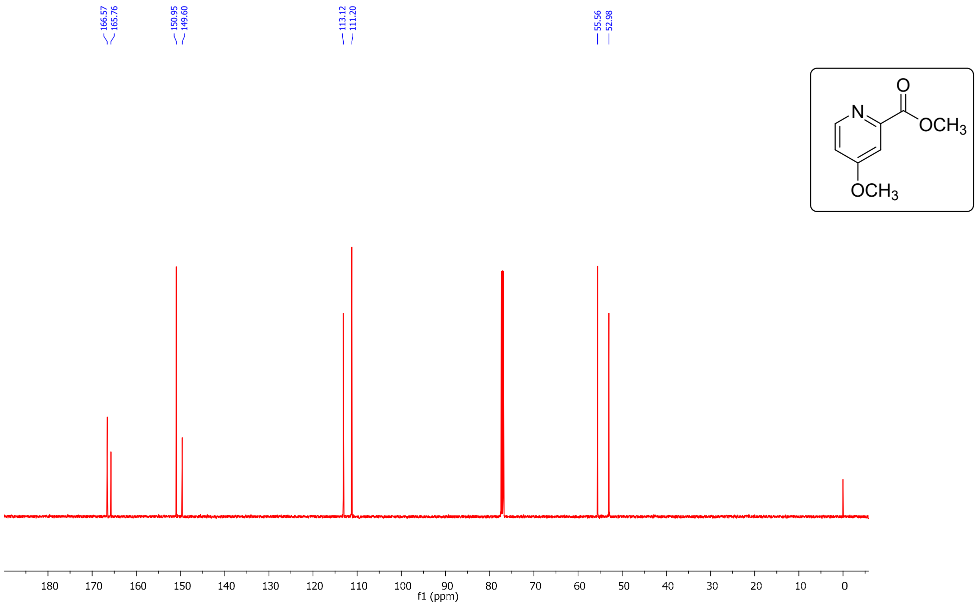

Figure S21. ^{1}H-NMR spectrum of compound **3b** (500 MHz, $CDCl_3$)

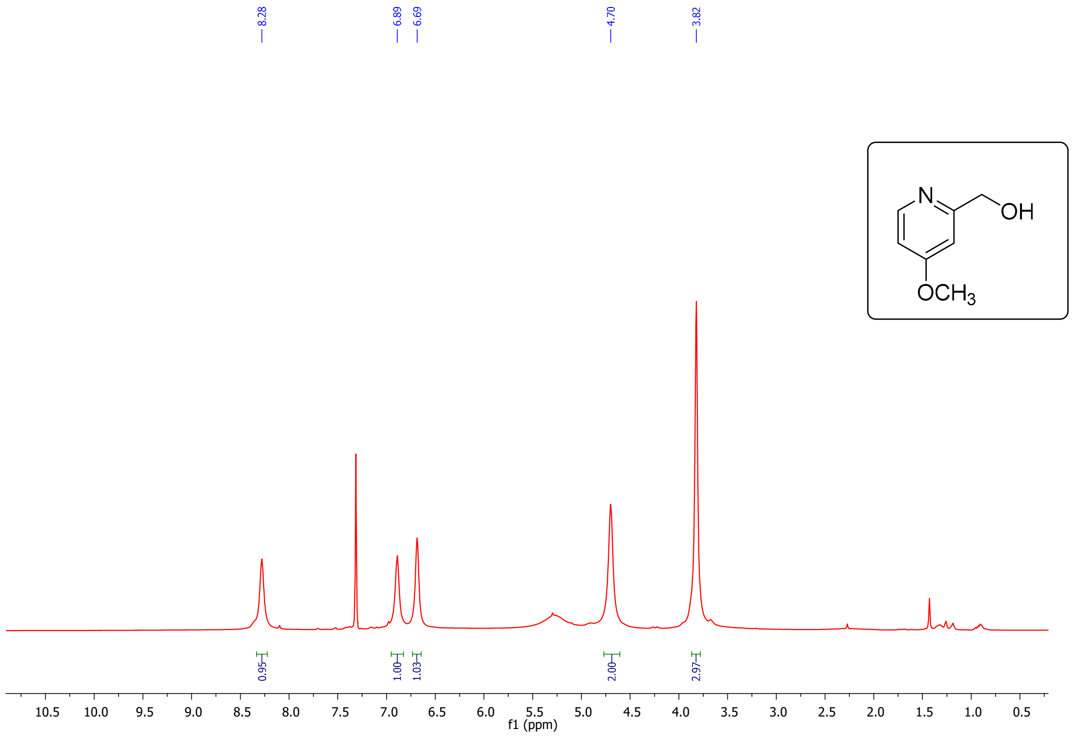

Figure S22. ^{13}C-NMR spectrum of compound **3b** (125 MHz, $CDCl_3$)

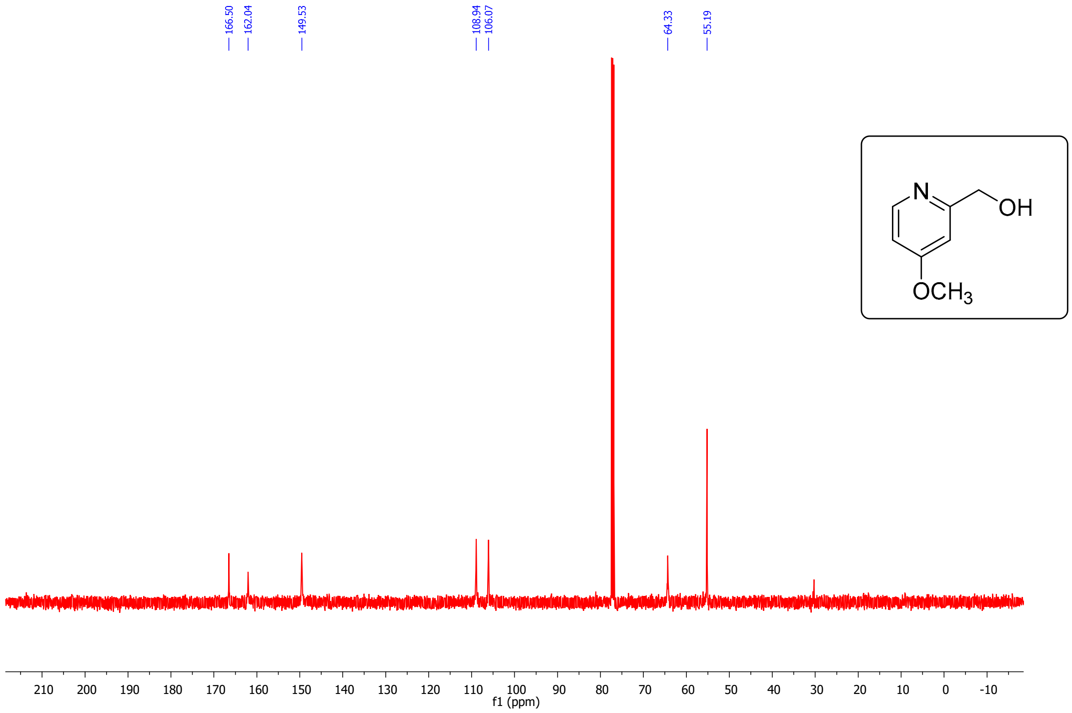

Figure S23. ^{1}H-NMR spectrum of compound **3c** (500 MHz, $CDCl_3$)

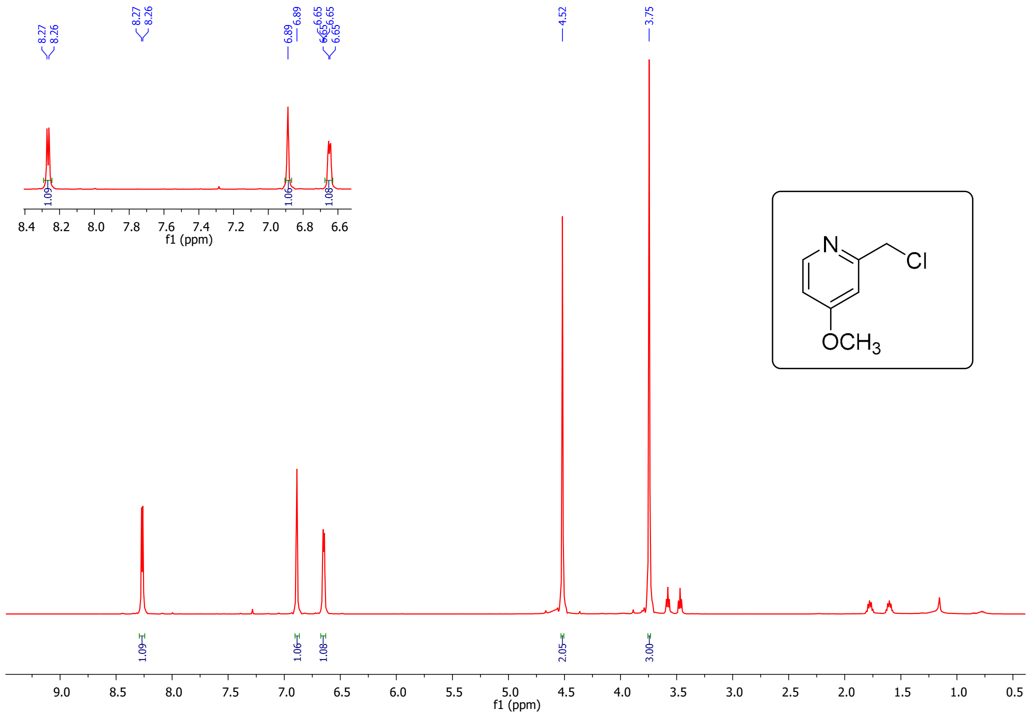

Figure S24. ^{13}C-NMR spectrum of compound **3c** (125 MHz, $CDCl_3$)

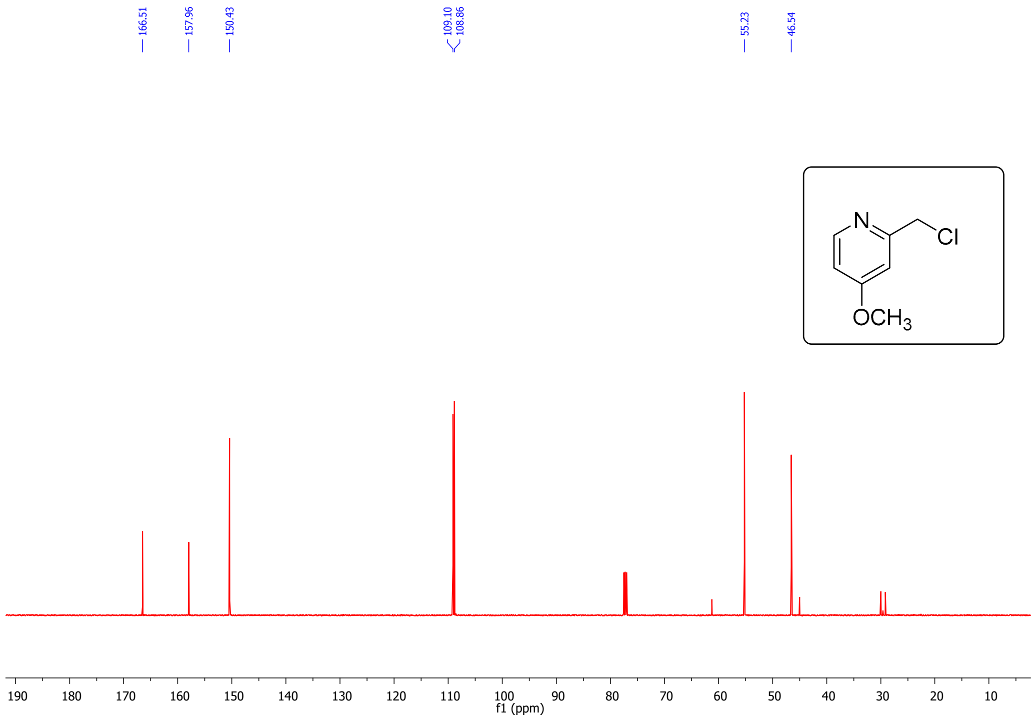

Figure S25. ^{1}H-NMR spectrum of compound **3d** (500 MHz, $CDCl_3$)

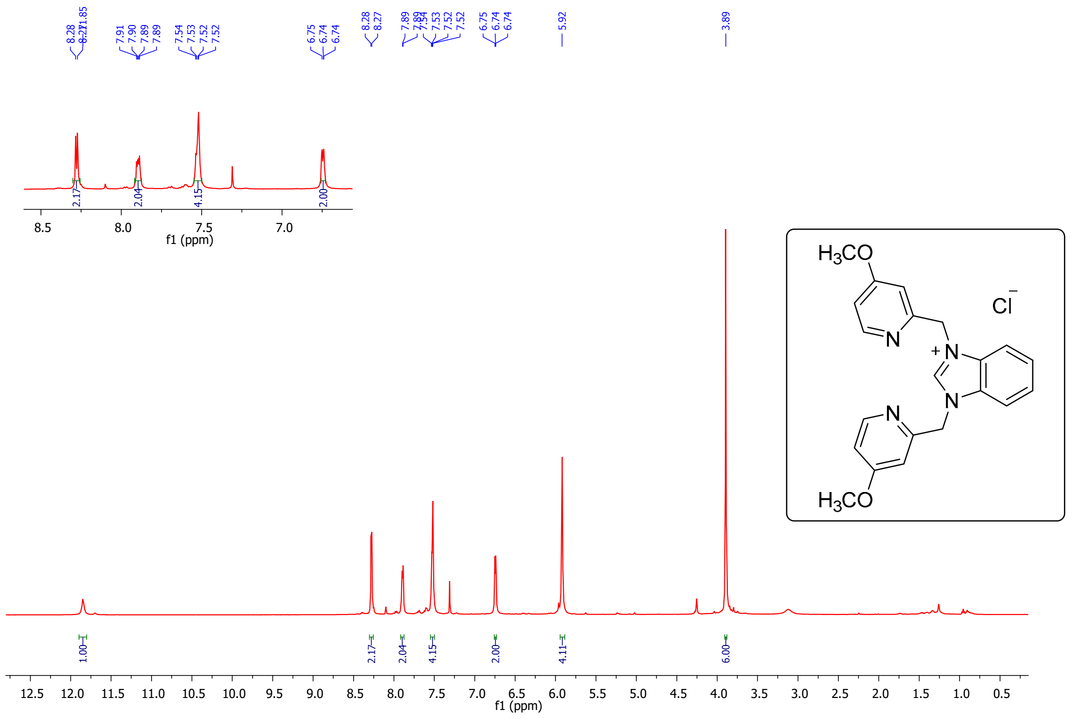

Figure S26. ^{13}C-NMR spectrum of compound **3d** (125 MHz, $CDCl_3$)

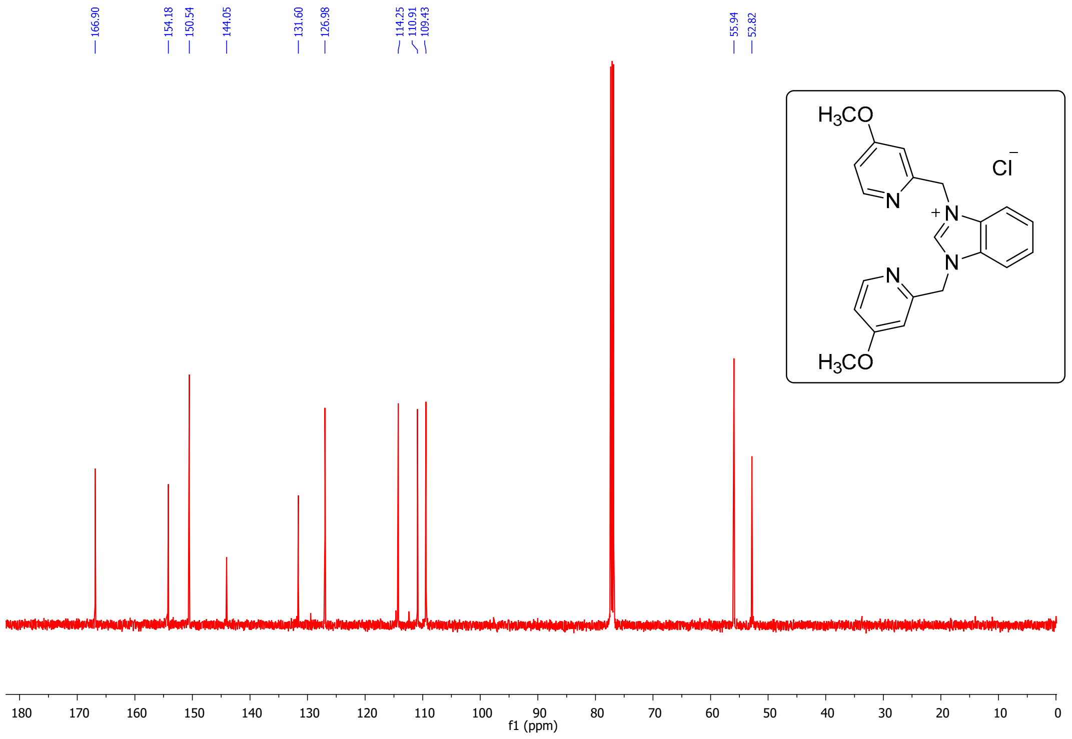

Figure S27. ^{1}H-NMR spectrum of compound **3e** (500 MHz, DMSO-d6)

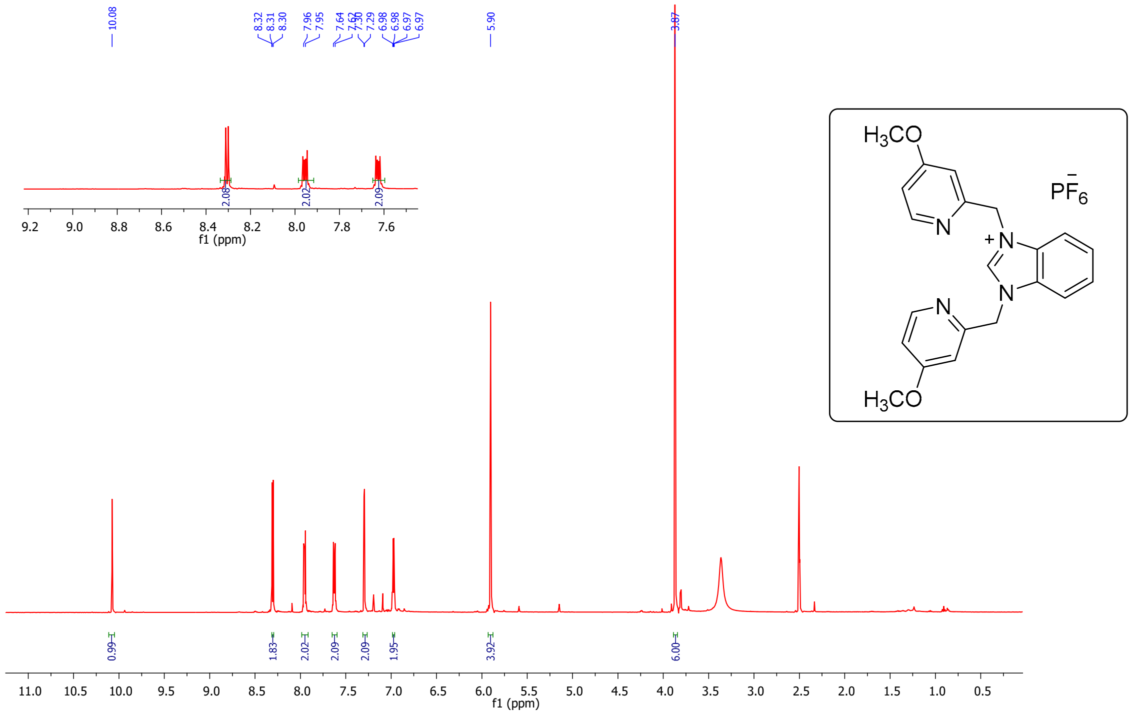

Figure S28. ^{13}C-NMR spectrum of compound **3e** (125 MHz, DMSO-d6)

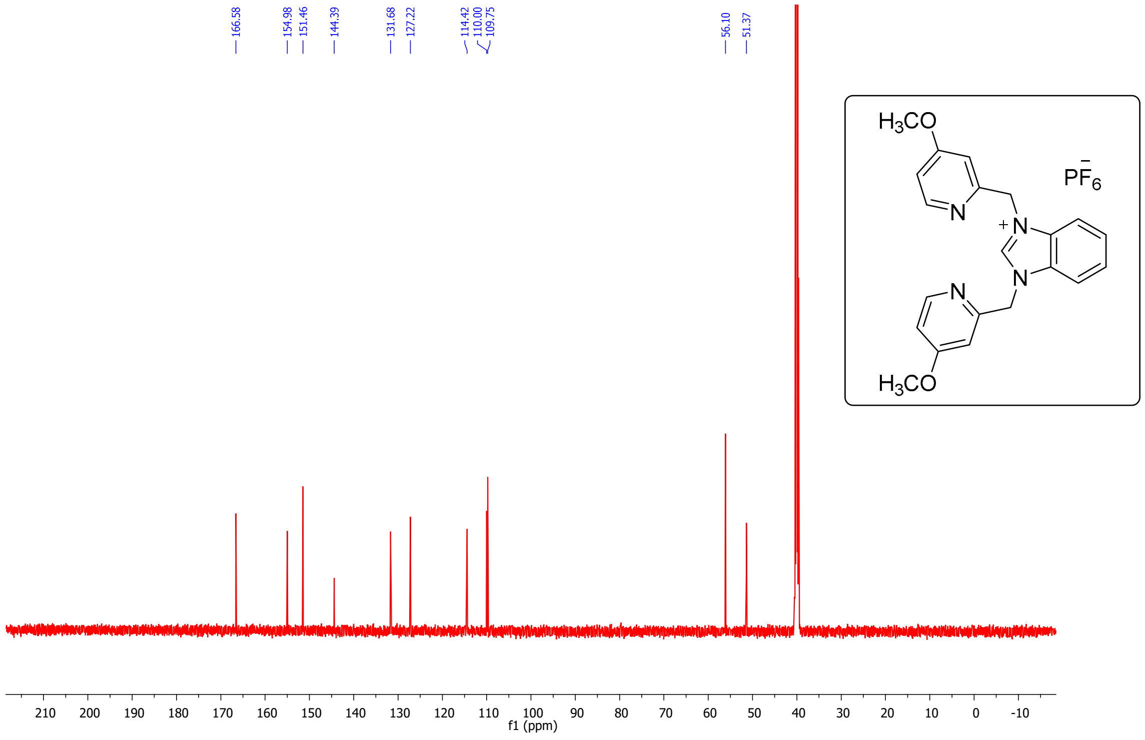

Figure S29. ^{19}F-NMR spectrum of compound **3e** (470 MHz, DMSO-d_6)

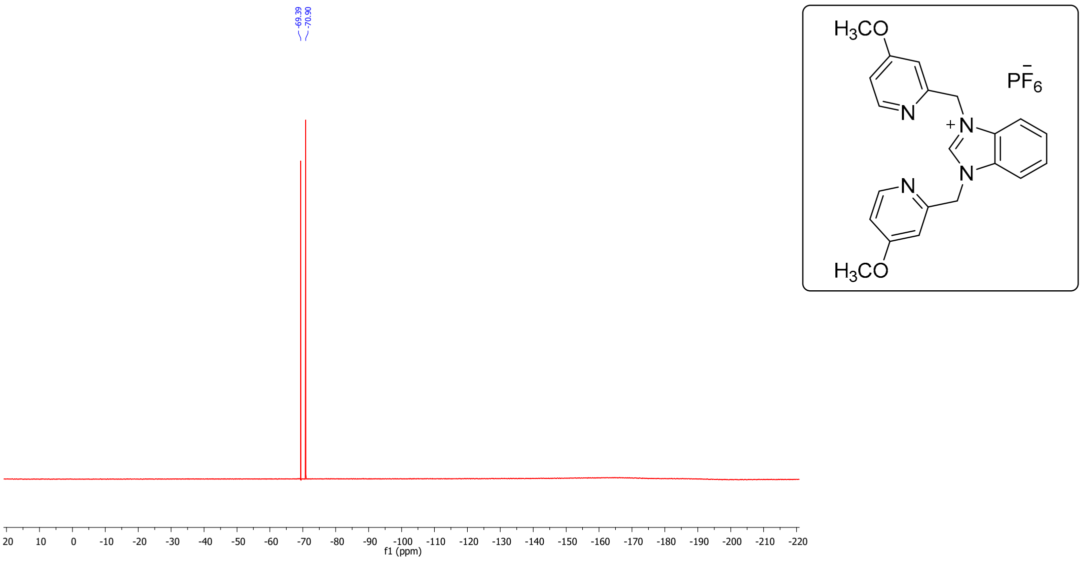

Figure S30. ^{31}P-NMR spectrum of compound **3e** (202 MHz, DMSO-d_6)

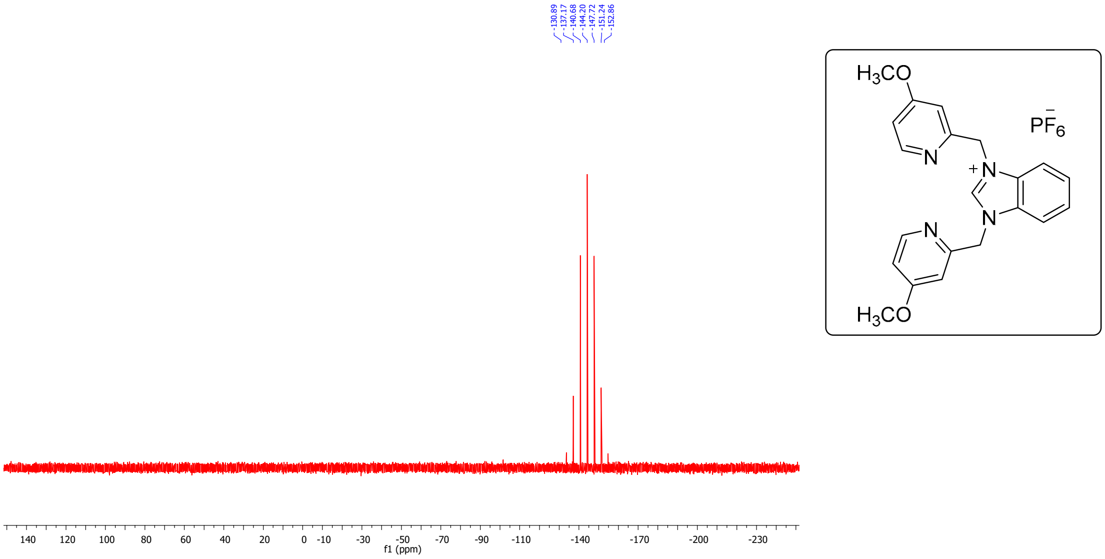

Figure S31. ^{1}H-NMR spectrum of compound **4a** (500 MHz, $CDCl_3$)

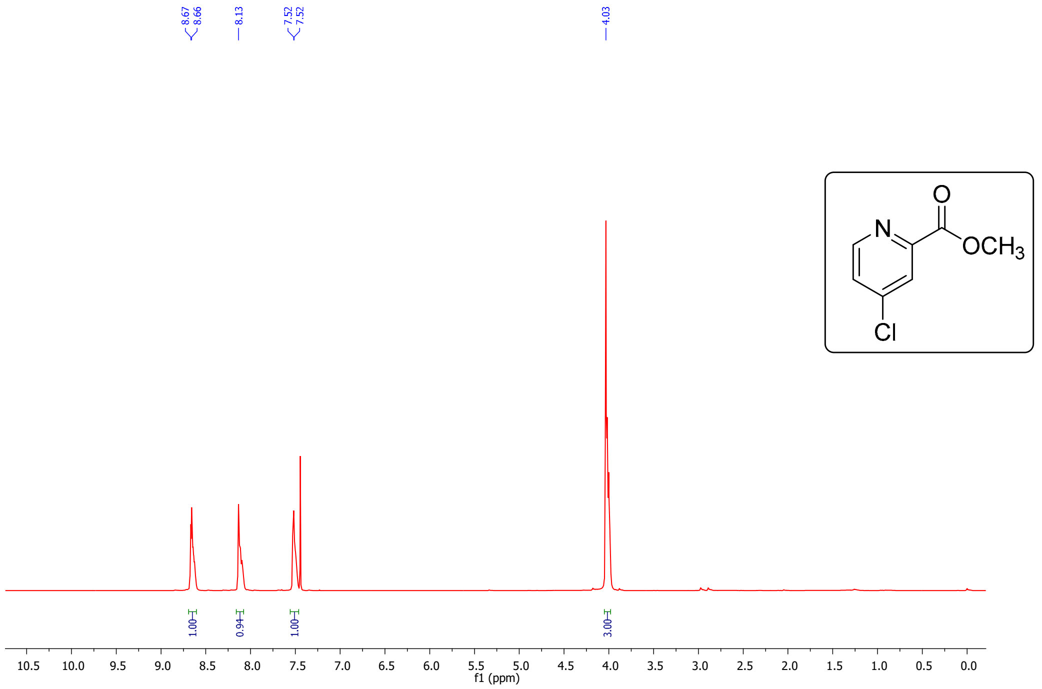

Figure S32. ^{13}C-NMR spectrum of compound **4a** (125 MHz, $CDCl_3$)

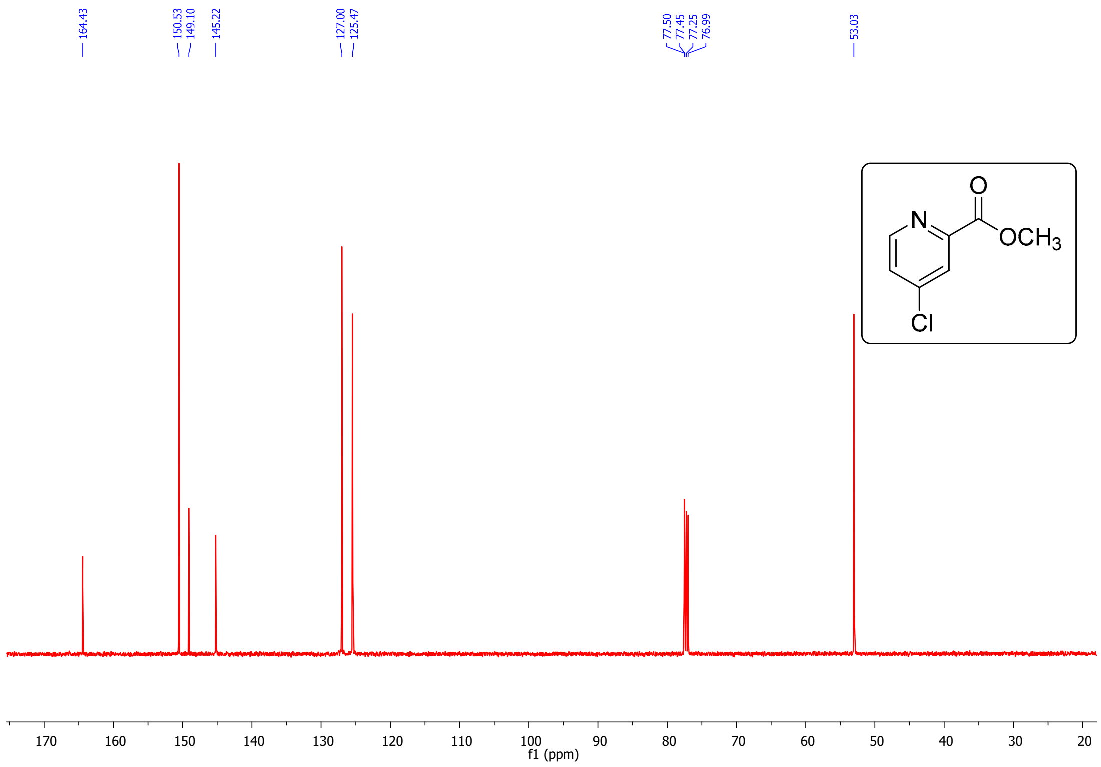

Figure S33. ^{1}H-NMR spectrum of compound **4b** (500 MHz, $CDCl_3$)

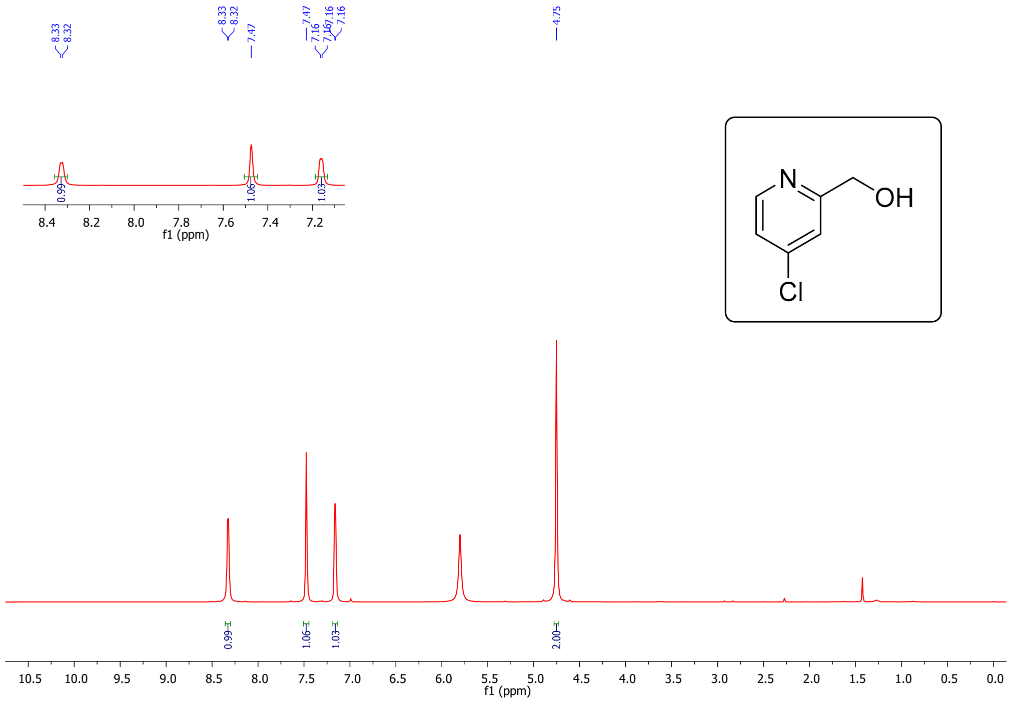

Figure S34. ^{13}C-NMR spectrum of compound **4b** (125 MHz, $CDCl_3$)

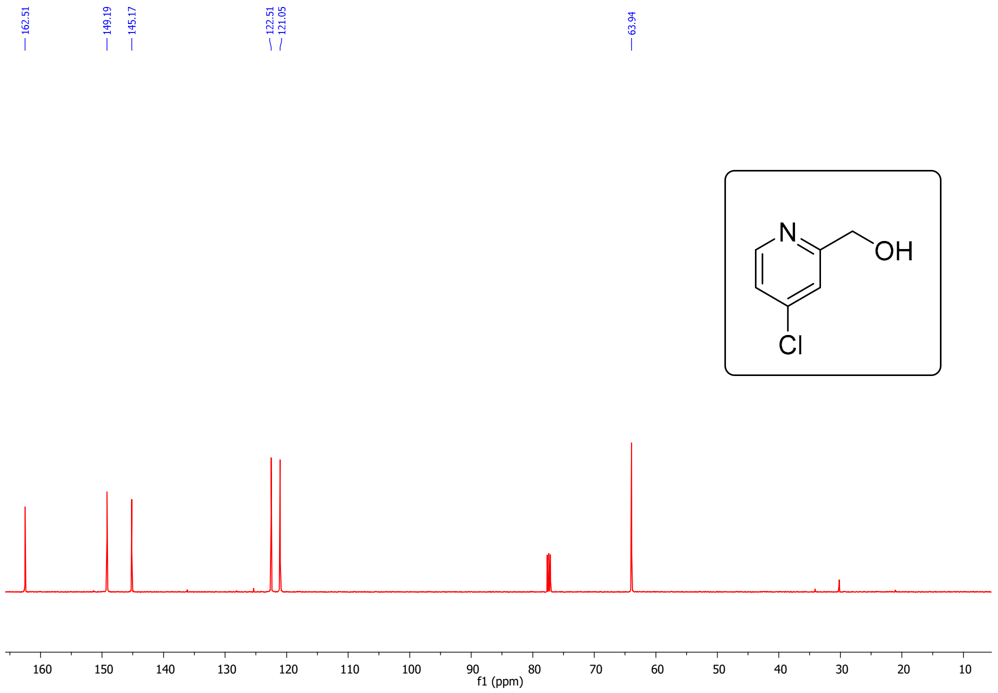

Figure S35. ^{1}H-NMR spectrum of compound **4c** (500 MHz, $CDCl_3$)

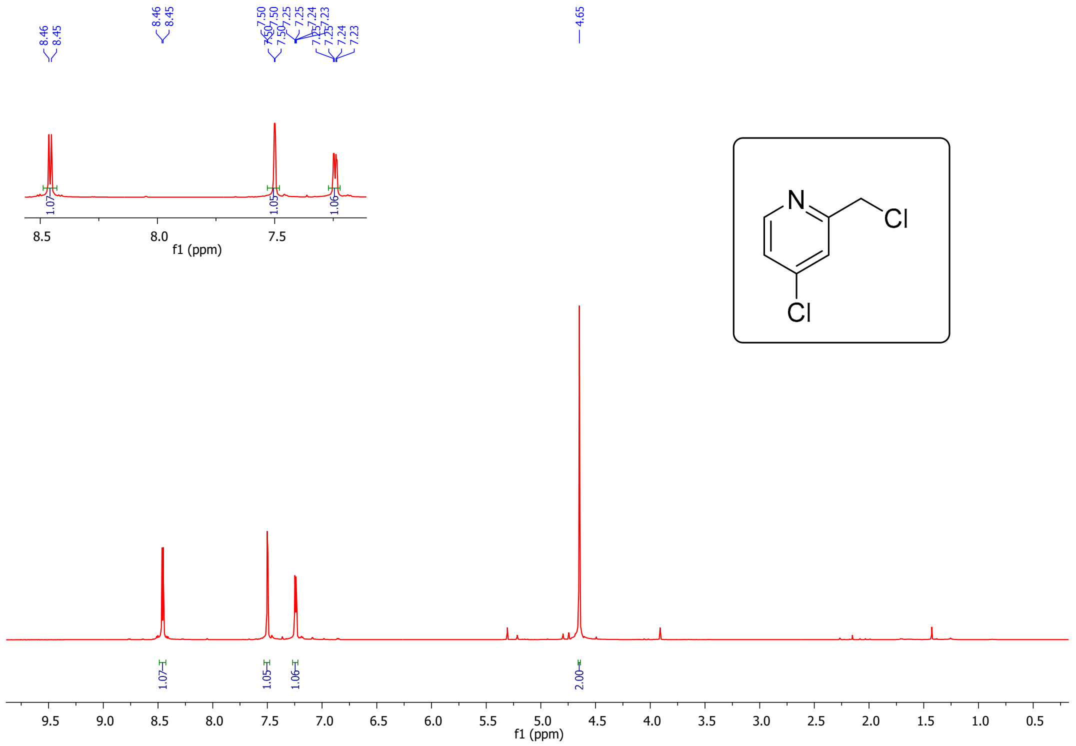

Figure S36. ^{13}C-NMR spectrum of compound **4c** (125 MHz, $CDCl_3$)

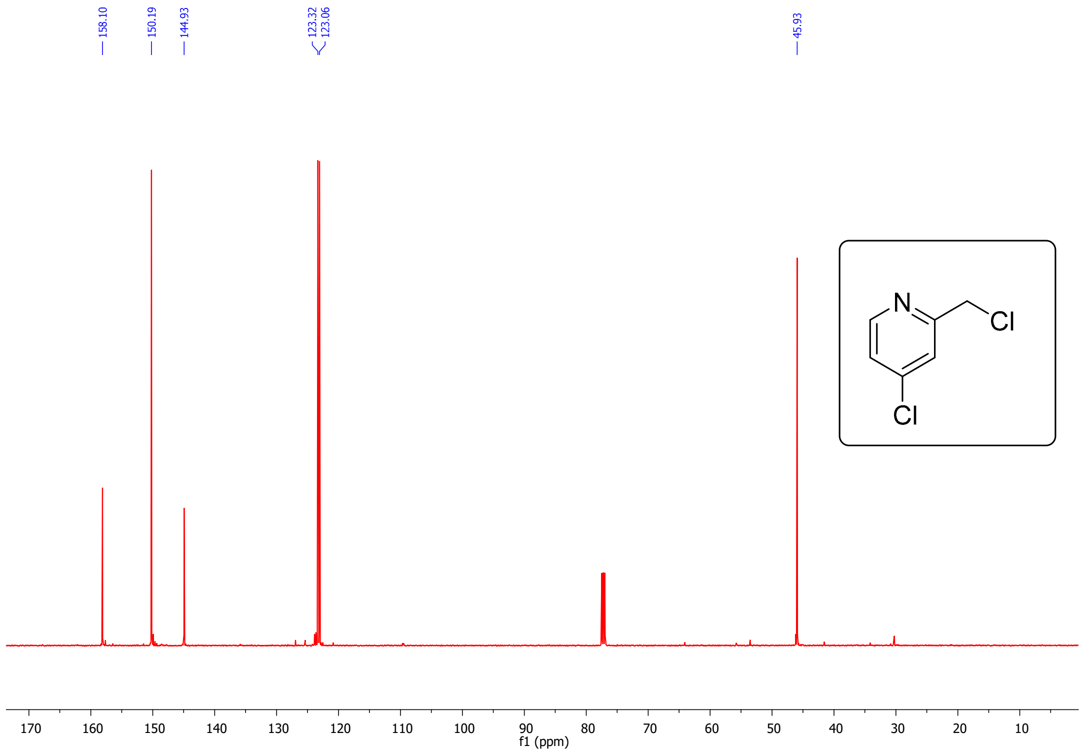

Figure S37. ^{1}H-NMR spectrum of compound **4d** (500 MHz, DMSO-d$_6$)

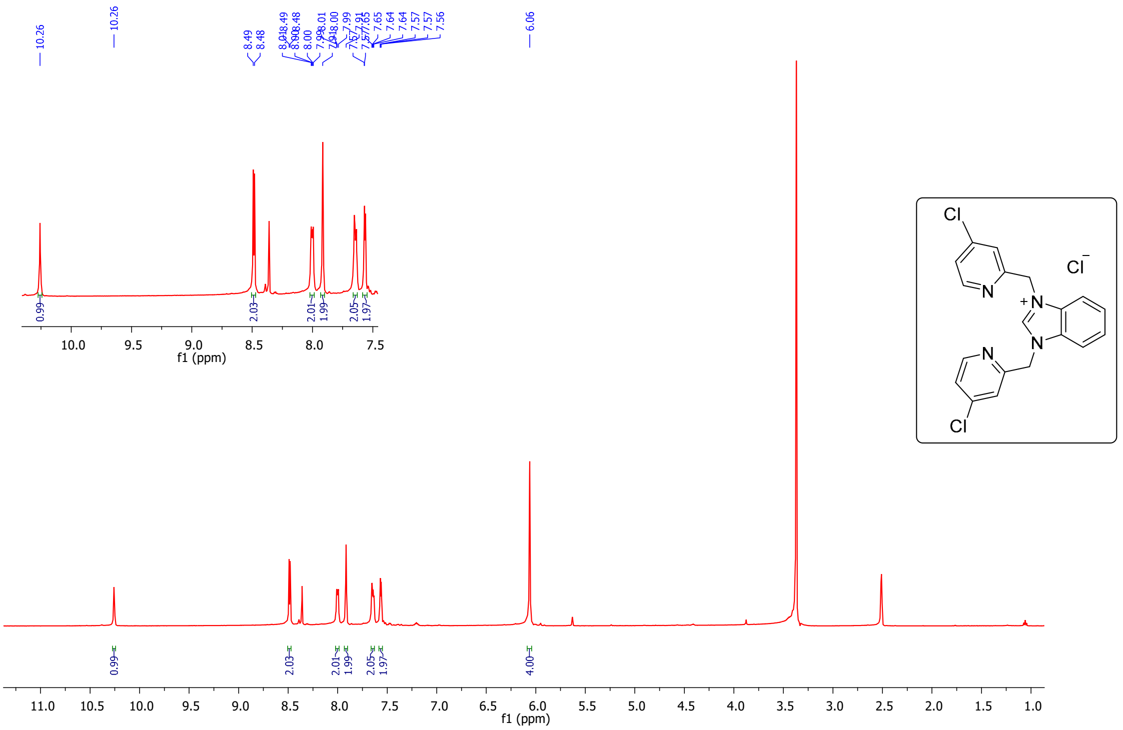

Figure S38. ^{13}C-NMR spectrum of compound **4d** (125 MHz, DMSO-d$_6$)

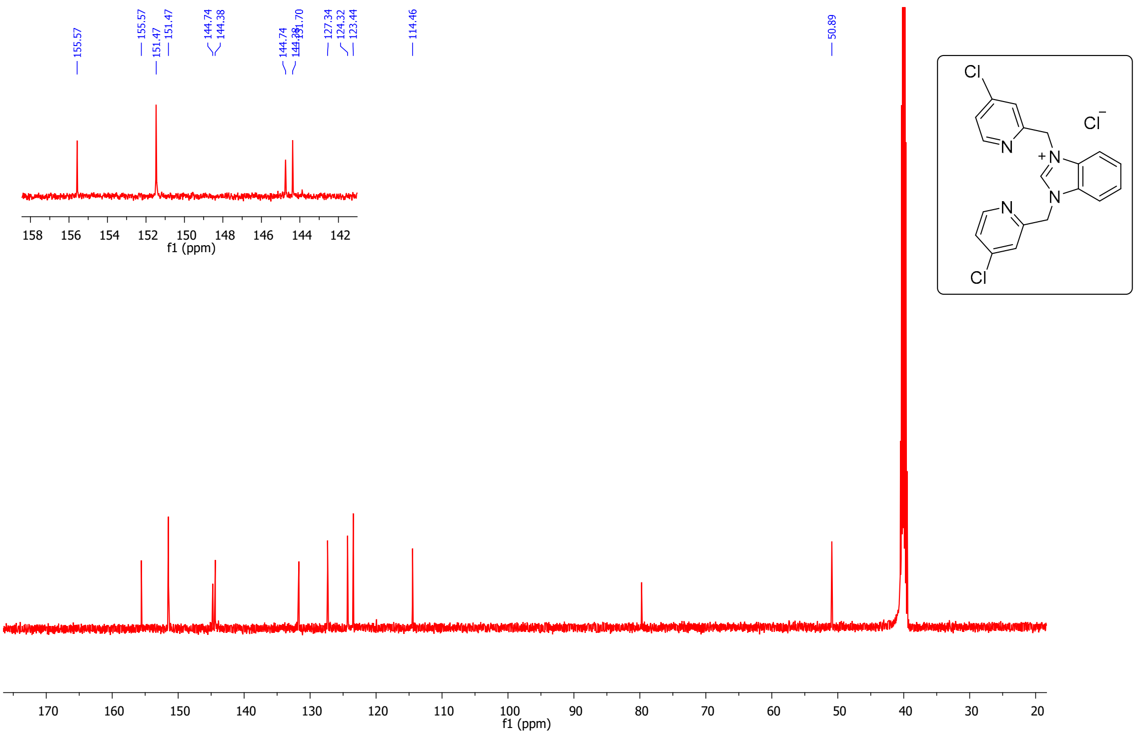

Figure S39. ^{1}H-NMR spectrum of compound **4e** (500 MHz, DMSO-d6)

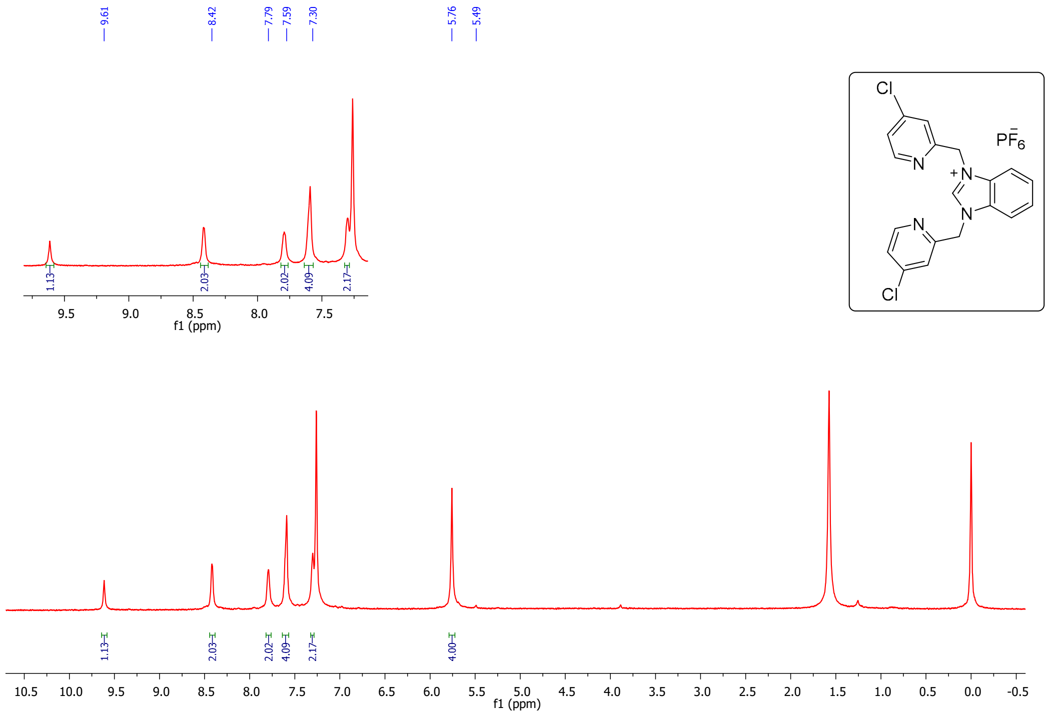

Figure S40. ^{13}C-NMR spectrum of compound **4e** (125 MHz, DMSO-d6)

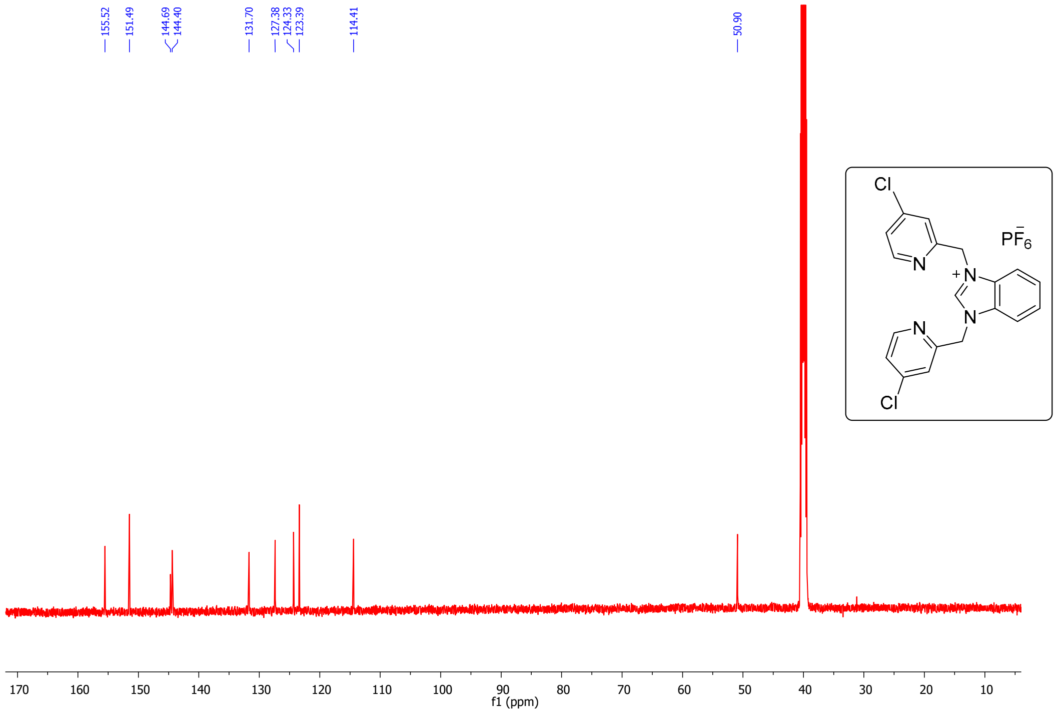

Figure S41. ^{19}F-NMR spectrum of compound **4e** (470 MHz, DMSO-d6)

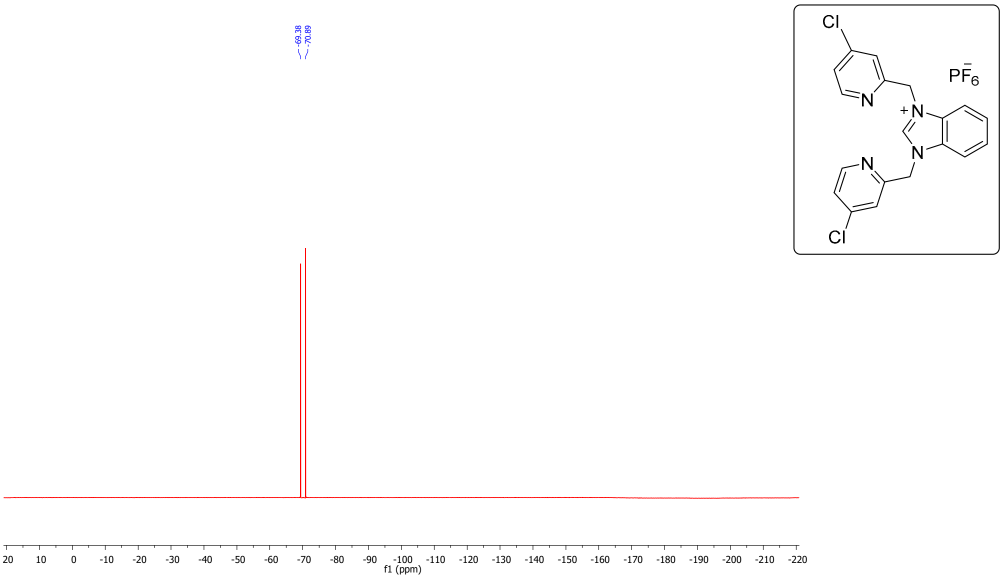

Figure S42. ^{31}P-NMR spectrum of compound **4e** (202 MHz, DMSO-d6)

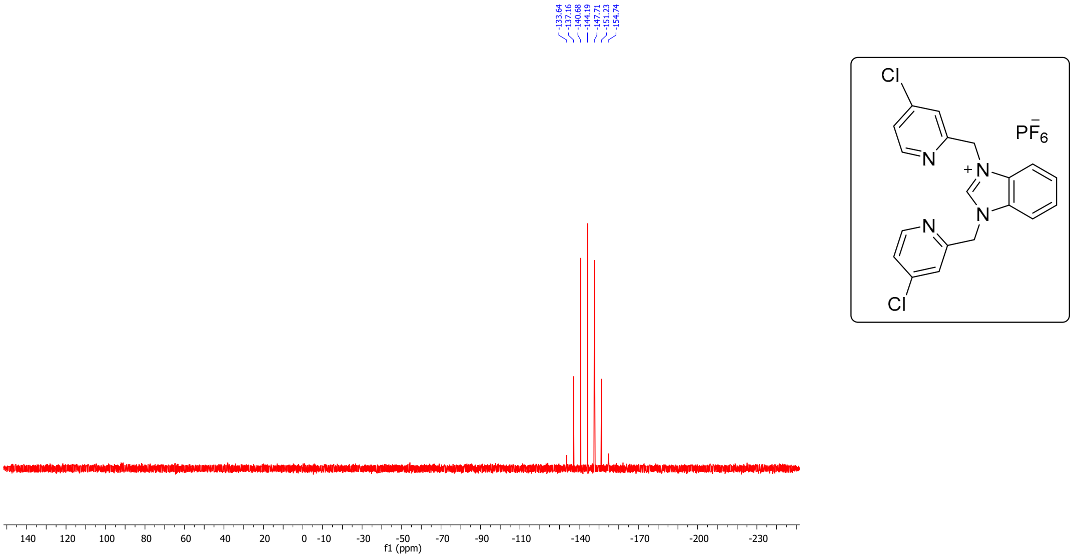

Figure S43. HRMS-ESI spectrum of compound **bPymBI-Me**

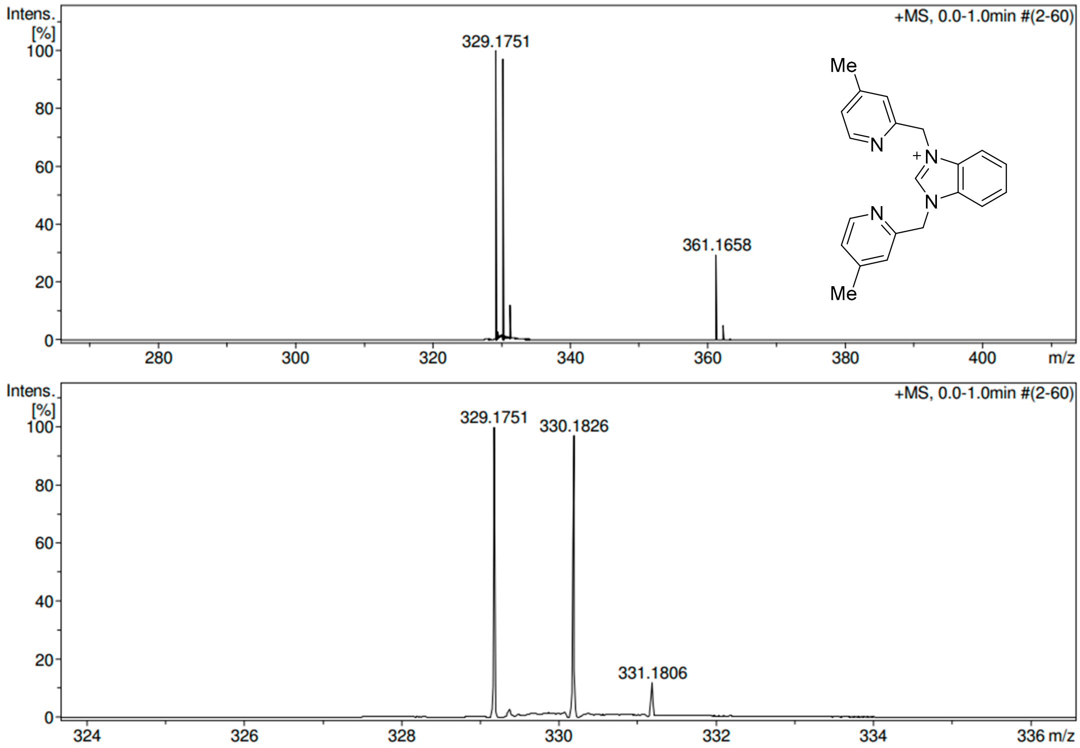

Figure S44. HRMS-ESI spectrum of compound **bPymBI-OMe**

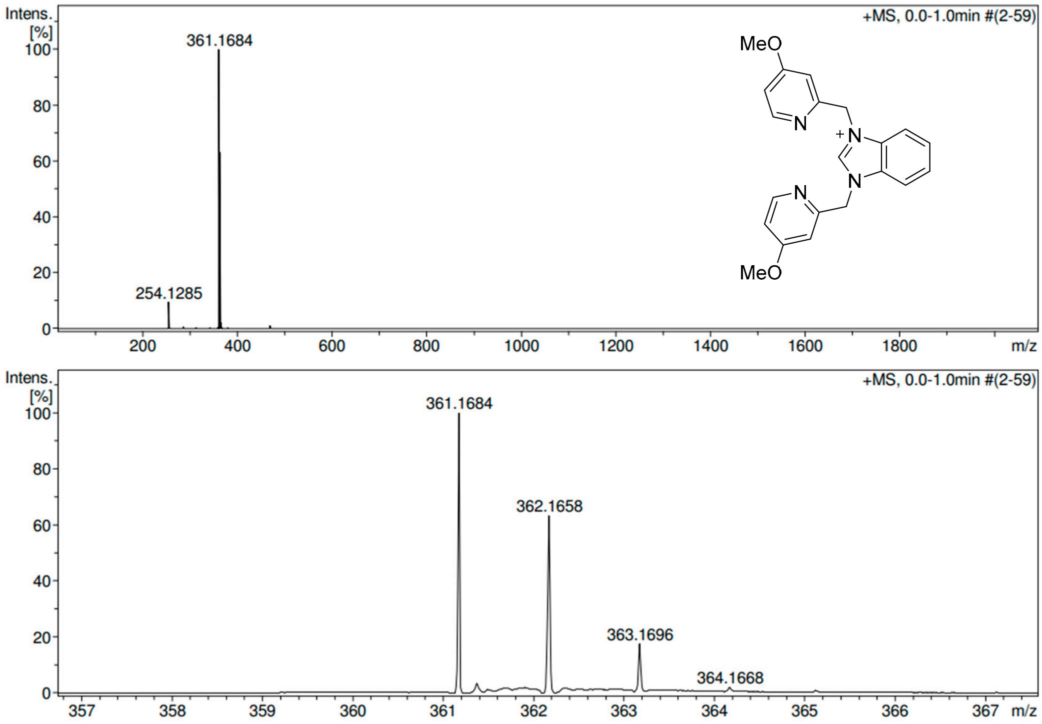

Figure S45. HRMS-ESI spectrum of compound **bPymBI-Cl**

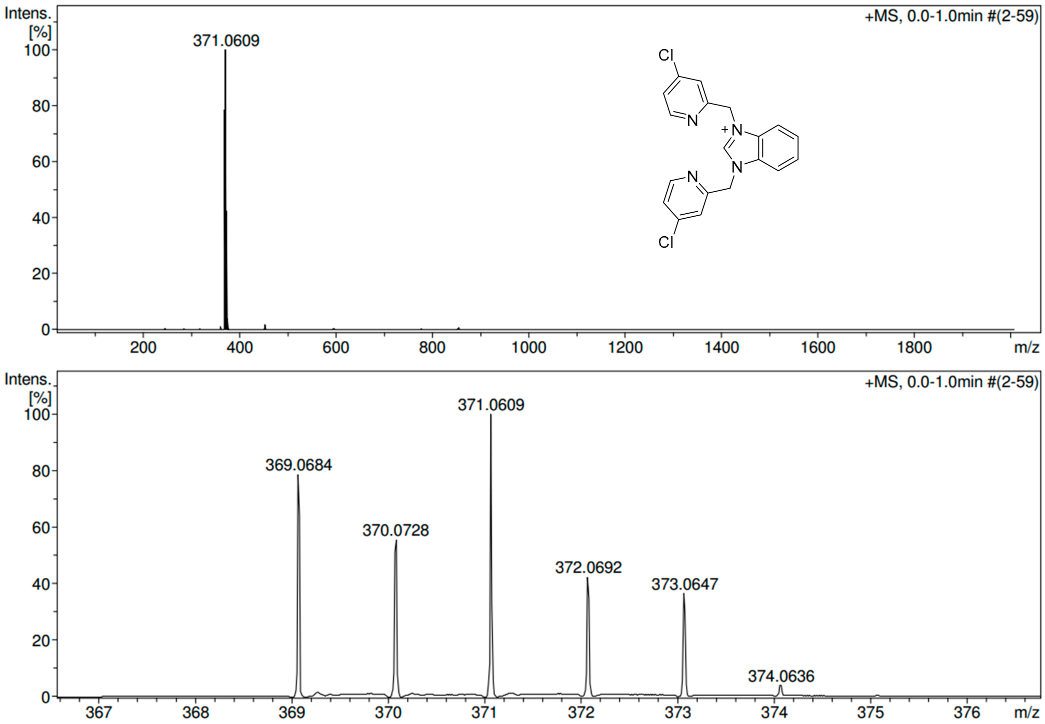

Figure S46. XRD of compound **Cu^{2+}bPymBI-Me [CCDC: 2313922]**

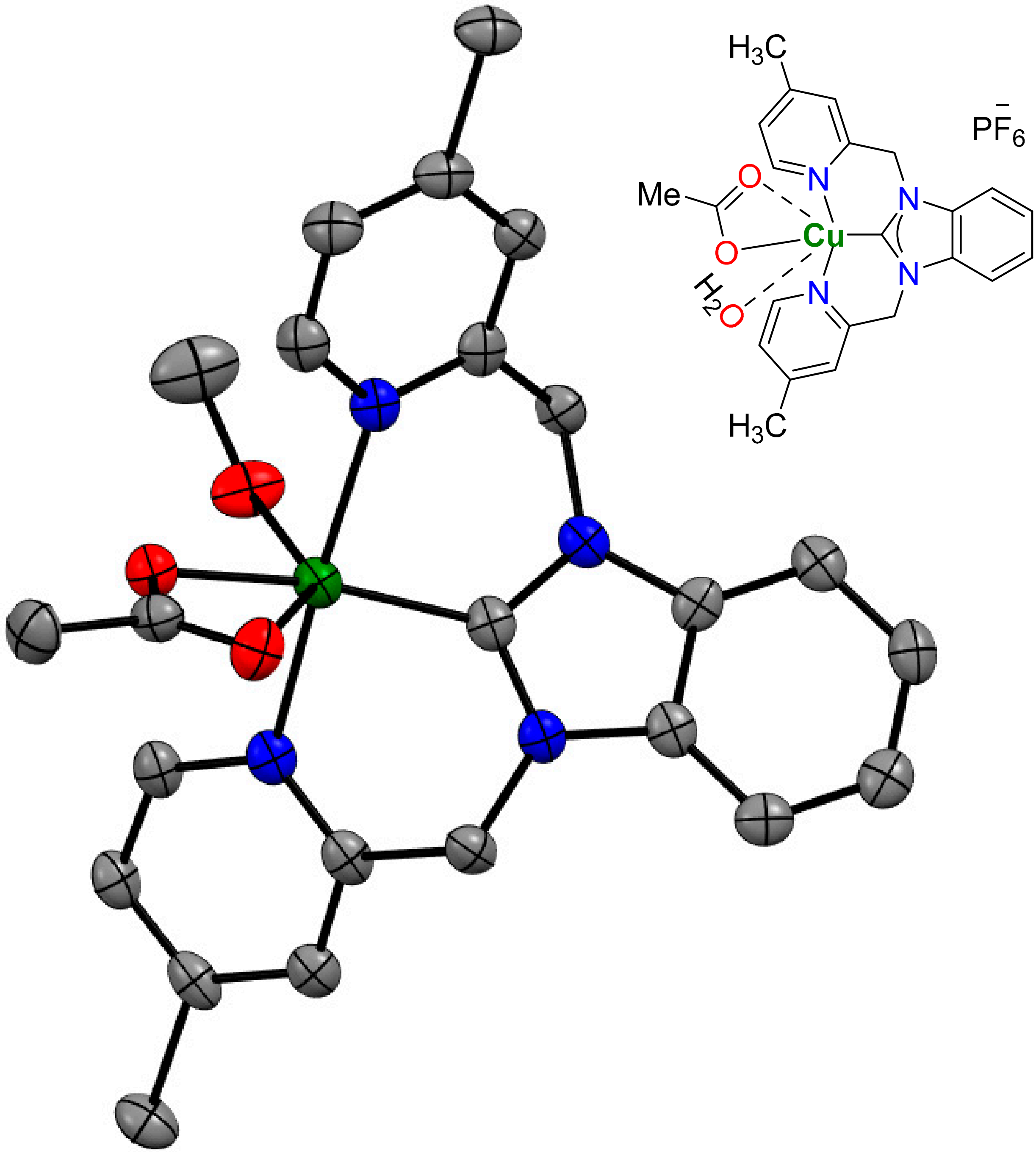

Table S1. Crystal data and structure refinement for **Cu^{2+}bPymBI-Me [CCDC: 2313922]**

Empirical formula	$C_{24}H_{27}CuF_6N_4PO_3$
Formula weight	628.00
Temperature/K	160.0(2)
Crystal system	Triclinic
Space group	P-1
a/Å	7.2339(5)
b/Å	13.0210(8)
c/Å	14.9669(12)
α/°	71.518(6)
β/°	77.528(7)
γ/°	79.771(5)
Volume/Å^3	1296.41(17)
Z	2
ρ_{calc}g/cm^3	1.609
μ/mm^{-1}	0.982
F(000)	642.0
Crystal size/mm^3	(0.70 × 0.40 × 0.30) mm^3
Radiation	MoKα (λ = 0.71073)
2Θ range for data collection/°	5.012 to 61.768
Index ranges	$-10 \le h \le 10$, $-17 \le k \le 16$, $-18 \le l \le 20$
Reflections collected	21028
Independent reflections	6504 [R_{int} = 0.0257, R_{sigma} = 0.0243]
Data/restraints/parameters	6504/0/360
Goodness-of-fit on F^2	1.068
Final R indexes [I>=2σ (I)]	R_1 = 0.0299, wR_2 = 0.0828
Final R indexes [all data]	R_1 = 0.0326, wR_2 = 0.0841
Largest diff. peak/hole / e Å^{-3}	0.61/-0.53

Figure S47. XRD of compound **Cu^{2+}bPymBI-OMe [CCDC: 2313921]**

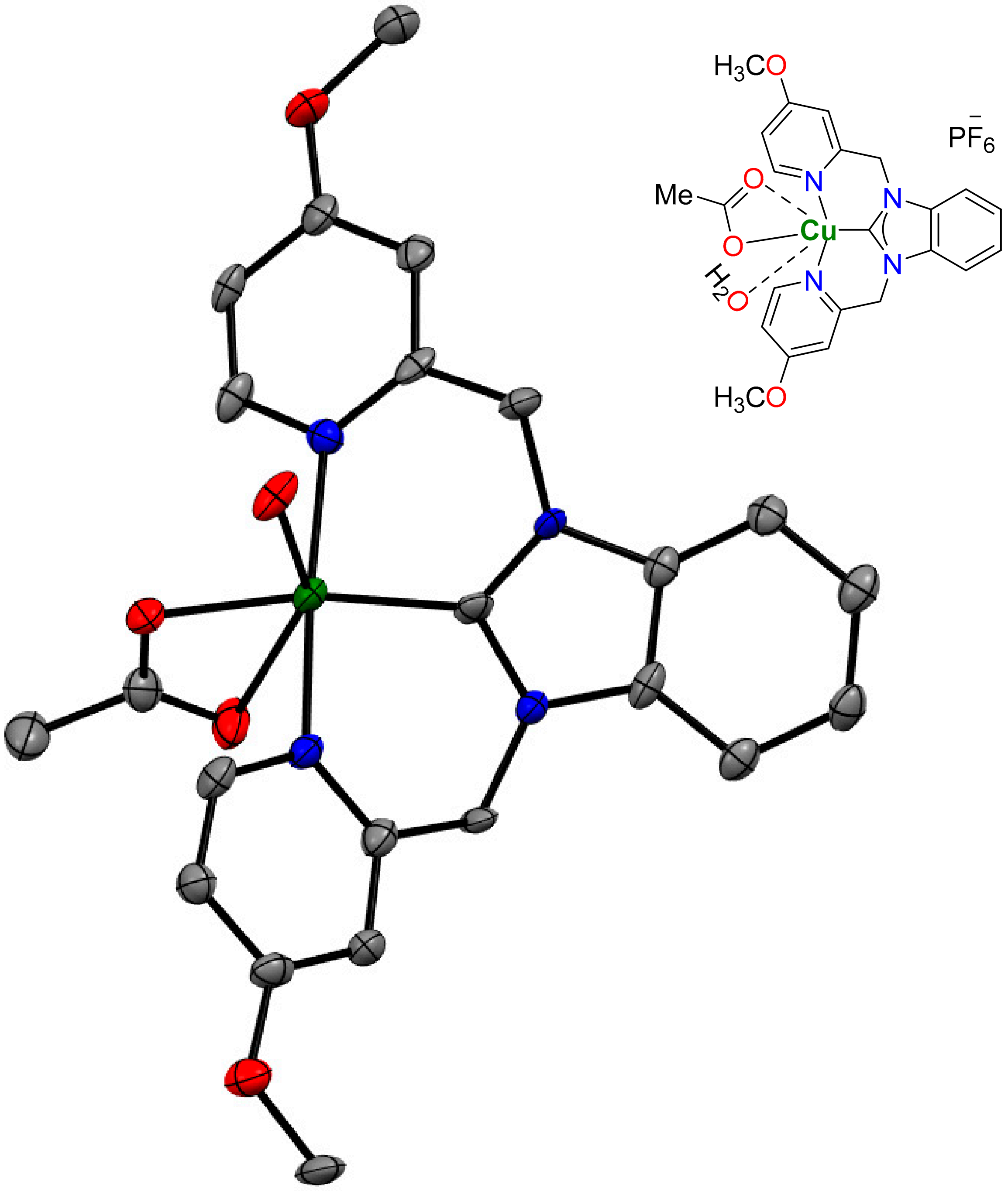

Table S2. Crystal data and structure refinement for **Cu^{2+}bPymBI-Ome [CCDC: 2313921]**

**Chemical formula**	$C_{23}H_{25}CuF_6N_4O_5P$
**Formula weight**	645.98 g/mol
**Temperature**	100(2) K
**Wavelength**	1.54178 Å
**Crystal size**	(0.105 x 0.147 x 0.253) mm^3
**Crystal system**	Triclinic
**Space group**	P -1
**Volume**	1328.10(9) $Å^3$
**Z**	2
**Density (calculated)**	1.615 g/cm^3
**Absorption coefficient**	2.505 mm^{-1}
**F(000)**	658
**Theta range for data collection**	4.06 to 66.55°
**Index ranges**	-13<=h<=11, -13<=k<=13, -14<=l<=14
**Independent reflections**	4509 [R(int) = 0.0851]
**Coverage of independent reflections**	96.1%
**Absorption correction**	Multi-Scan
**Max. and min. transmission**	0.7790 and 0.5700
**Function minimized**	$\Sigma w(F_o^2 - F_c^2)^2$
**Data / restraints / parameters**	4509 / 0 / 371
**Goodness-of-fit on F^2**	1.285
**Final R indices**	3681 data; $I>2\sigma(I)$ R1 = 0.1080, wR2 = 0.2062
	all data R1 = 0.1349, wR2 = 0.2212
**Weighting scheme**	$w=1/[\sigma^2(F_o^2)+15.3181P]$ where $P=(F_o^2+2F_c^2)/3$
**Largest diff. peak and hole**	0.687 and -0.726 $eÅ^{-3}$
**R.M.S. deviation from mean**	0.137 $eÅ^{-3}$

Figure S48. XRD of compound **Cu^{2+}bPymBI-Cl [CCDC: 2313923]**

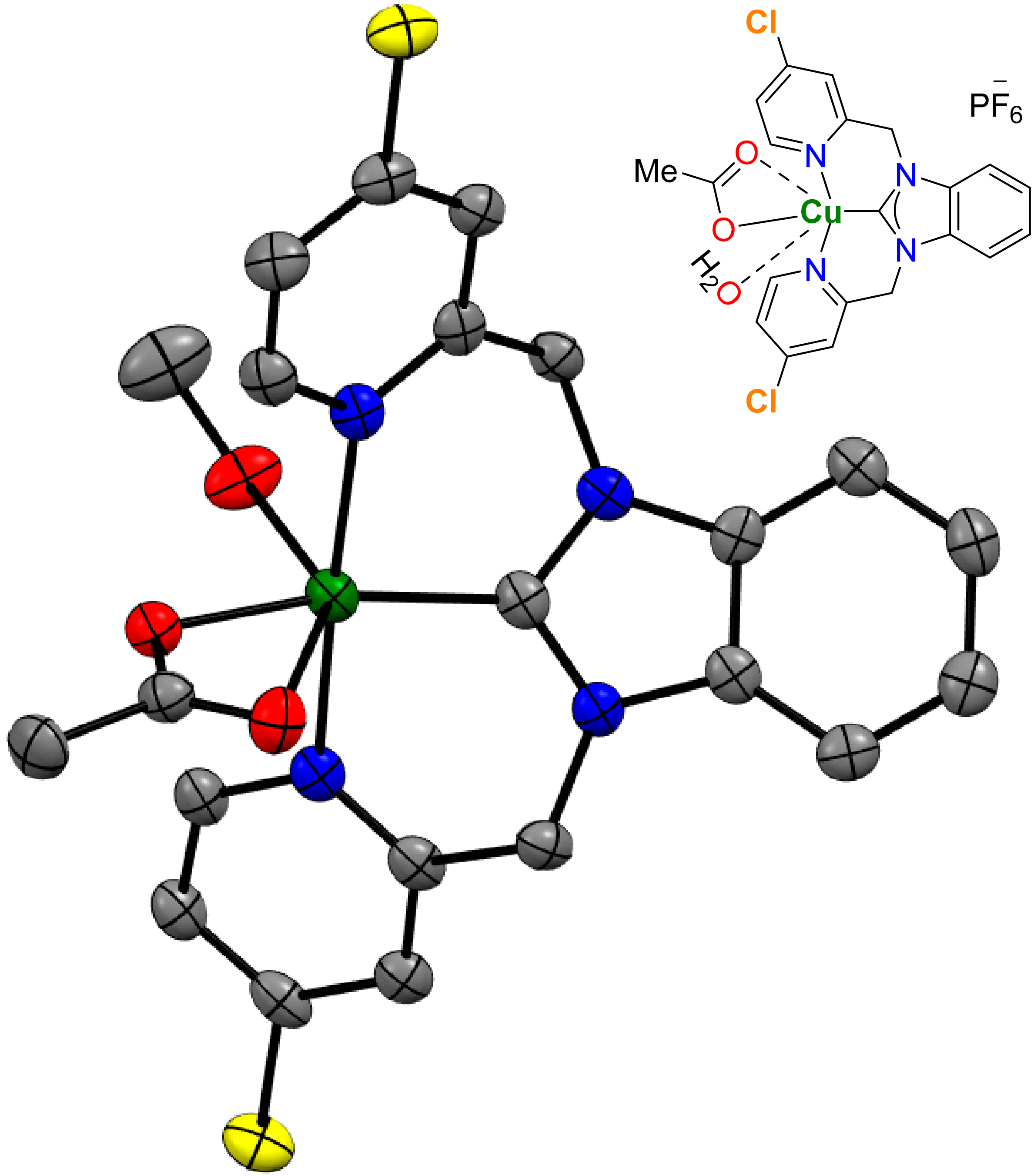

Table S3. Crystal data and structure refinement for **Cu^{2+}bPymBI-Cl [CCDC: 2313923]**

Empirical formula	$C_{22}H_{21}Cl_2CuF_6N_4PO_3$
Formula weight	668.84
Temperature/K	160(2)
Crystal system	triclinic
Space group	P-1
a/Å	7.1423(5)
b/Å	12.9101(11)
c/Å	14.7968(14)
α/°	73.002(8)
β/°	78.728(7)
γ/°	80.116(6)
Volume/Å^3	1270.0(2)
Z	2
ρ_{calc}g/cm^3	1.749
μ/mm^{-1}	1.212
F(000)	674.0
Crystal size/mm^3	(0.65 × 0.35 × 0.25)
Radiation	MoKα (λ = 0.71073)
2Θ range for data collection/°	4.972 to 54.206
Index ranges	$-9 \leq h \leq 9$, $-16 \leq k \leq 16$, $-18 \leq l \leq 18$
Reflections collected	18456
Independent reflections	5546 [R_{int} = 0.0721, R_{sigma} = 0.0633]
Data/restraints/parameters	5546/0/358
Goodness-of-fit on F^2	1.053
Final R indexes [I>=2σ (I)]	R_1 = 0.0649, wR_2 = 0.1646
Final R indexes [all data]	R_1 = 0.0846, wR_2 = 0.1774
Largest diff. peak/hole / e Å^{-3}	1.45/-1.11

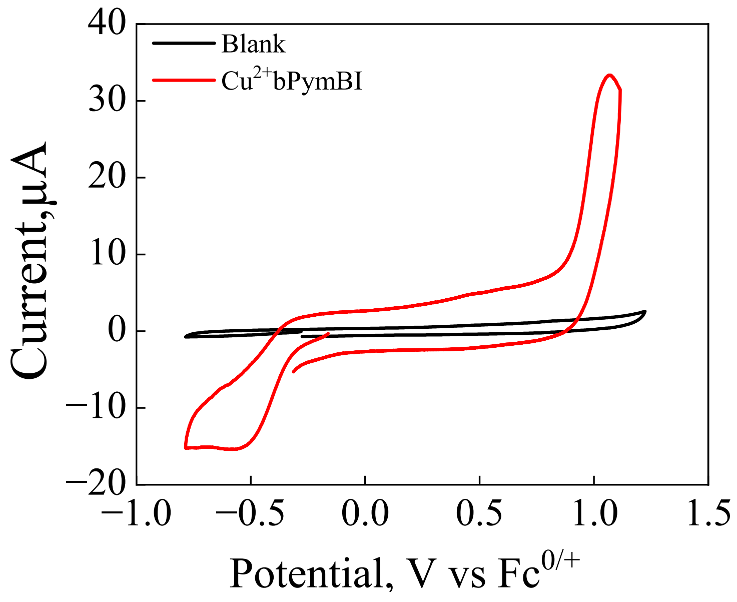

Figure S49. CV spectrum of compound **Cu^{2+}bPymBI**

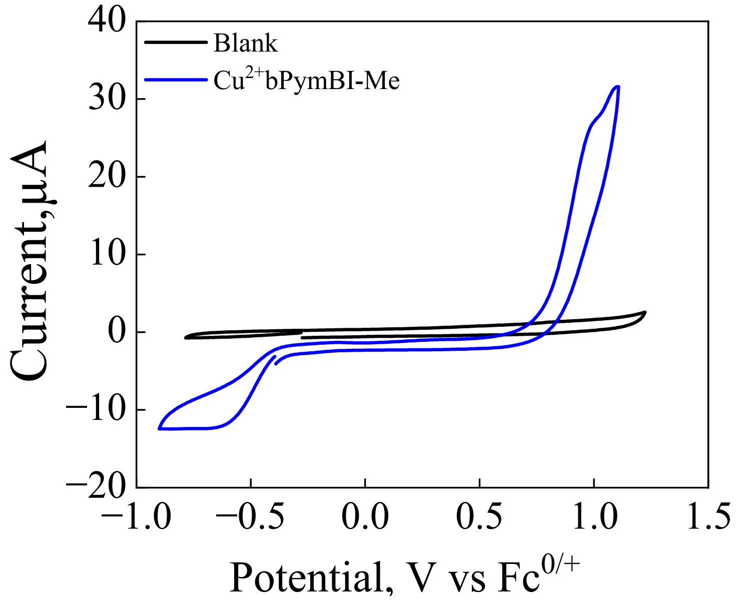

Figure S50. CV spectrum of compound **Cu^{2+}bPymBI-Me**

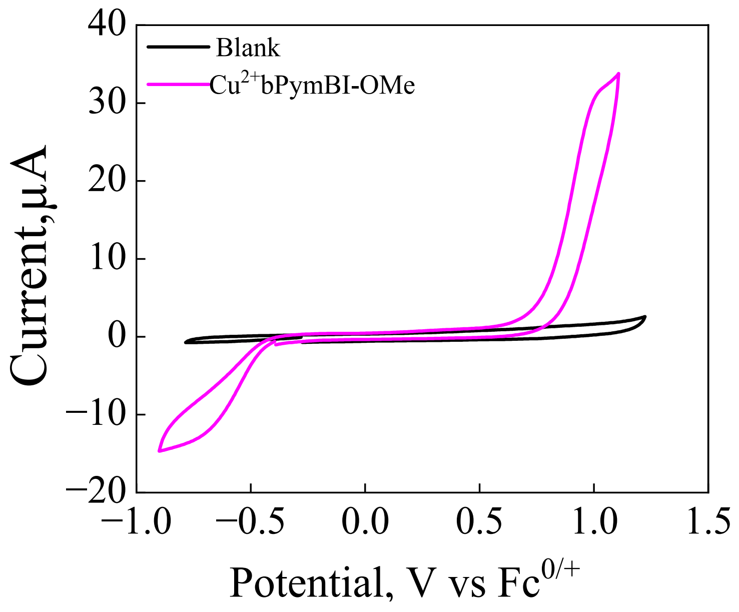

Figure S51. CV spectrum of compound **Cu^{2+}bPymBI-OMe**

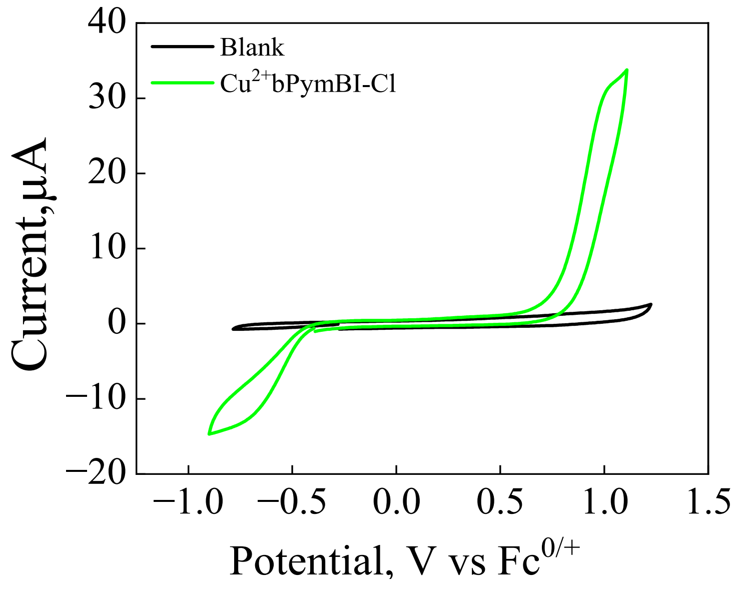

Figure S52. CV spectrum of compound **Cu^{2+}bPymBI-Cl**

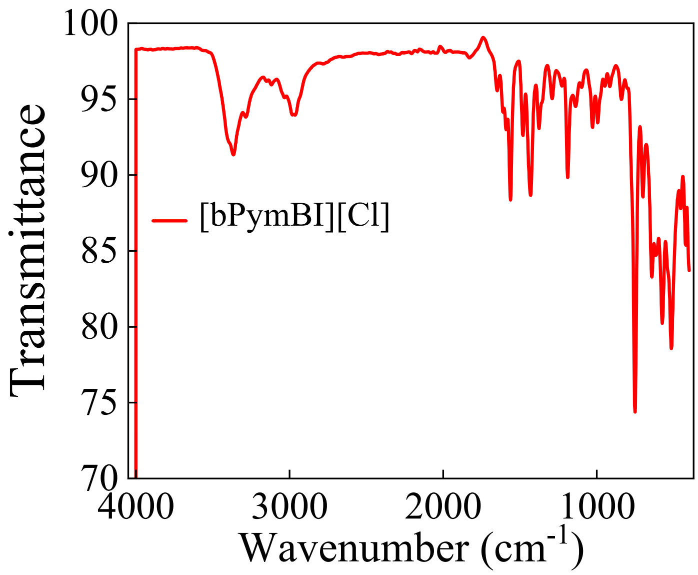

Figure S53. FT-IR spectrum of compound [**bPymBI**][**Cl**]

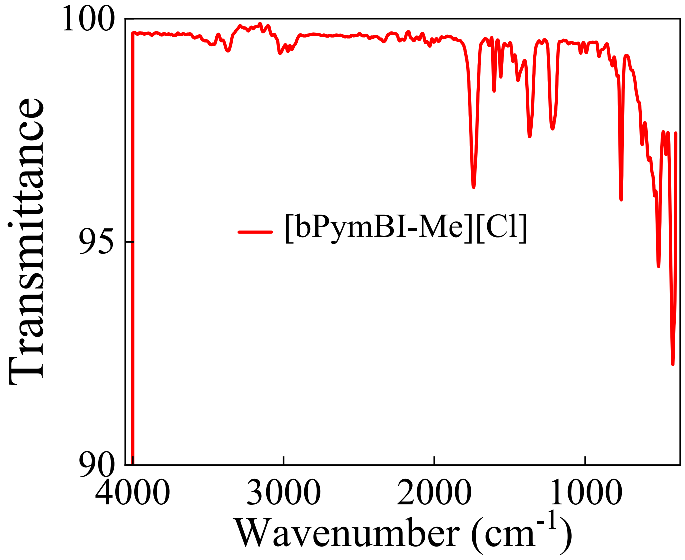

Figure S54. FT-IR spectrum of compound [**bPymBI-Me**][**Cl**]

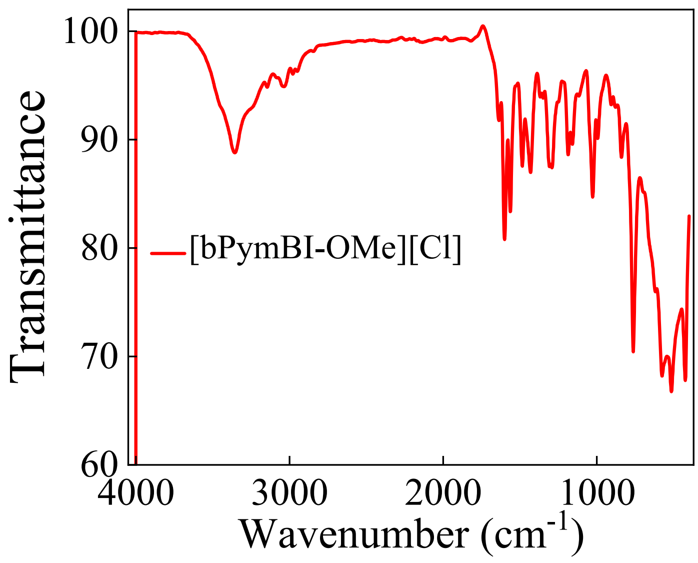

Figure S55. FT-IR spectrum of compound [**bPymBI-OMe][Cl]**

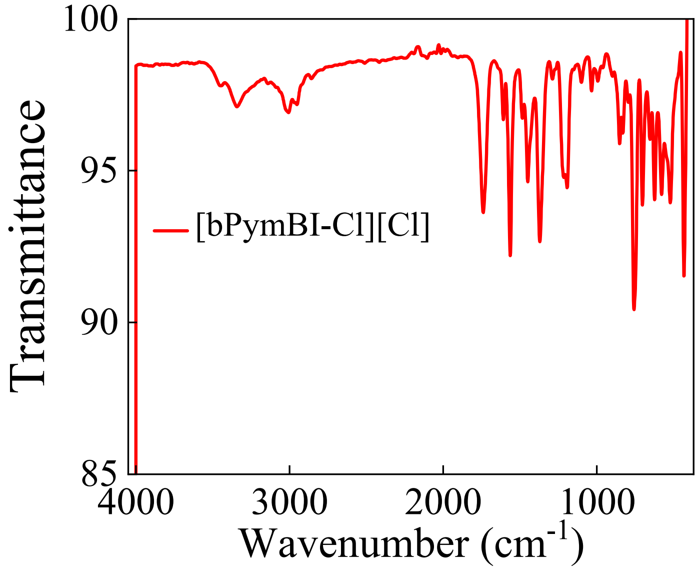

Figure S56. FT-IR spectrum of compound [**bPymBI-Cl][Cl]**

Table S4. EA for **Cu^{2+}bPymBI** Complexes

% Composition of Elements	Cu^{2+}bPymBI	Cu^{2+}bPymBI-Me	Cu^{2+}bPymBI-OMe	Cu^{2+}bPymBI-Cl
C [%] calculated	44.8	46.5	44.3	40.3
C [%] experimental	42.1	46.7	44.2	40.4
N [%] calculated	9.1	8.7	8.3	8.2
N [%] experimental	9.2	9.0	8.5	8.4
H [%] calculated	4.6	5.0	4.8	3.8
H [%] experimental	3.9	4.1	3.8	3.1

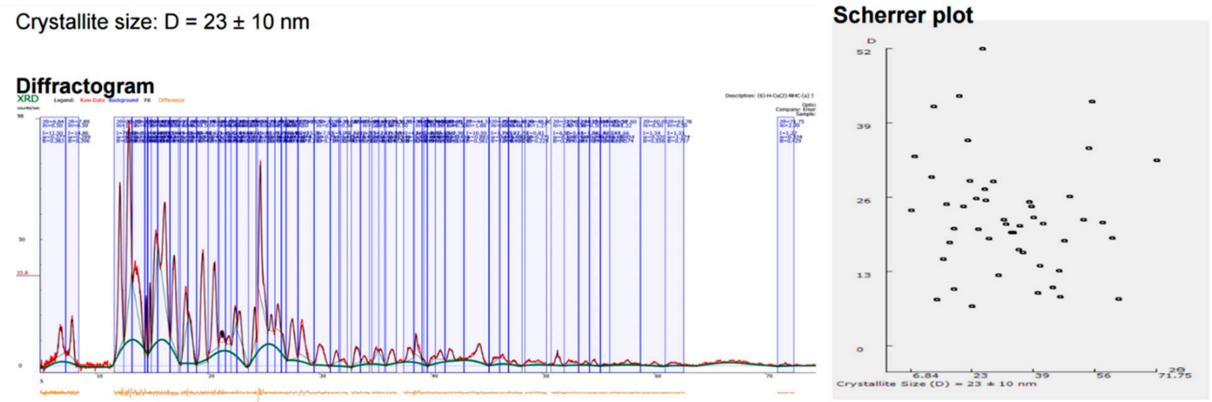

Figure S57. PXRD data of compound **Cu^{2+}bPymBI**

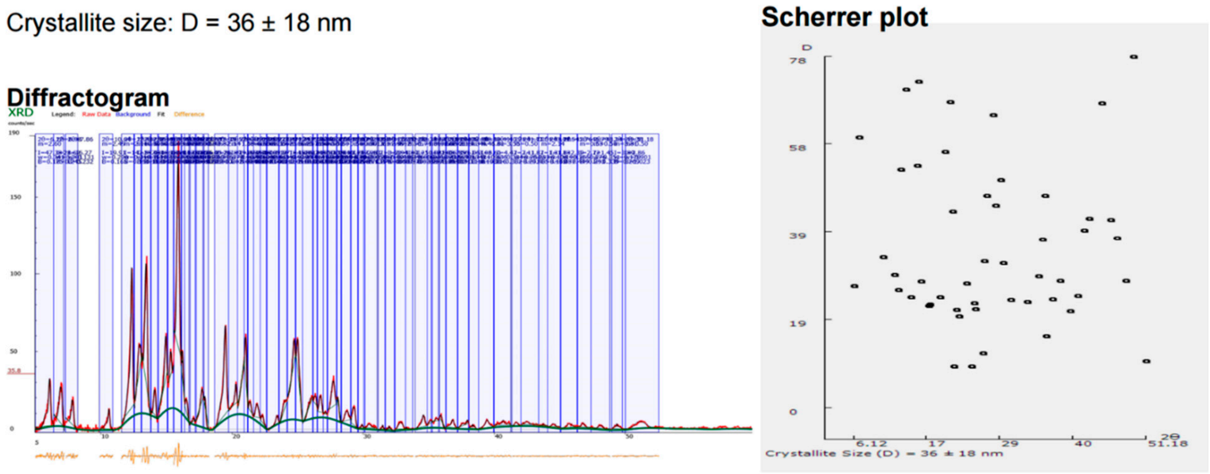

Figure S58. PXRD data of compound **Cu^{2+}bPymBI-Me**

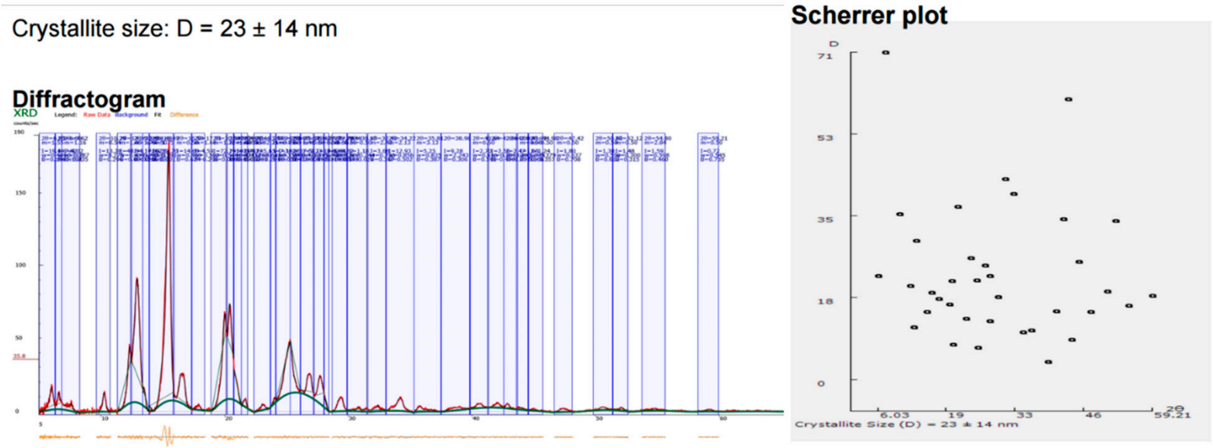

Figure S59. PXRD data of compound **Cu^{2+}bPymBI-OMe**

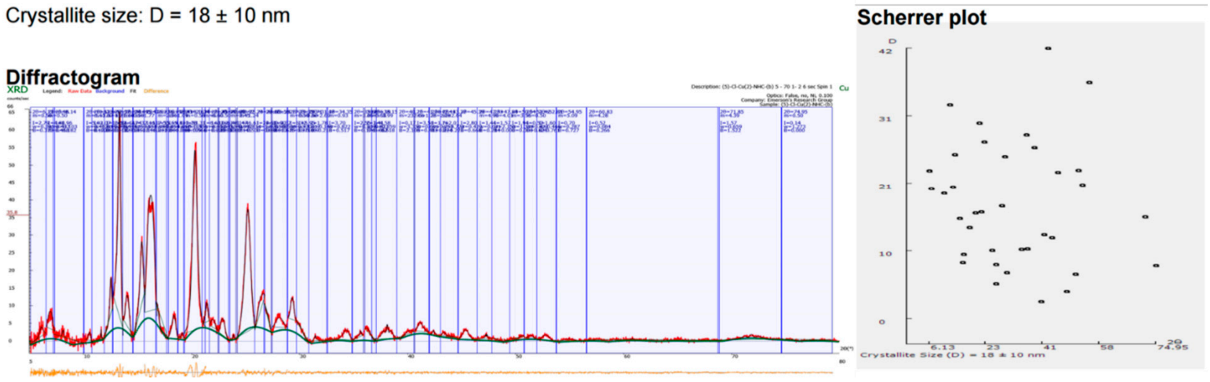

Figure S60. PXRD data of compound **Cu^{2+}bPymBI-Cl**

References

(1) Saint Program included in the package software: APEX4 v2022.10.0.

(2) Sadabs, Blessing R.H. (1995), Acta Cryst. A51. 33-38; Blessing, R. H. (1995) An empirical correction for absorption anisotropy. Acta Crystallographica Section A Foundations of Crystallography, 51 (1). 33-38; Multi-Scan (SADABS); Krause and al., 2015)

(3) SHELXT-Integrated space-group and crystal-structure determination Sheldrick, G. M. Acta Crystallogr., Sect. A 2015, A71, 3-8.

(4) SHELXTL Sheldrick, G. M. Ver. 2018/3. Acta Crystallographica. Sect C Structural Chemistry 71, 3 - 8.

(5) APEX4 v2022.10.0, AXS Bruker program.

(6) Sharma, M., Adhikari, B., Awoyemi, R. F., Perkins, A. M., Duckworth, A. K., Donnadieu, B., Wipf, D. O., Stokes, S. L., Emerson, J. P. (2022). Copper(II) NHC Catalyst for the Formation of Phenol from Arylboronic Acid. *Chemistry, 4*(2), 560-575.

(7) Cope, J. D., Sheridan, P. E., Galloway, J. C., Awoyemi, R. F., Stokes, S. L., & Emerson, J. P. (2020, December 11). Synthesis and Characterization of a Tetradentate, N-Heterocyclic Carbene Copper(II) Complex and Its Use as a Chan–Evans–Lam Coupling Catalyst. *Organometallics, 39*(24), 4457-4464.
